# Prefrontal-raphe-habenular circuit drives fear memory recall and updating

**DOI:** 10.1038/s41467-026-76574-5

**Published:** 2026-08-12

**Authors:** Boldizsár Zsolt Balog, Albert M. Barth, Krisztián Zichó, Abel Major, Zsuzsanna Bardóczi, János Brunner, Charlotte Seng, Márta Jelitai, Katalin E. Sos, Réka Z. Sebestény, András Szőnyi, Nikolas Karalis, Áron Orosz, Eszter Sipos, Erik Misák, György Cserey, Andreas Lüthi, Viktor Varga, Csaba Földy, János Szabadics, Gábor Nyiri

**Affiliations:** 1 Systems & Integrative Neuroscience Research Group, HUN-REN Institute of Experimental Medicine, Budapest, Hungary; 2 Tamás Roska Doctoral School of Sciences and Technology, Pázmány Péter Catholic University, Budapest, Hungary; 3 Laboratory of Subcortical Modulation, HUN-REN Institute of Experimental Medicine, Budapest, Hungary; 4 Semmelweis University Doctoral School, János Szentágothai Neurosciences Divisions, Budapest, Hungary; 5 Semmelweis University Doctoral School, Dental Research Division, Budapest, Hungary; 6 Laboratory of Cellular Neuropharmacology, HUN-REN Institute of Experimental Medicine, Budapest, Hungary; 7 Laboratory of Neural Connectivity, Brain Research Institute, Faculties of Medicine and Science, University of Zürich, Zürich, Switzerland; 8 Laboratory for Cellular Mechanisms of Learning and Memory, Friedrich Miescher Institute, Basel, Switzerland; 9 Paris Brain Institute, Inserm, Sorbonne Université, CNRS, APHP, Hôpital de la Pitié Salpêtrière, Paris, France; 10 Faculty of Information Technology and Bionics, Pázmány Péter Catholic University, Budapest, Hungary

**Keywords:** Fear conditioning, Neural circuits

## Abstract

Fear memory recall is a vital defensive response, yet its underlying neuronal mechanism remains incompletely understood. Here, we identify an effective fear recall pathway. We found that hippocampal and amygdalar memory centers directly target a group of medial prefrontal cortex (mPFC) neurons, which are necessary for fear recall and directly target vesicular glutamate transporter 2 (vGluT2)-expressing neurons in the pontine median raphe region (MRR^vGluT2^ neurons) in mice. We found that MRR^vGluT2^ neurons are necessary for recalling contextual and cued fear memories, they exhibit scalable fear-dependent activity, bidirectionally regulate depression-like behaviors, and show prolonged activity after a fearful experience. During fear memory recall, MRR^vGluT2^ neurons activate the lateral habenula (LHb), a region implicated in major depression. MRR^vGluT2^ neurons are electrophysiologically and genetically diverse, and present in rodent and primate species. The identified mPFC–MRR–LHb pathway critically mediates fear memory recall, providing potential therapeutic targets for fear-related disorders.

## Abstract (web summary)

Fear memory recall is a vital defensive response, but its neuronal mechanism remains unclear. Here, authors show a brainstem pathway that links memory centers to negative emotional states during fear memory recall, showing how the brain reactivates fear in the absence of danger.

## Introduction

Fear is an emotional state that serves as a vital defensive mechanism, enabling both humans and animals to respond adaptively to threats. While human fear often involves conscious awareness, its foundational components are also observable in animals as fear states, reflected in stereotypical defensive behaviors^1–4^. Decades of research on fear memory recall and its updating during extinction have established a canonical tripartite circuit model involving the amygdala, hippocampus, and prefrontal cortex^5–13^. According to this model, during contextual and cued fear recall, highly processed, multisensory information is conveyed to the hippocampus and amygdala, respectively. These regions subsequently recruit the prefrontal cortex to orchestrate appropriate fear memory recall and extinction^6,8,14–19^. Although some models have already incorporated additional thalamic and midbrain structures^5,20–23^, even the most recent models remain focused on forebrain-based circuits^24,25^.

During fear memory recall, behavioral responses closely resemble those observed during initial fear conditioning, suggesting that animals may partially re-experience the subjective aversive state associated with the original fearful event^26–28^. Brain regions engaged in this aspect of fear memory recall likely activate aversion-related neural circuits, including the lateral habenula^29,30^. Although evidence suggests that certain cognitive functions and associated network oscillations are regulated by pontine brainstem nuclei^31–35^, including the median raphe region^31,36–39^, the pontine brainstem has traditionally not been associated with fast-acting and direct roles in memory recall.

Memory recall and learning are distinct computational processes: whereas learning requires the integration of ongoing nociceptive signals with contextual or cued information to form new associations, recall reactivates a pre-existing representation and re‑evaluates its affective valence in the absence of the original aversive stimulus. Failure to distinguish these processes may obscure dedicated circuits that reassign negative valence during recall. We therefore hypothesized that a brainstem-mediated pathway might play a recall‑specific role by reinstating the aversive state associated with a memory, rather than encoding its content.

Here, we discovered a medial prefrontal cortex (mPFC) - median raphe region (MRR) – lateral habenula (LHb) fear memory recall pathway. We demonstrate that vesicular glutamate transporter 2 (vGluT2)-expressing neurons in the evolutionarily ancient pontine MRR project to several fear recall-related regions and are necessary for fear memory recall. We found that vGluT2-expressing MRR neurons (MRR^vGluT2^ neurons) update both contextual and cued fear memories and exhibit a scalable, fear-dependent activity. We show that during fear memory recall MRR^vGluT2^ neurons activate the LHb, a critical hub involved in setting motivational states that has been implicated in the development of major depression^40,41^. The connection between MRR^vGluT2^ neurons and the LHb is particularly relevant, because we found that MRR^vGluT2^ neurons can bidirectionally modulate depression symptoms and show prolonged activation after stress. We also show that MRR^vGluT2^ neurons are conserved across rodents and primates. Therefore, to facilitate future translational efforts, we have characterized the physiological and genetic heterogeneity of MRR^vGluT2^ neurons using the single-cell mRNA sequencing. Furthermore, we also investigated how cortical memory circuits activate MRR^vGluT2^ neurons to recall fear memories. We hypothesized that mPFC recalls the negative emotional aspect of aversive memories by activating the MRR^vGluT2^ neurons directly. We identified a specific population of mPFC projection neurons, which directly target the MRR^vGluT2^ neurons that in turn target the LHb to evoke the aversive experience. We demonstrated that MRR-targeting mPFC neurons (mPFC^→MRR^ neurons) are both necessary and sufficient for fear recall and also represent negative experiences. We found that mPFC^→MRR^ neurons receive direct inputs from the hippocampus and amygdala, regions critical for contextual and cued fear memory recall, respectively. This suggests that mPFC^→MRR^ neurons connect memory circuits and the fear-state-broadcasting MRR^vGluT2^ neurons that target the LHb, which plays a critical role in major depression^40,41^. Thus, the mPFC→MRR→LHb pathway we describe here represents a link between forebrain memory networks and brainstem valence centers that is specifically engaged during recall. Because maladaptive recall of aversive memories underlies conditions such as PTSD and depression, elucidating this recall-specific circuit provides a conceptual framework for understanding how negative affect is reactivated and offers potential therapeutic targets.

## Results

### MRR^vGluT2^ neurons are necessary for recalling contextual fear memories

**Fig. 1:**
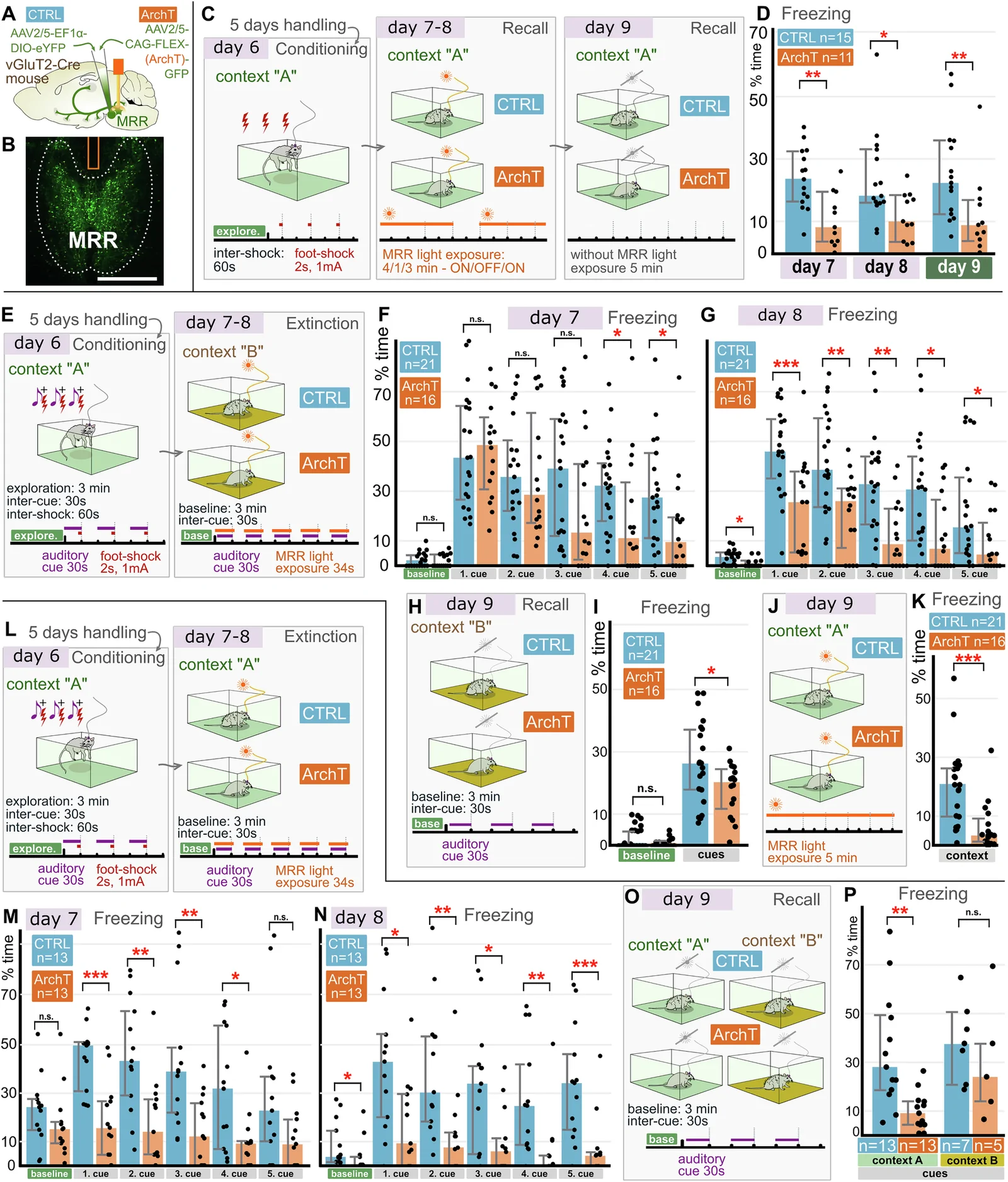
MRR^vGluT2^ neurons are necessary for fear memory recall. **A** Cre-dependent AAV injection into the MRR of vGluT2-Cre mice. Control mice (CTRL) received AAV2/5-EF1α-DIO-eYFP; inhibited mice (ArchT) received AAV2/5-CAG-FLEX-ArchT-GFP. Optic fibers (OF, orange) were implanted over the MRR. **B** Fluorescent image shows MRR^vGluT2^ eYFP expression (green) and optic fiber placement (orange). Scale bar, 500 μm. **C** Contextual fear conditioning timeline. Days 1-5: handling; day 6: 3 foot-shocks (1 mA, 2 s) in context “A”; days 7–8: 8 min in context “A” with 7 min OF light; day 9: 5 min in context A without OF light. **D** The percentage of time the mice spent with freezing behavior. ArchT mice (orange) showed significantly lower freezing than CTRL mice (blue) on days 7-9 (n_CTRL_=15, n_ArchT_=11). **E** Cued fear conditioning in a separate cohort (same preparation as A-B). Timeline: days 1-5 handling; day 6: context “A” with 3 auditory cues (30 s) co-terminated with foot-shocks (1 mA, 2 s); days 7–8: novel context “B” with 5 auditory cues and OF light (34 s) starting 2 s pre-cue. **F, G** ArchT mice (orange) displayed significantly lower post-cue freezing on days 7-8 (n_CTRL_=21, n_ArchT_=16). **H** Day 9, context “B” with 3 auditory cues without OF light; **I** ArchT mice showed significantly lower freezing than CTRL mice (nCTRL= 21, nArchT=16). **J** 5 minutes in context “A” with continuous OF light. **K** ArchT mice showed significantly lower freezing than CTRL mice (n_CTRL_=21, n_ArchT_=16). **L** Combined cued and contextual fear conditioning in a separate cohort. Paradigm identical to (E) for days 1–8, except days 7–8 used context “A” instead of “B”. **M, N** ArchT mice (orange) displayed significantly lower freezing on days 7-8 (n_CTRL_=13, n_ArchT_=13). **O** Day 9, context “A” then novel context “B”, with 3 auditory cues without OF light; **P** ArchT freezing was significantly lower than CTRL in context “A”, but not “B” (context “A”: n_CTRL_=13, n_ArchT_=13; context “B”: n_CTRL_=7, n_ArchT_=5). For statistical details, see Table S1. Graphs show medians and interquartile ranges. For reconstructed optic fiber localization see Fig. S16. Source data are provided as a Source Data file.

Previous studies suggested the importance of MRR in cognitive and negative experience processing^31,36,37,39^. Therefore, to test whether MRR^vGluT2^ neurons are necessary for contextual fear memory recall, we inhibited MRR^vGluT2^ neurons optogenetically using adeno-associated viruses (AAV) during fear recall in mice. To achieve that, we injected Cre-dependent, inhibitory archaerhodopsin (ArchT)-encoding AAV (ArchT mice) or control AAV (CTRL mice) into the MRR of vGluT2-Cre mice (Fig. 1A). We then implanted optic fibers above the MRR (Fig. 1B). After handling for 5 days, on day 6, mice received mild foot-shocks in context “A” (Fig. 1C), during which the groups showed no significant difference in fear behavior (freezing behavior, Figure S1A). On the following two days (7 and 8), we placed mice back in context “A”, delivered light to the MRR and found that, compared to the CTRL mice, ArchT mice showed a significantly lower contextual fear response (Fig. 1D, Table S1). This showed that MRR^vGluT2^ neurons are necessary for contextual fear memory recall.

On day 9, we placed mice back into context “A” without light exposure via the optic fiber (Fig. 1C) and we found that the significantly diminished fear behavior persisted in ArchT mice (Fig. 1D, Table S1) without further optogenetic intervention even a day later.

In the same experiment, inhibition of MRR^vGluT2^ neurons did not alter spontaneous locomotor activity, further supporting their specific role in fear recall rather than any non-specific influence on general locomotor functions (Fig. S1B, C, Table S1).

These data demonstrated that the activity of MRR^vGluT2^ neurons during fear memory recall serves as a critical reference point for subsequent recalls of the same memory and their inhibition has a persistent effect on fear memory.

### MRR^vGluT2^ neurons are necessary for cued fear memory recall

We next investigated whether MRR^vGluT2^ neurons are also essential for recalling the second major type of fear memory, known as cued fear. We prepared another cohort of mice that received the same optogenetic actuators and underwent the same surgical procedures as described above (Fig. 1A, B). Then, after handling, on day 6, mice received 30 seconds-long auditory cues co-terminated with mild foot-shocks in context “A” (Fig. 1E). Both groups showed normal acute fear behavior (freezing behavior, Figure S1D). On the following two days, we placed mice in a novel context “B” and presented the same auditory cue 5 times each day to recall cued fear memories. Simultaneously, we exposed the MRR^vGluT2^ neurons to light via the optic fibers, during the cues (Fig. 1E). We found that, compared to CTRL mice, light-inhibited ArchT mice showed significantly lower cued fear responses during the memory recall episodes (Fig. 1F, G, Table S1). Fear extinction was also enhanced in ArchT mice (Fig. 1F, G, Table S1). This showed that MRR^vGluT2^ neurons are necessary also for cued fear memory recall.

On day 9, we placed mice back into context “B” without optic fiber light exposure and presented the original auditory cues (Fig. 1H). We found that the lower freezing behavior in ArchT mice persisted (Fig. 1I, Table S1). Later, we placed mice back into the conditioning context “A” with optic fiber light exposure (Fig. 1J) and found that ArchT mice showed significantly less contextual fear recall (Fig. 1K, Table S1).

Furthermore, we demonstrated that the place aversion - induced by the activity of MRR^vGluT2^ neurons^31^ - can be associated with an auditory cue, and that this aversive association can later be recalled by re-exposing mice to the same auditory cue (Figure S1F–I). This showed that even the memory of the previous activation of MRR^vGluT2^ neurons (recalled using an auditory cue) can induce place aversion (Fig. S1F–I, Table S1).

Additionally, the role of MRR^vGluT2^ neurons was further tested during simultaneous recall of contextual and cued components of fear memories. Mice were prepared and conditioned the same way as in the previous cued fear conditioning experiment, but this time, cue recall with optic fiber light exposure was tested in the conditioning context “A” (Fig. 1A, B, L). After handling, on day 6, mice received mild foot-shocks in context “A” (Fig. 1L), during which the groups showed no significant difference in fear behavior (freezing behavior, Fig. S1E). We found that on day 7 and day 8, compared to CTRL mice, inhibited ArchT mice showed significantly lower fear responses during the cues in context “A” (Fig. 1M, N, Table S1). All these data demonstrated that MRR^vGluT2^ neurons are necessary for both cued and contextual fear memory recall.

On day 9, we first placed mice back into context “A” and then into a novel context “B” without light exposure (Fig. 1O) and found that the lower freezing persisted in ArchT mice for context “A”, but no such difference could be detected in context “B” (Fig. 1P). In summary, we found that precise inhibition of MRR^vGluT2^ neurons during memory recall accelerated the extinction of both contextual and cued fear memories, and this effect persisted for at least a day.

### Fear memory recall activates MRR^vGluT2^ neurons in a scalable manner, calcium imaging confirms

**Fig. 2:**
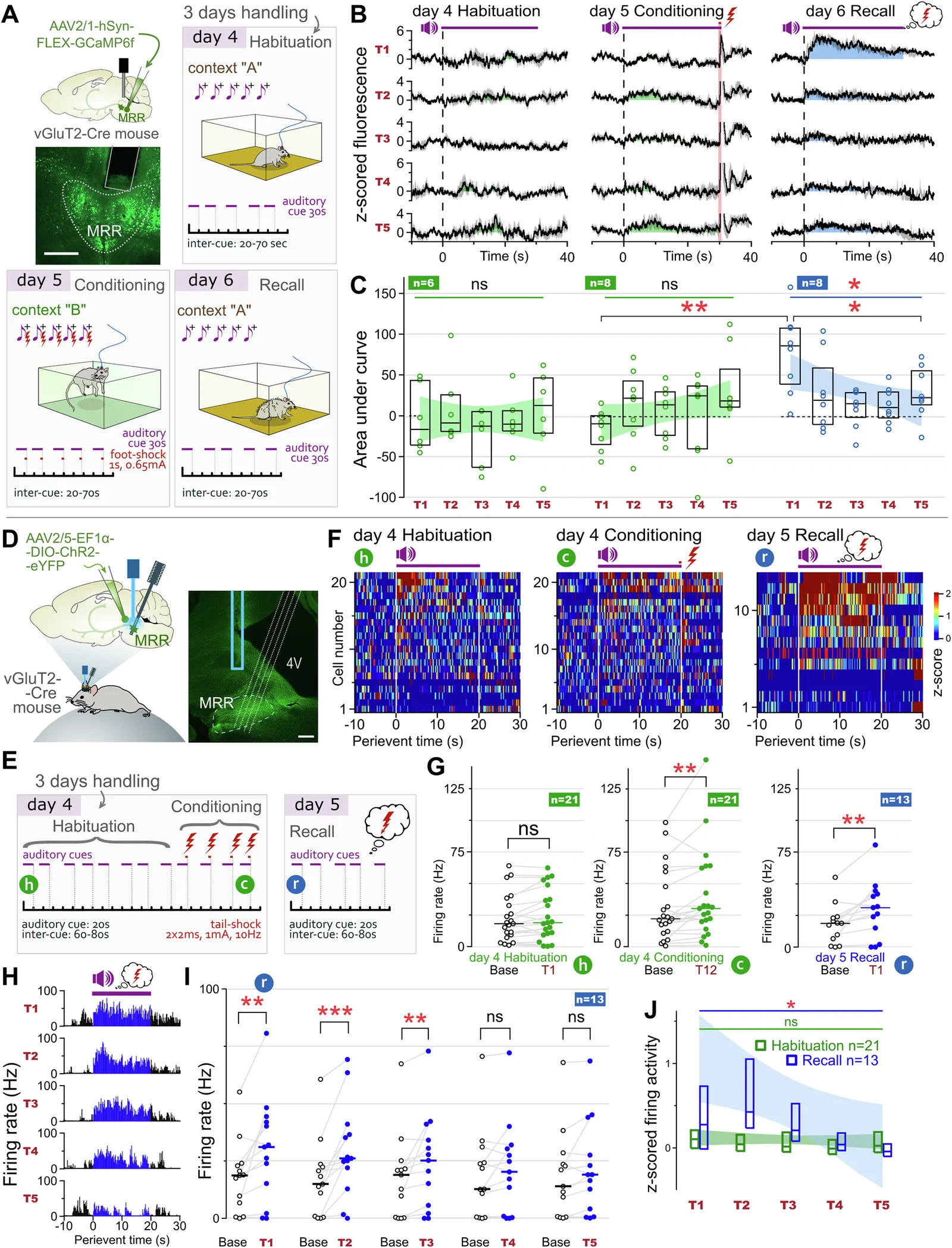
Scalable activation of MRR^vGluT2^ neurons during cued fear memory recall and extinction. **A** Cre-dependent GCaMP6f AAV injection (green) into and optic fiber implantation (grey) over the MRR of vGluT2-Cre mice. Scale bar, 500 μm. Cued fear conditioning: days 1-3 (Handling); day 4 (Habituation): context “A” with five auditory cues (30 s); day 5 (Conditioning): novel context “B” with five cues co-terminated with foot shocks (0.65 mA, 1 s), day 6 (Recall): five cues in context “A”. **B** Averaged peristimulus z-scored fluorescent signals of MRR^vGluT2^ neurons (mean ± SEM). Area-under-the-curve are shaded with green and blue. **C** Corresponding AUC boxplots during cues (habituation n_mice_ = 6; conditioning n_mice_ = 8; recall n_mice_ = 8; shading indicates 95% confidence interval of linear regression). Fear recall significantly increased MRR^vGluT2^ activity (Conditioning T1 vs. Recall T1) followed by progressive reduction (Recall T1 vs. T5), reflected by a significant negative regression. **D** For in vivo head-fixed electrophysiology, Cre-dependent, ChR2 AAV was injected into the MRR of vGluT2-Cre mice. During recordings, a silicon probe was inserted into, and an optic fiber was lowered over the MRR. Fluorescent image shows eYFP expression (green), optic fiber position (blue) and probe tracks (sagittal section). Scale bar, 500 μm. **E** Head-fixed cued fear conditioning: days 1-3: handling; day 4: mice were head-fixed and acutely recorded. After optogenetical tagging, eight 20-s auditory cues (Habituation) were followed by four cue-tail shock pairings (2 × 2 ms, 1 mA; Conditioning). Day 5 (Recall), same head-fixed recording with five cues. **F** Color scaled, z-scored peristimulus firing rates of tagged MRR^vGluT2^ neurons. **G** Average baseline and cue firing rates during Habituation (left, T1; n_units_ = 21; 6 mice), Conditioning (middle, T12; n_units_ = 21; 6 mice) and Recall (right, T1; n_units_ = 13; 4 mice, horizontal lines - medians). **H** Peristimulus firing histograms of a representative tagged MRR^vGluT2^ neuron during Recall (day 5, T1-5). **I** Firing rates of all tagged MRR^vGluT2^ neurons during Recall. Activity increased during the first three cues, but not the last two (n_units_=13; 4 mice). **J** Z-scored responses showed significant negative regression during Recall (n_units_=13; 4 mice), but not Habituation (n_units_=21; 6 mice; 95% confidence intervals). For statistical details, see Table S2 and Table S3. Graphs show medians and interquartile ranges unless specified otherwise. Source data are provided as a Source Data file.

Given our findings above that MRR^vGluT2^ neurons are required for fear memory recall, we investigated their activity during fear memory recall and their potential adaptation during fear extinction. To test this using fiber photometry calcium imaging experiments, we injected Cre-dependent, GCaMP-encoding AAV into the MRR of vGluT2-Cre mice and then implanted optic fibers above the MRR (Fig. 2A). After handling, on day 4, mice were habituated to five 30-second-long auditory cues (T1-5) in context “A”. On day 5, mice were placed in a novel context “B”, where they received five auditory cues, each co-terminated with an aversive foot-shock. On day 6, we placed mice back in context “A” and presented the auditory cues 5 times to investigate the activity of MRR^vGluT2^ neurons during repeated fear recall. Each day, neuronal activity-dependent (and control isosbestic) fluorescence signals were recorded using fiber photometry (Fig. 2A). We found that during fear memory recall, the first auditory cue resulted in significantly larger MRR^vGluT2^ neuronal activation compared to the first auditory cue before fear associations were formed (i.e. day 6-T1 vs day 5-T1, Fig. 2B, C, Table S2). This showed that MRR^vGluT2^ neurons are significantly activated during cued fear memory recall. Furthermore, the activity of the MRR^vGluT2^ neurons during repeated recalls showed a significant negative regression across the 5 cue-presentations (Fig. 2C, Table S2) and a significantly reduced MRR^vGluT2^ neuronal response during the last cue compared to the first cue (day 6-T5 vs day 6-T1). To demonstrate successful fear extinction, we analyzed baseline-corrected freezing levels and found a significant negative regression across consecutive cue presentations in these experiments as well, accompanied by a decline in MRR^vGluT2^ neuronal activity (Fig. S2C). None of these differences were present in the control isosbestic signals (Fig. S2A, B, Table S2). These data highlight a precise and scalable fear memory recall-related activation of MRR^vGluT2^ neurons.

### Fear memory recall activates MRR^vGluT2^ neurons in a scalable manner, electrophysiology confirms

We next examined the activation dynamics of MRR^vGluT2^ neurons during fear memory recall with higher temporal resolution using in vivo electrophysiological experiments. We injected Cre-dependent, channelrhodopsin 2 (ChR2)-encoding AAV into the MRR of vGluT2-Cre mice (Fig. 2D). After handling, mice were placed into a head-fixed setup, where MRR^vGluT2^ neurons were identified by optogenetic tagging and their activity was recorded using multichannel silicone probes (Fig. S2F, G). On day 4, neural activity was recorded both during the habituation of the mice to the auditory cues and during cued fear conditioning (auditory cue + aversive tail-shocks). On day 5, mice were presented with the same auditory cues alone to trigger fear memory recall (Fig. 2E). We found that cue presentations before cue-shock association on day 4 did not alter MRR^vGluT2^ neuronal activity (“h” in Fig. 2F, G). However, following three cue–shock pairings, their activity increased significantly in response to the final cue presentation (“c” in Fig. 2F, G). During recall, on day 5, the activity of MRR^vGluT2^ neurons significantly increased at the presentation of the first cue (“r” in Fig. 2E, F, G), accompanied by pupil dilatation (Fig. S2D, E) indicating that the cue-shock pairings were successful. On day 5, during the 5 cue presentations (Fig. 2H), the activity of MRR^vGluT2^ neurons showed a significant negative regression (Fig. 2J, Table S3) and we found that only the first 3 cues resulted in a significant activation of MRR^vGluT2^ neurons (T1-3, Fig. 2I, Table S3). These findings not only indicate that the MRR^vGluT2^ neurons are precisely activated during fear recall, but they also show that individual MRR^vGluT2^ neurons follow the progress of fear extinction in a scalable manner. This is important because MRR^vGluT2^ neurons project to the aversion-inducing LHb^31^.

### MRR^vGluT2^ neurons innervate negative memory recall-related brain areas

To identify the brain regions targeted by MRR^vGluT2^ neurons, we performed anterograde viral axonal tracings (Fig. S3). Although some of these projections have already been documented^31^, here we identified all MRR^vGluT2^ projections to multiple memory and emotional regulation-related brain regions (Fig. S3A–C). The most prominent targets were the LHb, the ventral tegmental area, the medial septum, the vertical limb of the diagonal band of Broca and the nucleus incertus. Other areas were also targeted including the interpeduncular nucleus, the dorsal raphe, the lateral hypothalamus, the lateral preoptic area, the pontine central gray and the subventricular tegmental nucleus.

The LHb, a key region in aversive information processing and negative prediction, receives a major input from the MRR^vGluT2^ neurons^31^. Therefore, we specifically investigated whether individual LHb-targeting MRR^vGluT2^ neurons (MRR^vGluT2→LHb^ neurons) form divergent projections to other brain regions. By labeling MRR^vGluT2→LHb^ neurons selectively (using a combination of retrograde and anterograde AAV labeling from the LHb, Fig. S3D), we found that individual MRR^vGluT2→LHb^ neurons can innervate all major target regions listed above (Fig. S3D–F). No substantial difference was observed in the regional distribution pattern of axonal projections originating from MRR^vGluT2^ and MRR^vGluT2→LHb^ neurons (Fig. S3G). This suggests that MRR^vGluT2→LHb^ neurons simultaneously influence several components of the memory and emotional circuits.

### MRR^vGluT2→LHb^ neurons control LHb activity during fear memory recall

**Fig. 3:**
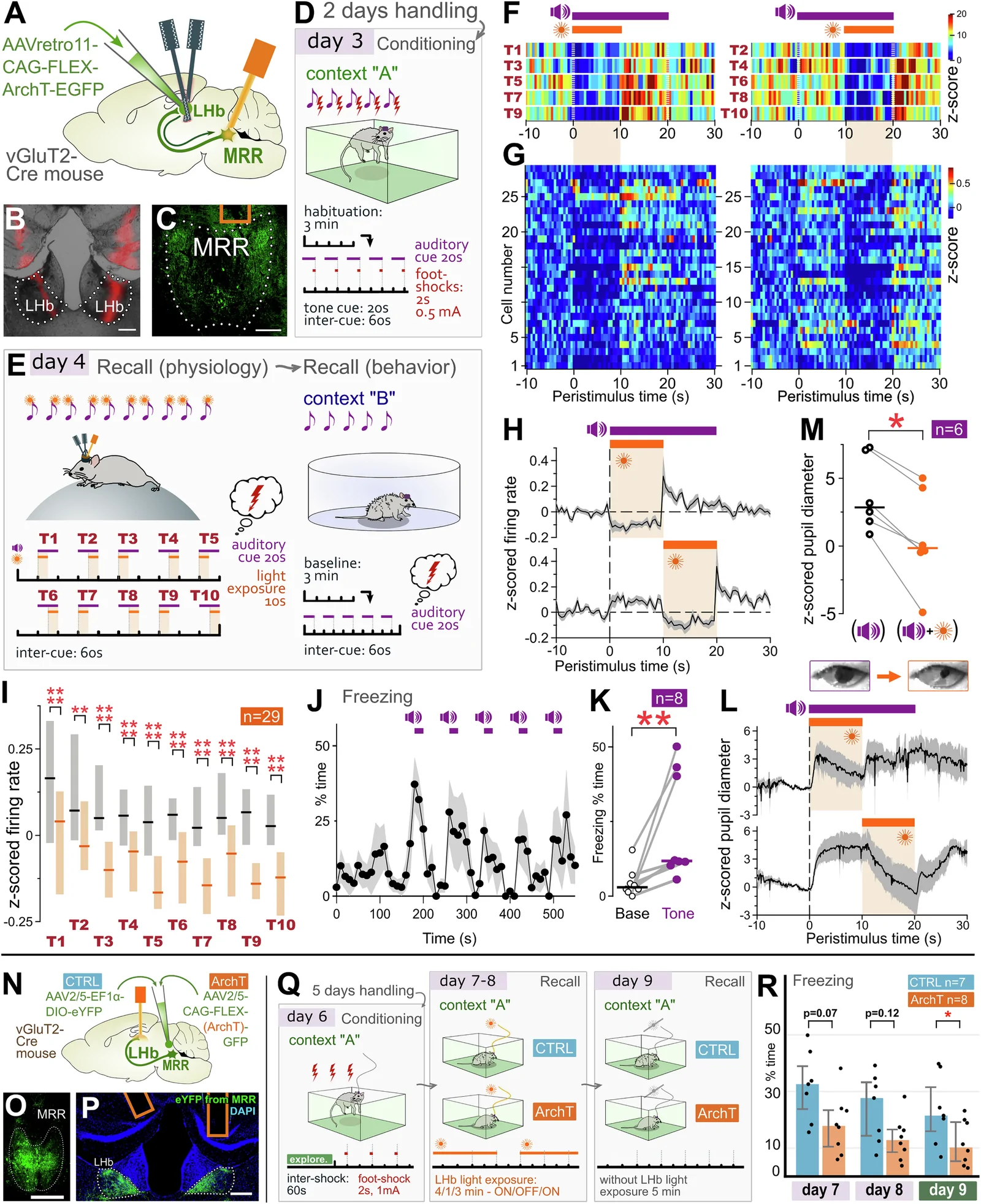
MRR^vGluT2→LHb^ neurons drive the activation of LHb neurons during cued fear memory recall. **A** Bilateral injection of Cre-dependent, retrograde ArchT AAV into the LHb of vGluT2-Cre mice; optic fiber over the MRR, silicon probes in LHb bilaterally. **B** Fluorescent DiI probe tracks (red) in the LHb; Scale Bar, 500 μm. **C** Optic fiber position (orange) over EGFP-expressing MRR^vGluT2→LHb^ neurons (green); Scale Bar, 200 μm. **D**,** E** Illustration of cued fear conditioning paradigm as described in the Results. **F–I** Significant decrease in activity (z-scored firing rate) of LHb neurons during cued fear memory recall, upon optogenetic inhibition of MRR^vGluT2→LHb^ neurons (n_units_=29, 8 mice). **F** Color-scaled z-scored peristimulus firing rates of a representative LHb neuron across recall trials (T1–T10), separated by inhibition timing (left: first half; right: second half of cue). **G** Color-scaled average z-scored firing rates of all recorded LHb neurons show reduced activity during MRR^vGluT2→LHb^ inhibition. **H** Corresponding averaged z-scored peristimulus firing histograms (mean ± SEM; top: first-half inhibition, bottom: second-half inhibition). The rebound activation following the end of optogenetic stimulation (upper trace) did not affect the conclusion of the analysis (see Methods). **I** Z-scored firing rates of inhibited and non-inhibited recall periods across trials. **J**,** K** One hour after head-fixed recordings, freely moving cued fear recall in novel context B showed increased freezing (mean ± SEM; n_mice_ = 8). **L** Average z-scored pupil-diameter traces during head-fixed fear recall, separated by inhibition timing (mean ± SEM). **M** Inhibition reduced pupil diameter (n_mice_=6). **N**,** O** Cre-dependent AAV injection into the MRR of vGluT2-Cre mice (CTRL: eYFP AAV; ArchT: ArchT-GFP AAV). Scale bar, 500 μm. **P** Optic fiber implantation above the bilateral LHb (green axons; orange optic fibers; blue DAPI). Scale bar, 200 μm. **Q** Contextual fear conditioning: 1-5 days: handling; day 6: three foot-shocks (2 s, 1 mA) in context “A”; days 7–8: 8 min in context “A” with 7 min OF light (8 mW); day 9: 5 min in context A without OF light. **R** ArchT mice displayed significantly reduced freezing compared to CTRL mice on day 9 (n_CTRL_=7, n_ArchT_ = 8). Graphs show medians and interquartile ranges unless specified otherwise. For statistical details, see Table S3. For reconstructed optic fiber localization see Fig. S16. Source data are provided as a Source Data file.

In light of the central role of LHb in fear processing^29,42^ and our findings on the necessity and scalability of the activity of MRR^vGluT2^ neurons during fear memory recall, we hypothesized that the MRR^vGluT2^ → LHb pathway constitutes a key circuit element mediating fear memory recall. To test this, we performed in vivo electrophysiological recordings of LHb neurons, combined with optogenetic inhibition of MRR^vGluT2→LHb^ neurons, during fear memory recall. We injected retrogradely spreading, inhibitory ArchT-encoding, Cre-dependent AAV11^43^, into the LHb of vGluT2-Cre mice (Fig. 3A-C). After handling, on day 3, freely moving mice received five 20-second-long auditory cues, co-terminated with aversive foot-shocks in context “A” (Fig. 3D). On day 4, mice were placed into a head-fixed setup, where we used optic fibers (inserted above MRR) to inhibit MRR^vGluT2→LHb^ neurons, while we recorded the activity of LHb neurons using multichannel silicon probes bilaterally (Fig. 3B, C, E). Mice then received the same (ten 20 s) auditory cues (T1–T10), aligned with 10-second inhibition of MRR^vGluT2→LHb^ neurons, and timed either to the first or second half of each cue (Fig. 3E). We found that inhibiting MRR^vGluT2→LHb^ neurons during fear memory recall significantly reduced the activity of a subgroup of LHb neurons (29/153) (Fig. 3F–I). Additionally, 27.3% (9/33) of the CS-activated units exhibited decreased firing rates during optogenetic inhibition of MRR^vGluT2→LHb^ neurons during CS presentation, whereas the remaining units were not significantly modulated. Furthermore, inhibition of MRR^vGluT2→LHb^ neurons during fear memory recall led to a significant reduction in pupil diameter (Fig. 3L, M), further supporting the notion that suppressing the activity of MRR^vGluT2^ neurons has a broad inhibitory effect on the recall of negative experiences. Following the head-fixed experiment (Fig. 3E), we confirmed that the cued-fear association was retained in these mice under freely moving conditions (Fig. 3J–K).

### MRR→LHb pathway is necessary for fear memory updating

To directly test the role of MRR^vGluT2→LHb^ neurons in fear memory recall, we performed an optogenetic experiment, where we specifically inhibited their axonal fibers in the LHb. We injected Cre-dependent, inhibitory ArchT-encoding AAV (ArchT mice) or control AAV (CTRL mice) into the MRR of vGluT2-Cre mice (Fig. 3N, O) and bilaterally implanted optic fibers above the LHb (Fig. 3P). After handling, on day 6, all mice received foot-shocks in context “A”. Both groups displayed normal acute fear responses (freezing behavior, Fig. S4H–L). On days 7-8, mice were returned to context “A” and were exposed to light through the optic fibers (Fig. 3Q). Compared to CTRL mice, ArchT mice showed a tendency toward reduced contextual fear responses (Fig. 3R, Table S1). Finally, on day 9, mice were returned to context “A” without optic fiber light exposure, and ArchT-expressing mice exhibited significantly reduced freezing behavior (Fig. 3R, Table S1). This showed that selective inhibition of the MRR^vGluT2^ → LHb pathway significantly enhances fear memory extinction.

### Chronic stress induces prolonged activation of MRR^vGluT2^ neurons

**Fig. 4:**
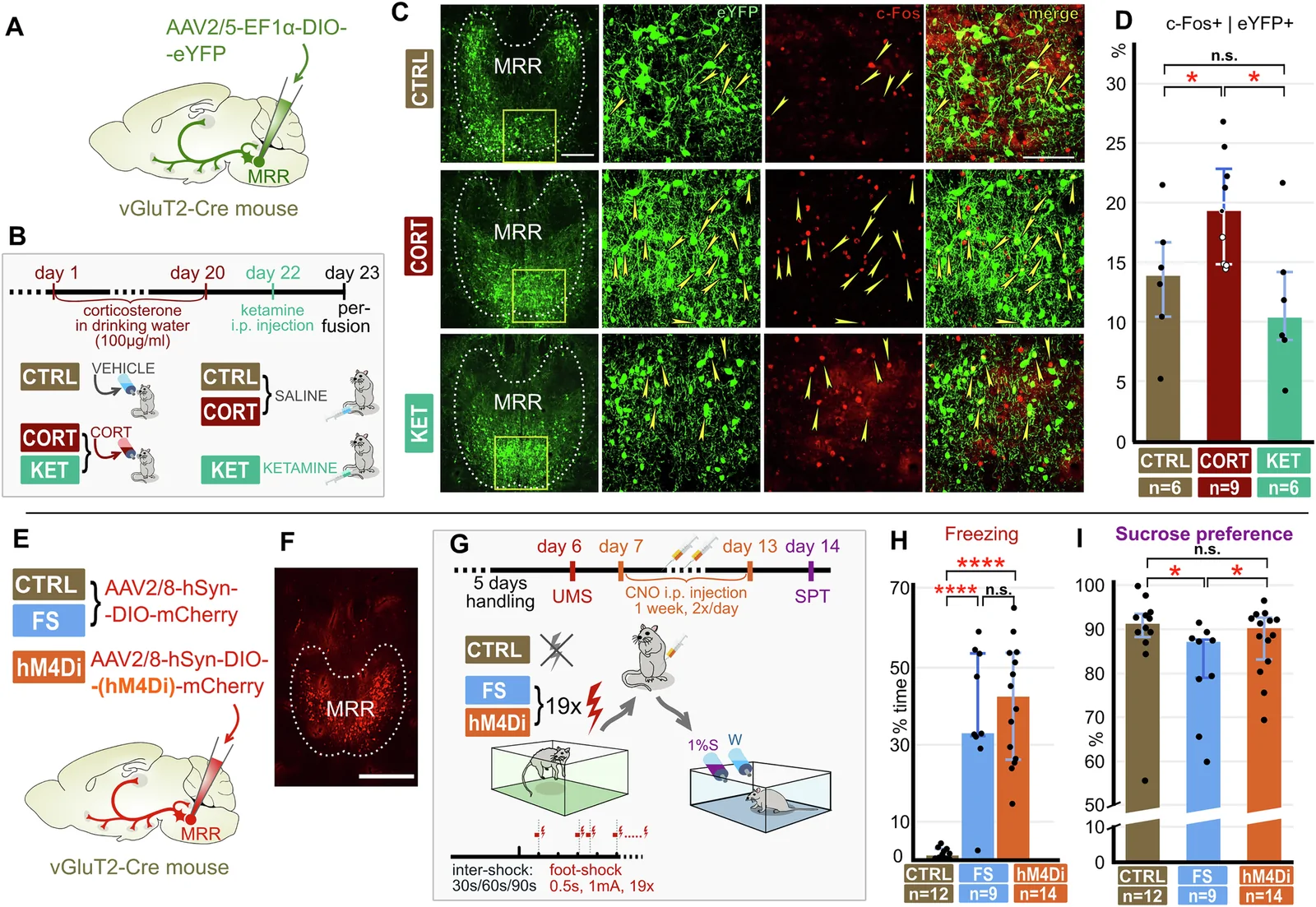
MRR^vGluT2^ neurons are chronically activated in depression-like state but their inhibition can alleviate depression-like symptoms. **A** Cre-dependent AAV was injected into the MRR of vGluT2-Cre mice. **B** Illustration of the corticosterone-induced model of depression as described in the Results. CORT and KET mice received 100 µg/ml corticosterone in their drinking water for 3 weeks, control mice (CTRL) did not. Day 22: KET mice received 10 mg/kg ketamine, CTRL and CORT mice received saline intraperitoneally (i.p.). Day 23: mice were perfused. **C** Representative confocal laser scanning images reveal higher expression levels of c-Fos protein (red) in MRR^vGluT2^ neurons (green) in a CORT mouse (middle row) compared to a CTRL mouse (top row) or a KET mouse (bottom row) mouse. Yellow squares indicate areas magnified on the right images. Scale bar, 200 μm for left column, 100 μm for right 3 columns. **D** Significantly higher c-Fos expression in MRR^vGluT2^ neurons of CORT, but not KET mice compared to CTRL mice (n_CTRL_=6, n_CORT_ = 9, n_KET_ = 6). **E** Cre-dependent AAVs were injected into the MRR of vGluT2-Cre mice. Control mice (CTRL) and foot-shock mice (FS) were injected with control AAV, hM4Di mice were injected with inhibitory, CNO-sensitive hM4Di-expressing AAV. **F** Fluorescent image shows mCherry expression (red) in the MRR. Scale bar, 500 μm. **G** Illustration of the unpredictable mild shock (UMS) model of depression as described in the Results. FS and hM4Di mice received 19 mild foot-shocks (0.5 s, 1 mA) with pseudorandom inter-shock-interval (30 s or 60 s or 90 s), CTRL mice only explored the chamber. 1 mg/kg CNO was injected i.p. twice per day for a week. **H** Freezing time percentage was significantly higher in FS and hM4Di groups compared to CTRL mice during the UMS (n_CTRL_=12, n_FS_ = 9, n_hM4Di_ = 14). **I** The UMS induced significantly lower sucrose preference in FS mice compared to CTRL mice but chronic chemogenetic inhibition of MRR^vGluT2^ neurons restored normal phenotype in hM4Di mice (n_CTRL_=12, n_FS_ = 9, n_hM4Di_ = 14). For statistical details, see Table S1. Graphs show medians and interquartile ranges. Source data are provided as a Source Data file.

After chronic stress, LHb neurons contribute to the development of depression-like behaviors^40,41,44,45^. Additionally, major depressive disorder in humans is associated with a bias toward recalling negative memories^46^, however ketamine, a breakthrough therapeutic for depression, can alleviate depression symptoms, specifically acting on LHb-related circuits^41,44,47,48^. We hypothesized that MRR^vGluT2^ neurons may contribute to the prolonged recall of negative memories associated with depression. Therefore, we investigated MRR^vGluT2^ neuronal activity in a chronic stress-induced model of depression and following a single ketamine treatment. First, we labeled MRR^vGluT2^ neurons by injecting Cre-dependent, eYFP encoding AAV into the MRR of vGluT2-Cre mice (Fig. 4A). After one week, “corticosterone-mice” (CORT) and “ketamine-mice” (KET) received 100 µg/ml corticosterone in their drinking water [known to induce depression-like behavior^49^], while “control-mice” (CTRL) received only vehicle, for three weeks (approximately 500 µg/day, 20 mg/kg/day, Fig. 4B). The following day, all mice received clean water for a day. Then, KET mice received a single dose of ketamine (10 mg/kg), whereas CTRL and CORT mice received saline intraperitoneally. We generated two independent cohorts of these experiments under identical experimental conditions. In the first cohort, we performed a sucrose preference test [SPT,^50^]. We confirmed that CORT mice developed significant anhedonia (lower sucrose preference), a core symptom of depression, and that a single ketamine treatment in KET mice restored normal behavior in SPT (Fig. S4A, B, Table S1) as expected^40,44,48,51^. This validated our model and the significant effect of the ketamine treatment.

In the second cohort, the mouse brains were fixed one day after the ketamine/saline injection (Fig. 4B) that is two days after the final corticosterone treatment. Then, we assessed the acute activity of MRR^vGluT2^ neurons by investigating c-Fos expression, which can signal neuronal activation within the 20–90 min preceding brain fixation^52^. We found that CORT mice exhibited a significantly higher ratio of c-Fos-expressing MRR^vGluT2^ neurons compared to CTRL mice (Fig. 4C, D, Table S1). However, following ketamine treatment, we observed significantly fewer c-Fos-positive MRR^vGluT2^ neurons in KET mice compared to CORT mice, rendering the KET mice indistinguishable from CTRL mice. This suggests that ketamine treatment reduces MRR^vGluT2^ neuronal activity (Fig. 4C, D, Table S1).

This data also suggests that MRR^vGluT2^ neurons remain highly active even two days after a negative experience, which suggests that under stressful conditions, they may contribute to the development of depression-like behavior. Our results in KET mice also show that ketamine treatment significantly affects the activity of MRR^vGluT2^ neurons, which may play a significant role in the antidepressant effect of the ketamine treatment either directly or via their connections with the LHb.

### Chronic inhibition of MRR^vGluT2^ neurons relieves depression-like phenotypes

To determine whether MRR^vGluT2^ neurons are involved in the development of depressive phenotypes, we tested whether their chronic inhibition alleviates depression-related anhedonia. We injected Cre-dependent, inhibitory, modified human M4 muscarinic receptor (hM4Di)-encoding AAV (hM4Di mice) or control AAV [FS (only foot shocked) and CTRL (control) mice] into the MRR of vGluT2-Cre mice (Fig. 4E, F). Neuronal inhibition via hM4Di can be selectively induced using clozapine N-oxide (CNO). FS and hM4Di mice were subjected to the unpredictable mild stress (UMS) protocol^53^, which involved 19 mild foot-shocks delivered at pseudo-random inter-shock intervals. CTRL mice were exposed to the same context for an equivalent duration, but without receiving foot-shocks (Fig. 4G). We found that compared to CTRL mice, both FS and hM4Di mice showed significantly higher freezing levels, as expected (Fig. 4H, Table S1). Following UMS, mice received intraperitoneal injections of CNO twice daily, for one week. On the following day, the SPT (Fig. 4G) revealed that foot-shocked FS mice exhibited significantly greater anhedonia compared to CTRL mice, as expected. In contrast, the chronic inhibition of MRR^vGluT2^ neurons in hM4Di mice significantly ameliorated anhedonia, restoring sucrose preference to control levels (Fig. 4I, Table S1). These findings show that sustained inhibition of MRR^vGluT2^ neurons can rescue the depressive phenotype.

Furthermore, in a different set of experiments we also confirmed that a one week long chemogenetic activation of MRR^vGluT2^ neurons not only induced significant anhedonia [as previously suggested^31^], but also elicited learned-helplessness behavior, another core phenotype associated with depression (Fig. S4C–G, Table S1).

Together, these data strengthen the main conclusion that MRR^vGluT2^ neurons are not merely involved in acute fear responses, but act as a broader hub for negative affective memory recall and updating.

### MRR^vGluT2→LHb^ neurons comprise distinct subpopulations based on their electrophysiological properties

**Fig. 5:**
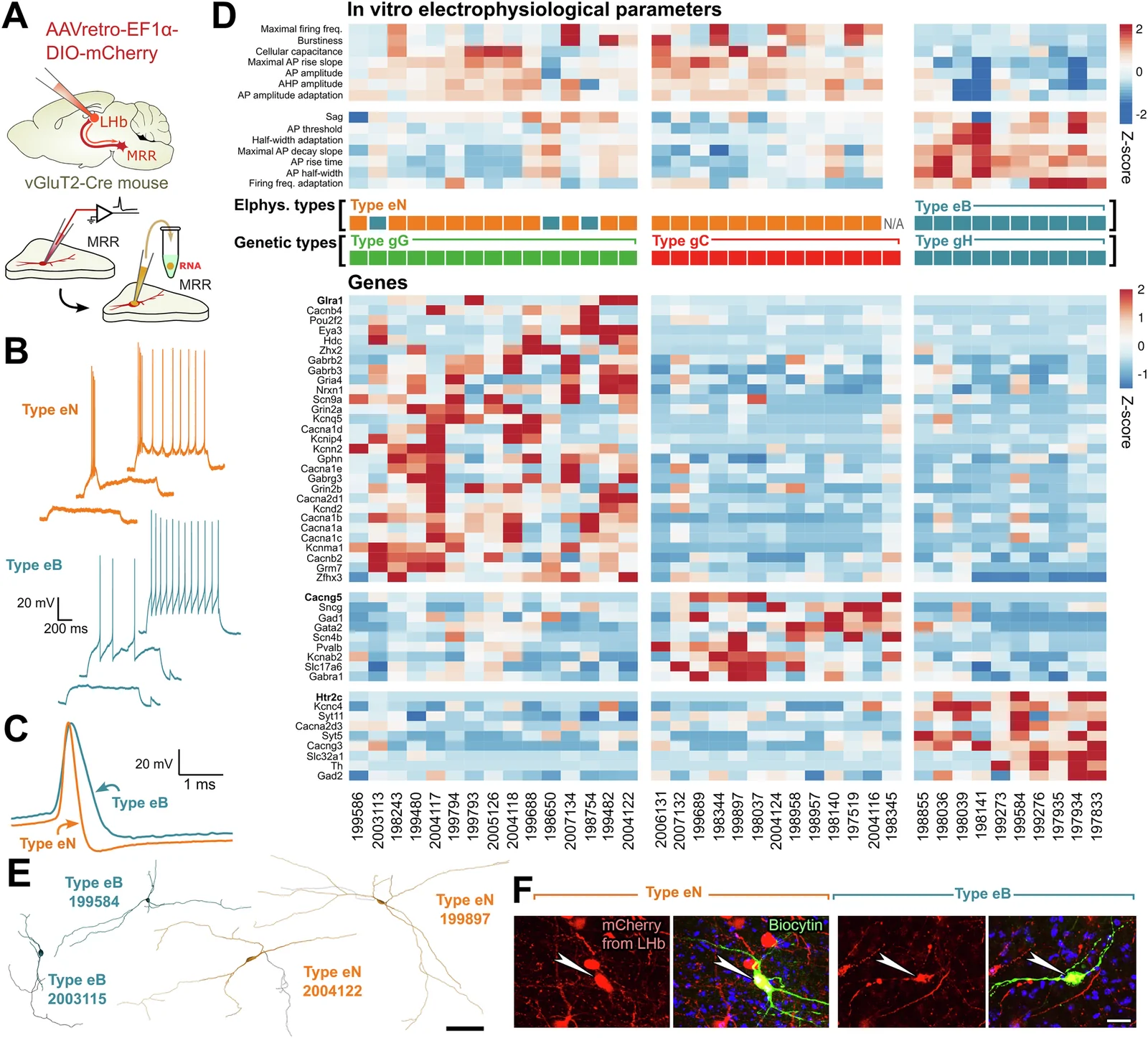
Electrophysiological and gene expression profiles of MRR^vGluT2→LHb^ neurons. **A** Cre-dependent, mCherry-encoding, retrograde AAV was injected into the LHb of vGluT2-Cre mice bilaterally to label MRR^vGluT2→LHb^ neurons. Labeled MRR^vGluT2→LHb^ neurons were characterized in in vitro patch clamp experiments and sampled for whole cell mRNA sequencing (Patch-seq). **B** Voltage responses from two representative MRR^vGluT2→LHb^ neurons evoked by gradually increasing current injections, which were used to characterize their firing properties that resulted in two major electrophysiological types: *Type eN* (electrophysiological type, Narrow spiker), *Type eB* (electrophysiological type, Broad spiker; see panel D, Fig. S5, Fig. S6 and Results for details). **C** Representative single action potentials, showing the distinct waveforms of the two neuron types. **D** Upper heatmaps show the z-scored differences in 14 electrophysiological parameters and lower heatmaps show differences in gene expressions among the MRR^vGluT2→LHb^ neurons. Between the two tables of heatmaps, color boxes represent clusters based on the electrophysiological and the genetic screening of MRR^vGluT2→LHb^ neurons. Hierarchical clustering of electrophysiological data revealed 2 subtypes. Hierarchical mRNA cluster analysis revealed 3 subtypes that we named based on the gene expression of which differed most significantly from the other two subtypes [*Type gG* (*glycine receptor alpha 1), Type gC (voltage-gated calcium channel auxiliary subunit gamma 5), Type gH (Htr2c – serotonin receptor 2c)]*. Individual neuron IDs are shown at the bottom of the heatmaps. For more electrophysiological, morphological, localization and genetic data see Figs. S5–9, Table S3, and Table S9-S10. **E** Four examples of 3-dimensionally reconstructed MRR^vGluT2→LHb^ neurons. Scale bar, 100 μm. **F** Fluorescent images show examples of recorded and biocytin-filled (green) retrogradely labeled (red) MRR^vGluT2→LHb^ neurons from both electrophysiological clusters. Scale bar, 20 μm.

Our anatomical data indicated that MRR^vGluT2→LHb^ neurons have diverse projections to multiple brain regions involved in aversive information processing. To better understand the potential heterogeneity of MRR^vGluT2→LHb^ neurons we performed in vitro electrophysiological recordings followed by single-cell PatchSeq mRNA sequencing^54^. For this, we labeled MRR^vGluT2→LHb^ neurons by injecting retrogradely spreading, Cre-dependent, mCherry-encoding AAV into the LHb of vGluT2-Cre mice. Then, the fluorescently labeled MRR^vGluT2→LHb^ neurons were patch clamped in acute slices to characterize their electrophysiological behavior. Neurons were filled with biocytin during the recordings for post hoc anatomical investigation (Fig. 5A). Hierarchical cluster analysis revealed two electrophysiologically distinct subtypes of MRR^vGluT2→LHb^ neurons (*Type eN* and *Type eB*; names derived from their characteristic *N*arrow and *B*road action potentials, preceded by “e” to indicate electrophysiological categorization, Fig. S5). *Type eN* MRR^vGluT2→LHb^ neurons were found to have larger cellular capacitance and lower input resistance. They often showed bursting behavior and they emitted faster and larger action potentials (Fig. 5B–D, Fig. S7A-C, Table S3, S9, S10). After 3 dimensional reconstructions, we also analyzed the localization (Fig. S7D) and the morphological features of MRR^vGluT2→LHb^ neurons (Fig. 5E, F, Fig. S9A). In line with the observed difference in input resistance and cellular capacitance, both groups had relatively large dendritic arborizations with subtype-dependent dendritic length, but with similar spine density (Fig. S9B–E, Table S3).

### MRR^vGluT2→LHb^ neurons comprise distinct subpopulations based on their genetic properties

To identify neurotransmitter systems that regulate MRR^vGluT2→LHb^ neurons and to explore potential drug targets for future therapies, such as those for depression, we analyzed the mRNA content of labeled MRR^vGluT2→LHb^ neurons by collecting their nuclei after the in vitro experiments described above (Fig. 5A, Fig. S5, S6, and S8). We found that besides common glutamatergic and GABAergic receptor families, MRR^vGluT2→LHb^ neurons typically expressed opioid-related nociceptin receptor (*Oprl1)*, cannabinoid receptor *(Cnr1)*, glycine receptor *(Glrb)*, histamine receptor *(Hrh1)* and acetylcholine receptor (*Chrna4*) as well (Fig. 5D, Fig. S6 and S8) suggesting that the activity of MRR^vGluT2→LHb^ neurons is regulated by behaviorally relevant pharmacological pathways. The mRNA data also showed that these neurons expressed vGluT2, but not vesicular GABA transporter (vGAT), confirming their purely glutamatergic nature^31^ (Fig. S8).

Despite shared features, hierarchical cluster analysis of mRNA expression revealed three distinct subtypes - *Type gG, Type gC*, and *Type gH* - named after one of their most unique gene markers and preceded by “g” to indicate genetic categorization (Fig. 5D, Fig. S5B, Fig. S6; see Methods). *Type gH* neurons were parvalbumin (PV)-negative and were all identified electrophysiologically as *Type eB* neurons (Fig. S8K, Table S3). In contrast, both *Type gG* and *Type gC* neurons expressed significantly higher levels of PV (Fig. S8K).

*Type gG* and *Type gC* neurons (which together largely corresponded to the *Type eN* electrophysiological phenotype) were approximately three times more prevalent (28/38, ~74%) than *Type gH* neurons. Among the 37 MRR^vGluT2→LHb^ neurons analyzed both electrophysiologically and genetically, 34 neurons exhibited consistent clustering across electrophysiological and transcriptional profiles, supporting the robustness of this classification. The remaining three neurons displayed a *Type eB* electrophysiological profile but a *Type gG* genetic signature (Fig. 5D, Fig.S6). All *Type gH* neurons were *Type eB* and - with the above-mentioned 3 exceptions - all *Type gC* and *Type gG* neurons were *Type eN* neurons (Fig. 5D, Fig. S6).

### PV is selectively expressed in MRR^vGluT2^ neurons

**Fig. 6:**
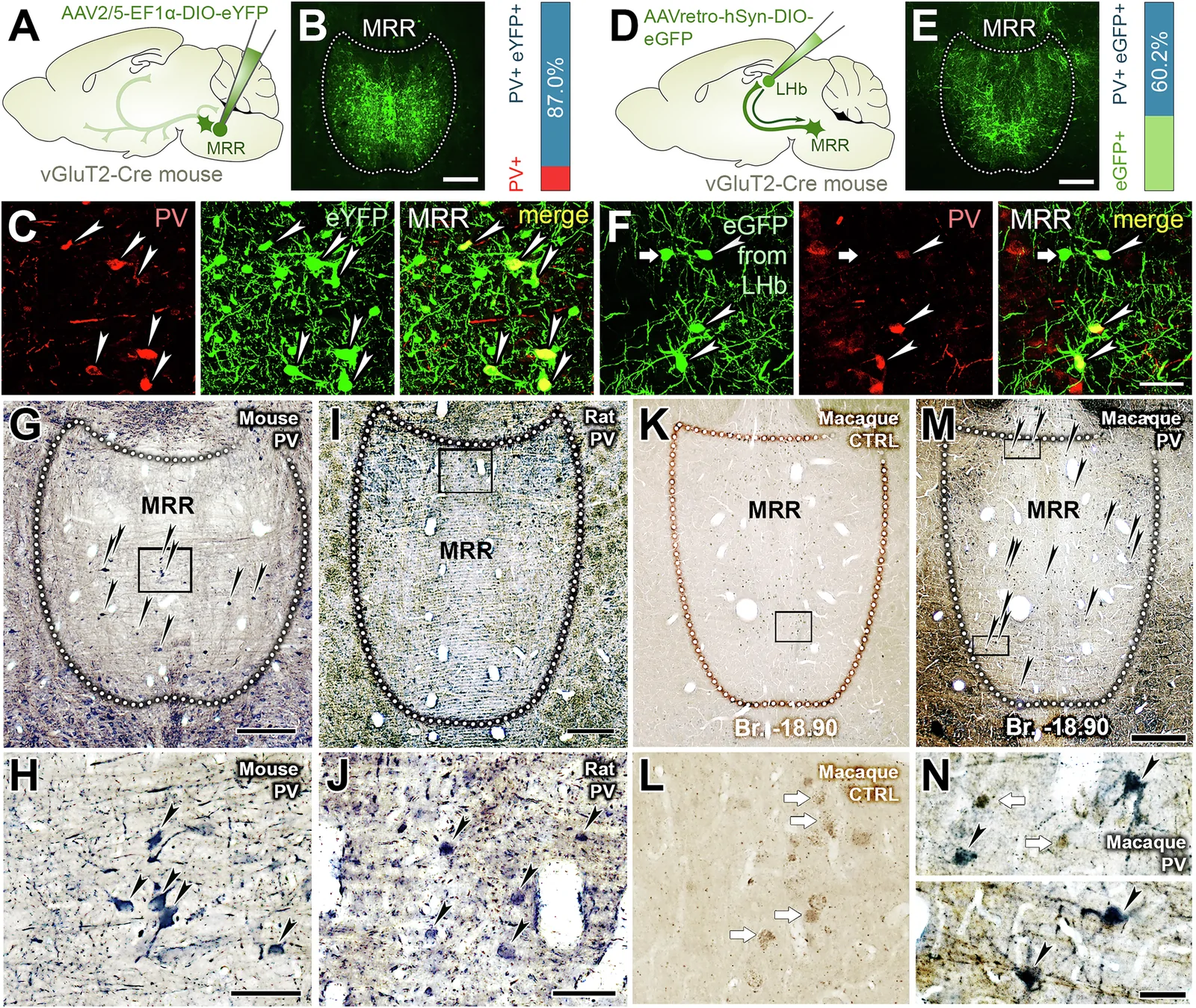
MRR^PV^ neurons identified in mouse, rat and monkey pons. **A** Cre-dependent, eYFP-encoding AAV was injected into the MRR of two vGluT2-Cre mice. **B** Injection site labeling in the MRR. Scale bar, 200 μm. **C** Confocal laser-scanning images and graph show that the vast majority (87%, 67/77 neurons from 2 mice) of PV immunostained neurons (red) were positive for eYFP (green, white arrows) in the MRR. Scale bar for C and F, 50 μm. **D** Cre-dependent, eGFP-encoding, retrograde AAV was injected into the LHb of three vGluT2-Cre mice bilaterally. **E** Images show retrogradely labeled MRR^vGluT2→LHb^ neurons (green). Scale bar, 200 μm. **F** Images and a graph show that 60.2% (62/103 neurons from 3 mice) of the retrogradely-labeled GFP-containing, MRR^vGluT2→LHb^ neurons (green) were immunopositive for PV (red). Scale bar for C and F, 50 μm. **G** Brightfield microscopic image shows PV-positive MRR neurons (black DAB-Ni staining) in the mouse pons (confirmed in 2 mice). Scale bar, 200 μm. **H** A higher magnification image from panel G shows a group of MRR^PV^ neurons (arrows) in the mouse. Scale bar, 50 μm. **I** Image shows PV-positive MRR neurons (black DAB-Ni staining) in rat pons (confirmed in 4 rats). Scale bar, 200 μm. **J** A higher magnification image from panel I shows a group of MRR^PV^ neurons (arrows) in the rat. Scale bar, 50 μm. **K** Representative image shows control staining with the lack of labeling without PV primary antibody in the MRR of the rhesus macaque (at Bregma −18.90 mm). **L** Inset from panel K showing control staining without PV antibody with only faint brown lipofuscin (white arrows) in the MRR. **M**,** N** PV staining (black DAB-Ni precipitate) reveals MRR^PV^ neurons (black arrows) in the monkey [brown lipofuscin (white arrows) staining is also present in PV-stained section]. Scale bars, 500 μm for **K** and M and 50 μm for **L** and **N**.

Genetic data showed that PV expression is prevalent among MRR^vGluT2→LHb^ neurons, which, therefore, can potentially be used as a marker for MRR^vGluT2^ neurons around the MRR. We labeled MRR^vGluT2^ neurons using an injection of Cre-dependent, eYFP-encoding AAV into the MRR of vGluT2-Cre mice (Fig. 6A, B) and we labeled PV cells using immunohistochemistry. We found that the vast majority of MRR^PV^ neurons [at least 87% (67/77) neurons from 2 mice] were also eYFP (vGluT2) positive (Fig. 6C).

We also confirmed that MRR^PV^ neurons densely project to the LHb with vGluT2-positive terminals (see Fig. S10A–F). Additionally, we directly investigated the PV expression within the population of MRR^vGluT2→LHb^ neurons. We labelled MRR^vGluT2→LHb^ neurons with a retrogradely spreading, Cre-dependent, eGFP-encoding AAV injected into the LHb of vGluT2-Cre mice (Fig. 6D, E), and we simultaneously labeled PV in MRR neurons using immunohistochemistry. Indeed, the majority of MRR^vGluT2→LHb^ neurons [at least 60% (62/103) neurons from 3 mice] were also PV-positive by immunohistochemistry (Fig. 6F).

Our genetic data suggested that about 25% of the MRR^vGluT2→LHb^ neurons are *Type gH*, which typically lack PV. To investigate whether their projection pattern in the LHb is different from *Type gG and Type gC* neurons, we labeled PV-positive (*Type gG* and *Type gC*) and PV-negative (*Type gH*) MRR^vGluT2→LHb^ neurons using mutually exclusive AAV constructs, with green and red fluorescent protein expressions, respectively (Fig. S10H). We found that they target the LHb with similar projection pattern (Fig. S10I), with vGluT2 immunopositive terminals (Fig. S10J). These results suggest that despite the diversity of their genetic and electrophysiological properties, all MRR^vGluT2^ types provide similar excitatory input to the LHb.

### MRR^vGluT2/PV^ neurons are present in mice, rats and primates

To confirm that PV-positive MRR^vGluT2^ neurons (MRR^vGluT2/PV^) are evolutionarily conserved, we demonstrated their presence in rat and primate brains as well. First, we injected Cre-independent, mCherry-encoding AAV into the MRR of Wistar rats (Figure S11A, B). Their axonal distribution in the LHb (Fig.S11C) closely resembled MRR^vGluT2→LHb^ axonal distribution in mice and their axon terminals were also immunopositive for vGluT2 but not for vGAT in the LHb (Fig. S11D).

Because most MRR^vGluT2→LHb^ neurons are PV-positive, we localized their cell bodies in rats and primate monkey brains, using precipitation-based PV immunostaining. Using light microscopy, we identified these MRR^PV^ neurons in two mice and four rats (Fig. 6G–J). In addition, we identified MRR^PV^ neurons in the brain of the rhesus monkey (*Macaca mulatta*) (Fig. 6K–N), indicating that these neurons are conserved across mammalian species.

### MRR^vGluT2→LHb^ neurons are directly targeted by the mPFC^→MRR^ neurons

**Fig. 7:**
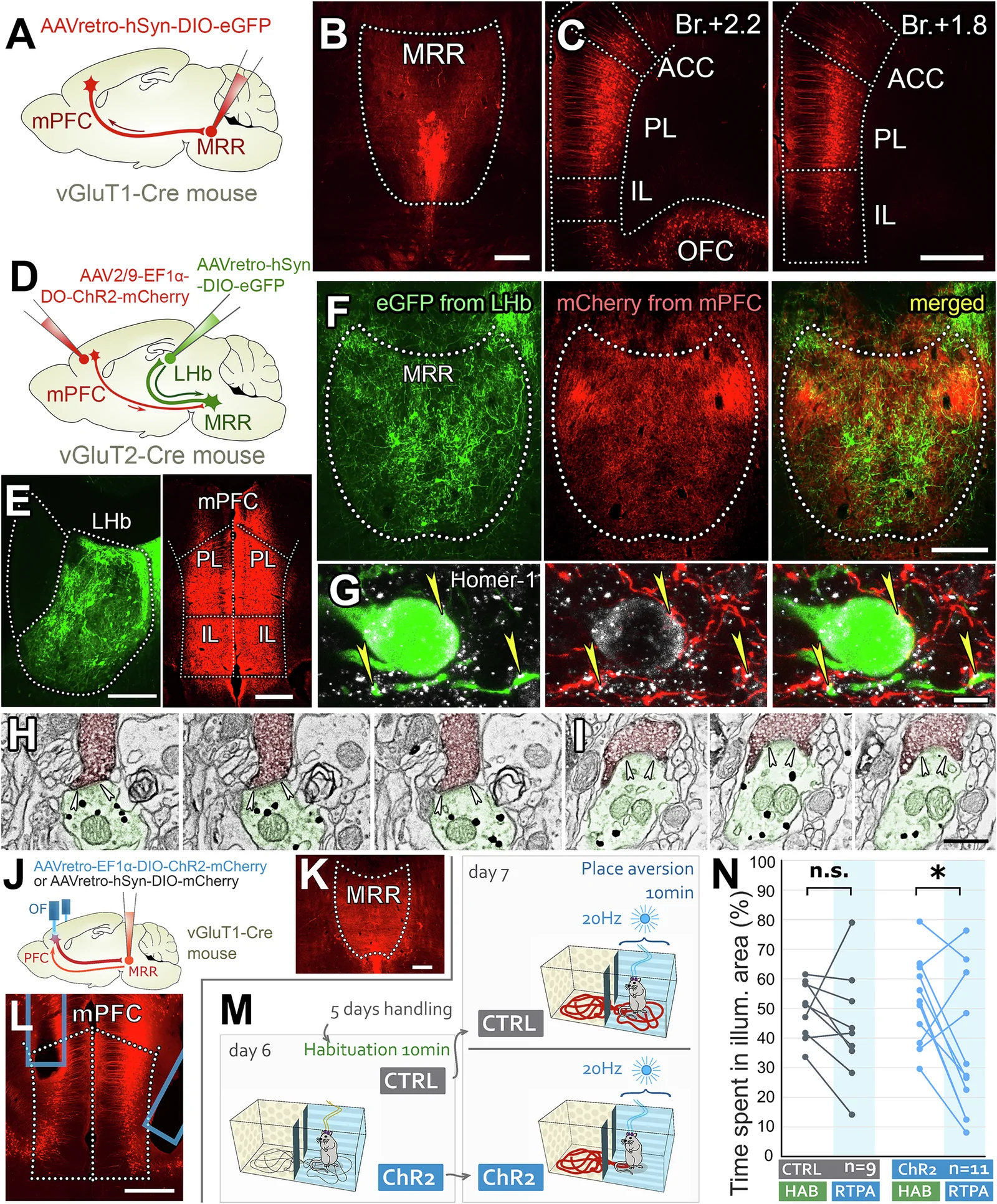
mPFC neurons target MRR^vGluT2→LHb^ neurons and induce negative experience. **A** eGFP-encoding, Cre-dependent, retrograde AAV, injected into the MRR, labeling mPFC^→MRR^ neurons in two vGluT1-Cre mice. **B** eGFP expression (red) in the MRR. Scale bar, 200 μm. **C** Representative fluorescent images from two Bregma coordinates (Br. +2.2 mm and Br. +1.8 mm) show mPFC^→MRR^ neurons (red) in the anterior cingulate cortex (ACC), the prelimbic cortex (PL), the infralimbic cortex (IL) and the orbitofrontal cortex (OFC). Scale bar, 500 μm. **D**,** E** Retrograde, bilateral labeling of MRR^vGluT2→LHb^ neurons (green eGFP-expression) from the LHb was combined with the anterograde, bilateral viral labeling of mPFC^→MRR^ neurons (red mCherry-expression) in the prelimbic (PL) and the infralimbic (IL) mPFC regions of four vGluT2-Cre mice. Scale bars, 200 μm for the LHb and 500 μm for the mPFC. **F**,** G** mPFC fibers (red) innervate the MRR and establish homer-1 (white) positive, putative synaptic contacts (yellow arrowheads) on eGFP-labeled MRR^vGluT2→LHb^ neurons (green). Scale bars, 200 μm and 5 μm. **H**,** I** Serial electron micrographs show two representative examples of the synaptic contacts (synaptic edges marked with arrowheads) established by mPFC terminals (DAB precipitate, red) on the dendrites of MRR^vGluT2→LHb^ neurons (labeled retrogradely from the LHb, immunogold labeling, green). Scale bar, 500 nm. **J** Cre-dependent, retrograde AAV was injected into the MRR of vGluT1-Cre mice to label mPFC^→MRR^ neurons. Control (CTRL) mice received mCherry-encoding AAV, ChR2 mice received ChR2- and mCherry-encoding AAV. Optic fibers were implanted over the mPFC bilaterally. **K**,** L** mCherry expression (red) shows the injection site in the MRR and mPFC^→MRR^ neurons in the mPFC. Optic fibers (blue). Scale bars, 200 μm for the MRR and 500 μm for the mPFC. **M** Place aversion test: After handling, on day 6: mice were habituated (HAB) to the two-chamber box. Day 7: mice received 20 Hz light exposure via the optic fibers in only one of the chambers to test their place aversion. **N** Unlike CTRL mice, ChR2 mice significantly avoided the chamber with mPFC^→MRR^ activation (n_CTRL_=9, n_ChR2_ = 11). For statistical details, see Table S1. For reconstructed optic fiber localization see Figure S16. Source data are provided as a Source Data file.

Our behavioral, electrophysiological and calcium imaging data demonstrated that MRR^vGluT2^ neurons have a crucial role in contextual and cued fear memory recall. Contextual and cued fear memory centers, the hippocampus and the basolateral amygdala target the mPFC^11,55–57^. We hypothesized that the mPFC recalls the negative affective valence of aversive memories by activating the MRR^vGluT2^ neurons directly. First, we confirmed that the mPFC targets the MRR. We injected Cre-dependent, retrogradely spreading, eGFP-encoding AAV into the MRR of vGluT1-Cre mice (Fig. 7A, B) to label its cortical inputs. We found a large population of MRR targeting neurons in the anterior cingulate cortex (ACC), the prelimbic cortex (PL), the infralimbic cortex (IL) and the orbitofrontal cortex (OFC) (Fig. 7C, Fig.S12F–H).

Then, to show that the mPFC directly targets MRR^vGluT2→LHb^ neurons, we infected the mPFC neurons directly with mCherry-encoding AAV and we injected a Cre-dependent, retrogradely spreading, eGFP-encoding AAV into the LHb to label MRR^vGluT2→LHb^ neurons in vGluT2-Cre mice (Fig. 7D, E). Fluorescent images revealed that the mPFC densely and specifically innervate the MRR and their fibers arborize around the MRR^vGluT2→LHb^ neurons (Fig. 7F, Fig. S12A–E). Immunostaining of homer-1 (an excitatory postsynaptic scaffolding protein) combined with confocal laser scanning imaging revealed putative, excitatory synaptic contacts from the mPFC^→MRR^ neurons on MRR^vGluT2→LHb^ neurons (Fig. 7G). At least 83% (25/30) of MRR^vGluT2→LHb^ neurons received, on average 6-7 putative synapses from mPFC (161 homer-1-positive putative synapses in four mice). Synapses from mPFC to MRR neurons were also confirmed using electron microscopy (16 identified synapses in 2 mice, Fig. 7H–I, Fig. S14 A–D).

Additionally, using anterograde trans-synaptic viral tracing, we confirmed that the MRR^vGluT2→LHb^ neurons that are directly targeted by synaptic connections from mPFC neurons, send axonal collaterals to the LHb (Fig. S13).

### mPFC^→MRR^ neurons are sufficient to induce negative experience

Having shown that MRR^vGluT2^ neuronal activity can be associated with an auditory cue (Fig. S1F–I), we next investigated whether the upstream mPFC^→MRR^ input to these neurons is sufficient to induce an ongoing negative affective state. Therefore, we examined how optogenetic activation of mPFC^→MRR^ neurons influences mouse behavior. We injected retrogradely spreading, Cre-dependent, excitatory ChR2-encoding AAV (ChR2 mice) or control AAV (CTRL mice) into the MRR of vGluT1-Cre mice (Fig. 7J, K). We then implanted optic fibers into the mPFC bilaterally (Fig. 7L). After handling, on day 6, mice were placed in a double-chamber box to test their baseline chamber-preference. On day 7, mice were placed back into the double-chamber box, where the mPFC was illuminated when mice entered one of the test chambers (Fig. 7M). Unlike CTRL mice, ChR2 mice exhibited significant real-time place aversion to the chamber paired with optic fiber illumination, relative to their baseline preference (Fig. 7N, Table S1). These data suggest that the activation of mPFC^→MRR^ neurons is sufficient to induce a negative experience. Thus, these two assays (Fig. S1F–I and Fig. 7J–N) addressed closely related but distinct questions: whether the pathway can generate a memory of a negative experience, and whether activation of its upstream cortical input can directly evoke negative valence.

### mPFC^→MRR^ neurons represent negative experiences in a scalable manner

**Fig. 8:**
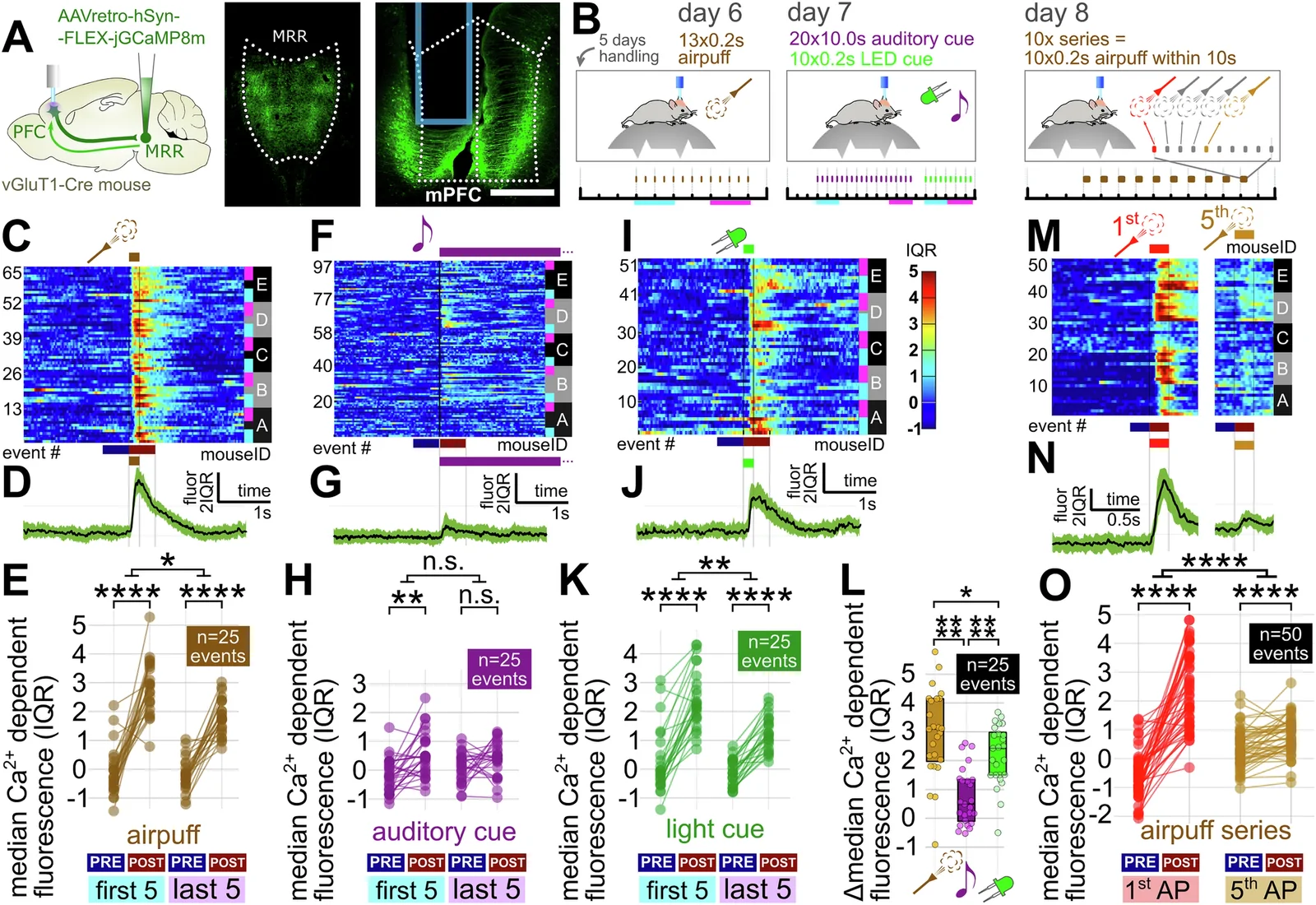
Activity of mPFC^→MRR^ neurons scales with the aversiveness of negative experiences. **A** Cre-dependent, GCaMP8m-encoding, retrograde AAV was injected into the MRR of vGluT1-Cre mice to label mPFC^→MRR^ neurons. An optic fiber was implanted over the left mPFC. Fluorescent images show the injection site in the MRR (green). Retrogradely labeled mPFC^→MRR^ neurons (green), and the location of the optic fiber (blue) in the mPFC. Scale bar, 500 μm for both images. **B** Head-fixed fiber photometry protocol and timeline. Day 6: thirteen 0.2-second-long airpuffs. Day 7: twenty 10-seconds-long auditory tones and ten 0.2-second-long LED light flashes. Day 8: ten airpuff series, each series had ten 0.2-second-long airpuffs within 10 seconds. Heatmap plot shows changes in mPFC^→MRR^ activity (detrended, normalized, Ca^2+^-dependent signals) aligned to (**C**) airpuffs (**F**) tone cues and (**I**) LED light flashes, respectively (n_events_=65, 97, 51 respectively, 5 mice). Fiberphotometry units are normalized to the interquartile range (IQR, see methods).**D**, **G** and **J** Perievent plots show medians and interquartile ranges of mPFC^→MRR^ activity aligned to each stimulus respectively. **E**,** H** and** K** Plots show a comparison of mPFC^→MRR^ neuronal activity before (0.5 s) and after (0.5 s) the start of the stimuli. Data are calculated for the first and last 5 stimuli. Differences in the amount of fluorescence changes are also compared between the first 5 and the last 5 stimuli (n_events_=25; 5 mice). **L** Graph shows that mPFC^→MRR^ activity follows presumed stimulus aversiveness (n_events_=25; 5 mice), inferred from putative aversive salience. **M** Heatmap plots show quick adaptation of mPFC^→MRR^ neurons to the same airpuffs already within the first 5 seconds of the series (n_events_=50; 5 mice) **N** Perievent plots show medians and interquartile ranges of mPFC^→MRR^ activity aligned to the first (left) and the fifth (right) airpuffs in each airpuff series. **O** mPFC^→MRR^ neuronal activation (0.2 s before, 0.2 s after airpuff) is significantly higher in the first (left) than in the fifth (right) airpuffs in each series, these were delivered within 5 seconds (n_events_=50; 5 mice). Graphs show medians and interquartile ranges. For statistical details, see Table S2. For reconstructed optic fiber localization see Fig. S16. Source data are provided as a Source Data file.

To investigate the specific contributions of mPFC^→MRR^ neurons to the processing of fear memory and to assess whether their activity scales proportionally with threat intensity, we performed fiber photometry experiments. To selectively record mPFC^→MRR^ neuronal activity, we injected Cre-dependent, GCaMP-encoding, retrograde AAV into the MRR of vGluT1-Cre mice. We then implanted optic fibers over the left mPFC (Fig. 8A). After handling, mice were head-fixed, and we recorded mPFC^→MRR^ neuronal responses (and control isosbestic fluorescence signal) to different stimuli. On day 6, we presented 13 aversive airpuffs. On day 7, we presented 20 auditory cues as neutral stimuli and later, 10 mildly aversive light flashes (Fig. 8B). All stimuli caused significant activation of mPFC^→MRR^ neurons initially (Fig. 8C–K, Table S2). However, we found that the aversive airpuffs induced the strongest activation of mPFC^→MRR^ neurons (Fig. 8L, Table S2). Light flashes induced significantly weaker activation, whereas neutral auditory cues caused only sparse activation that was significantly weaker than the responses to light activation (Fig. 8L, Table S2).

Adaptation to adverse events is vitally important and can occur at different time scales. We found that the mPFC^→MRR^ neurons show significantly different activation for early (the first 5) and late (the last 5) presentation of the same airpuffs (Fig. 8E) and light flashes (Fig. 8H), whereas the last 5 auditory cues could not activate mPFC^→MRR^ neurons anymore (Fig. 8K, Table S2). This demonstrates that signal adaptation can unfold on the timescale of several minutes.

In addition, we investigated whether adaptation could occur also on the timescale of a few seconds. We analyzed the activity of mPFC^→MRR^ neurons, after five, 0.2-second-long airpuffs were presented within 5 s (day 8, Fig. 8B). We found that the first airpuffs in each series evoked significantly higher mPFC^→MRR^ neuronal activation than the 5^th^ airpuffs (Fig. 8M–O, Table S2). None of these differences were present in the control isosbestic signals (Fig. S15, Table S2). These data demonstrate that the activity of mPFC^→MRR^ neurons is tightly regulated by stimulus intensity and can adaptively modulate on a timescale of both seconds and minutes.

### mPFC^→MRR^ neurons are necessary for fear memory recall

**Fig. 9:**
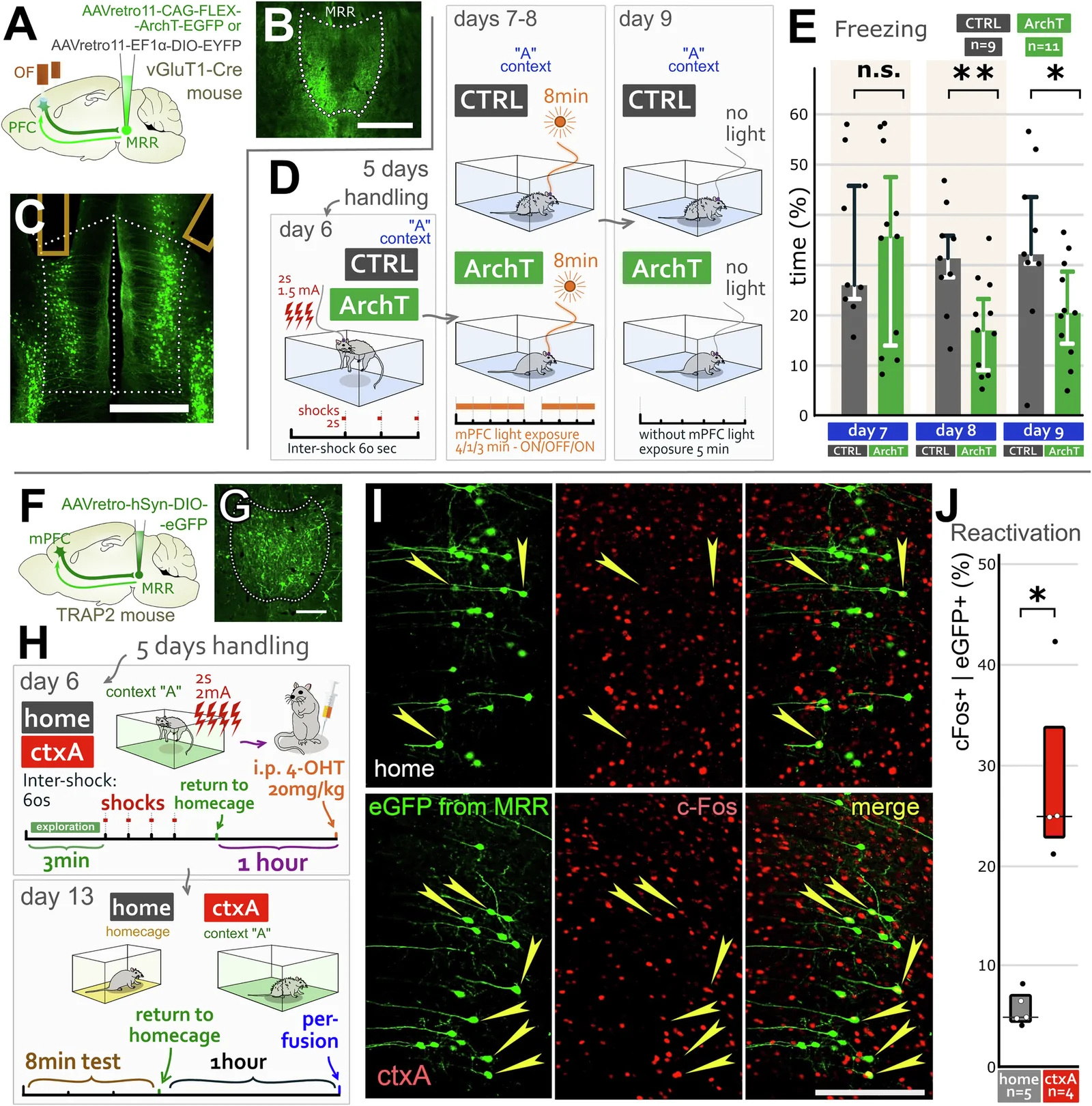
mPFC^→MRR^ neurons are necessary for fear recall and reactivate during fear recall. **A**,** B** Cre-dependent, retrogradely spreading AAV was injected into the MRR in vGluT1-Cre mice. CTRL mice received only EYFP-encoding AAV. ArchT mice received ArchT-EGFP-encoding AAV. Scale bar, 500 μm **C** Two optic fibers were implanted bilaterally over the mPFC to deliver light to mPFC^→MRR^ neurons. Image shows mPFC^→MRR^ neurons (green) and the position of the optic fibers (orange). Scale bar, 500 μm. **D** Contextual fear conditioning test: After handling, on day 6, mice received 3 foot-shocks (2 s, 1.5 mA) in context “A”. Days 7-8: mice were placed back into context “A” for 8 minutes to test their contextual fear memories, while they received 7 min light exposure via the optic fiber (8 mW). Day 9: fear recall test in context “A” for 5 min without light exposure. **E** ArchT mice showed significantly less fear (freezing) compared to CTRL mice on day 8, and also on day 9 without light exposure (n_CTRL_=9, n_ArchT_ = 11). **F** Cre-dependent, eGFP-encoding retrograde AAV was injected into TRAP2 mice. **G** eGFP expression (green) in the MRR. Scale bar, 200 μm. **H** Contextual fear recall test. Mice received four foot-shocks (2 mA, 2 s) in context “A”. One hour later, mice received 4-hydroxytamoxifen (4-OHT, 20 mg/kg) injection intraperitoneally. A week later ctxA-mice were placed back into context “A”, whereas home-mice were left in their homecage. One hour later the mice were perfused. **I** Representative fluorescent images revealed significantly higher reactivation [expression of c-Fos protein (red)] in mPFC^→MRR^ neurons (green) in a ctxA-mice (bottom row) compared to home-mice (top row). Scale bar, 200 μm. **J** Significantly higher proportion of mPFC^→MRR^ neurons expressed c-Fos in ctxA-mice (n_home_=5, n_ctxA_ = 4). Graphs show medians and interquartile ranges. For statistical details, see Table S1. For reconstructed optic fiber localization see Fig. S16. Source data are provided as a Source Data file.

To evaluate whether mPFC→MRR pathway is involved in fear memory recall, we inhibited the mPFC^→MRR^ somata during contextual fear memory recall. We injected retrogradely spreading, Cre-dependent, inhibitory ArchT-encoding AAV (ArchT mice) or control AAV (CTRL mice) into the MRR of vGluT1-Cre mice (Fig. 9A, B). We then implanted an optic fiber above the somata in the mPFC bilaterally (Fig. 9C). After handling, on day 6, all mice received foot-shocks in context “A”. Both groups showed normal acute fear behavior during conditioning (freezing behavior, Figure S4M-Q). On days 7-8, we placed mice back into context “A” and we exposed them to light via the optic fibers (Fig. 9D). We found that, compared to CTRL mice, ArchT mice showed a significantly lower contextual fear response on day 8 during light exposure (Fig. 9E, Table S1). Finally, on day 9, mice were placed back into context “A” without optic fiber light exposure, and the significantly reduced freezing in ArchT-expressing mice persisted (Fig. 9E, Table S1). This shows that mPFC^→MRR^ neurons contribute to contextual fear memory recall and prevent premature fear memory extinction.

### Fear encoding mPFC^→MRR^ neurons are reactivated during fear memory recall

To independently verify that the mPFC controls fear memory via MRR^vGluT2^ neurons, we genetically tagged fear-encoding mPFC^→MRR^ neurons and labeled those reactivated during fear recall. First, we injected a retrogradely spreading, Cre-dependent, eGFP-encoding AAV into the MRR of Fos2a/iCre-ERT2 (TRAP2) mice (Fig. 9F, G). This virus infected (but it did not yet label) mPFC^→MRR^ neurons. After handling, all mice received aversive foot-shocks in context “A” and received 4-hydroxytamoxifen (4-OHT) intraperitoneally (Fig. 9H). 4-OHT allows eGFP-expression in active, cFos-expressing, AAV-infected neurons in TRAP2 mice. One week later ctxA-mice were placed back into context “A” for 8 minutes contextual fear memory recall. The control group remained in their homecage (home-mice). One hour later all the mice were prepared for immunohistochemistry to localize cFos-expression during fear recall in reactivated mPFC^→MRR^ neurons (Fig. 9H). eGFP-labeling in a large population of MRR neurons (Fig. 9G) and in mPFC^→MRR^ neurons (Fig. 9I, green neurons) revealed that both the mPFC^→MRR^ neurons and the MRR neurons are directly activated during contextual fear encoding. Furthermore, direct c-Fos immunostaining showed that the fear encoding mPFC^→MRR^ neurons were significantly (about 5 times more) reactivated in ctxA-mice compared to home-mice (Fig. 9I–J, Table S1). This demonstrated that fear-encoding mPFC^→MRR^ neurons were reactivated during fear recall one week after fear conditioning, suggesting that they directly reactivate MRR^vGluT2^ neurons during contextual fear memory recall.

### mPFC^→MRR^ neurons receive converging inputs from the hippocampus and amygdala

**Fig. 10:**
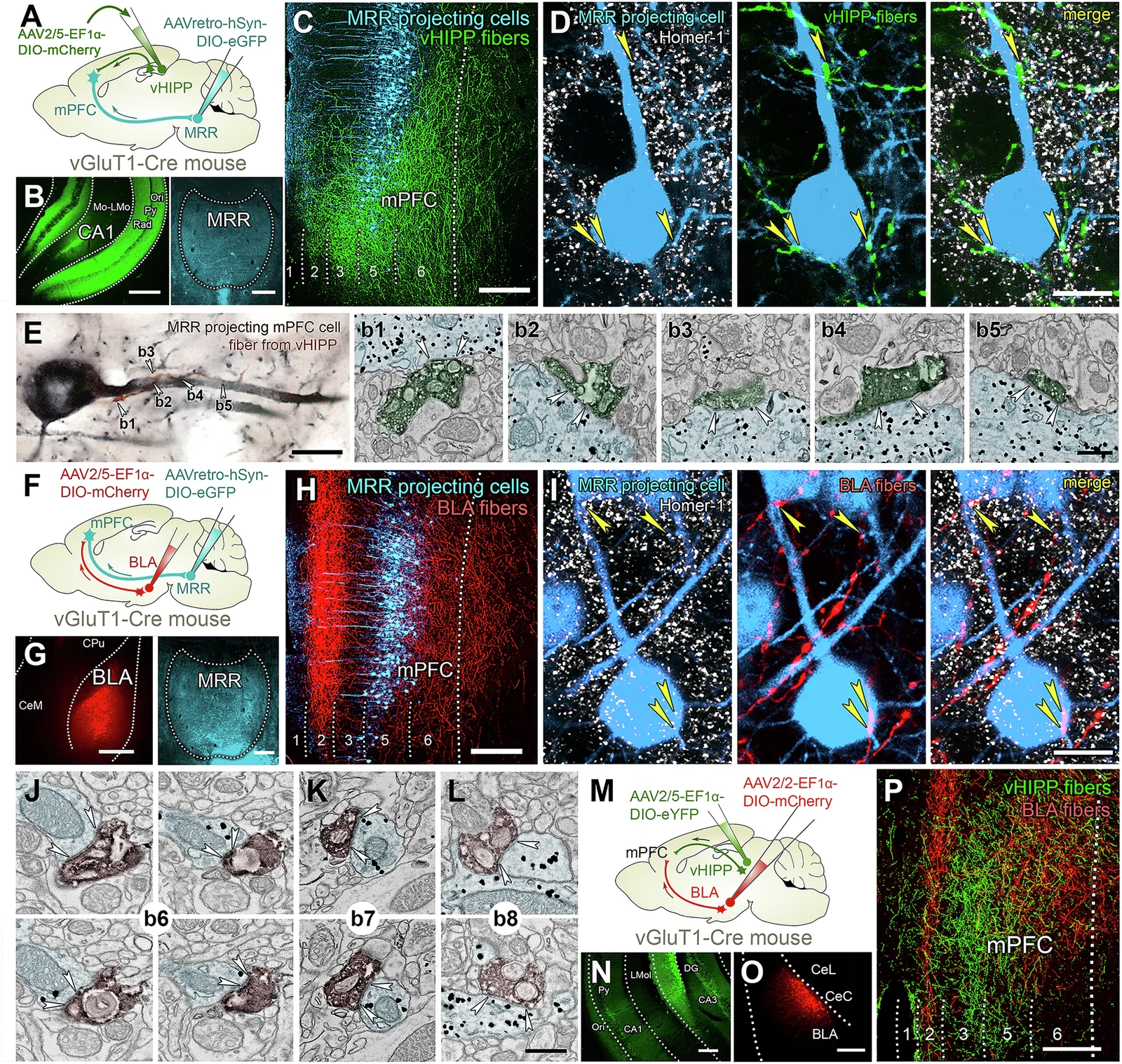
mPFC^→MRR^ neurons are targeted by the vHIPP and the BLA. **A** Retrograde AAV-labeling of mPFC^→MRR^ neurons (cyan) and bilateral, anterograde AAV-labeling of vHIPP neurons (green) in two vGluT1-Cre mice. **B** Images show mCherry expression (here showed in green) in the vHIPP and eGFP expression (cyan) in the MRR. Scale bars, 200 μm. **C** vHIPP fibers (green) target the mPFC^→MRR^ neurons (cyan, retrogradely labeled from the MRR). Scale bar, 200 μm. **D** Confocal laser scanning images show that vHIPP fibers (green) establish homer-1-positive (white) putative synaptic contacts (yellow arrowheads) with mPFC^→MRR^ neurons (cyan). Scale bar, 10 μm. **E** Brightfield image shows that a vHIPP axon (brown DAB precipitate) wraps tightly around the dendrite of a mPFC^→MRR^ neuron (retrogradely labeled from the MRR, black immunogold labeling), forming numerous synapses. Scale bar, 10 μm. Electron micrographs show 5 synapses (**b1-5**, synaptic edges are marked with arrowheads) established by vHIPP boutons (DAB precipitate, green) on the mPFC^→MRR^ neuron (immunogold labeling, cyan). Scale bar, 500 nm. **F** Retrograde AAV-labeling of mPFC^→MRR^ neurons (cyan) and bilateral, anterograde AAV-labeling of BLA neurons (red) in two vGluT1-Cre mice. **G** Images show mCherry expression (red) in the BLA and eGFP expression (cyan) in the MRR. Scale bars, 200 μm. **H** BLA fibers (red) target the mPFC^→MRR^ neurons (cyan, retrogradely labeled from the MRR). Scale bar, 200 μm. **I** Confocal laser scanning images show that BLA fibers (red) establish homer-1-positive (white) putative synaptic contacts (yellow arrowheads) with mPFC^→MRR^ neurons (cyan). Scale bar, 10 μm. **J–L** Electron micrographs show 3 synapses (**b6, b7, b8**, synaptic edges are marked with arrowheads) established by BLA boutons (DAB precipitate, red) on the dendrites of the mPFC^→MRR^ neurons (immunogold labeling, cyan). Scale bar, 500 nm. **M** Combination of the anterograde AAV labeling of vHIPP neuronal fibers (green) and BLA fibers (red) in two vGluT1-Cre mice. **N, O** Images show injection sites for eYFP-labeled (green) vHIPP and mCherry-labeled (red) BLA areas. Scale bars, 200 μm. **P** Fluorescent imaging reveals the slightly distinct localization patterns of HIPP and BLA fibers targeting the region containing mPFC^→MRR^ neurons. Scale bar, 200 µm.

The hippocampus (HIPP) and the basolateral amygdala (BLA) are required for contextual and cued fear memory recall^8,19^. We hypothesized that HIPP and BLA neurons influence MRR^vGluT2^ neurons via the mPFC→MRR pathway, thereby facilitating the recall of the aversive components of fear memories. Therefore, we investigated their connections using retrograde and anterograde AAV tracing combined with fluorescent and electron microscopy. First, we injected Cre-dependent, mCherry-encoding AAV into the ventral HIPP (vHIPP, Fig. 10A) or the BLA (Fig. 10F) of vGluT1-Cre mice to label their fibers in the mPFC. Then we injected a Cre-dependent, retrograde, eGFP-encoding AAV into the MRR of the same mice (Fig. 10A, B, Fig. 10F, G) labeling mPFC^→MRR^ neurons. Fluorescent images revealed that both vHIPP (Fig. 10C, D) and BLA fibers (Fig. 10H, I) arborize around mPFC^→MRR^ neurons (Fig. 10C, Fig. 10H). Immunostaining of homer-1 revealed that at least 80% (53/66 from two mice) of the mPFC^→MRR^ pyramidal cells receive at least one synaptic contact (on average 4.64) from vHIPP neurons (Fig. 10D). Further measurements revealed that at least 60% (27/45 from two mice) of mPFC^→MRR^ pyramidal cells receive at least one synaptic contact (on average 4.07) from BLA neurons (Fig. 10I). These synaptic connections were also confirmed using electron microscopy (Fig. 10E and J–L). Electron microscopic reconstruction of synapses revealed that vHIPP inputs to mPFC^→MRR^ neurons targeted 1 spine, 3 somata, and 12 dendritic shafts (16 synapses, 2 mice; Fig. 10E, Fig. S14E–H), while BLA inputs targeted 4 spines, 1 soma, and 14 dendritic shafts (19 synapses; 2 mice; Fig. 10J–L, Fig. S14I–L) in our samples. Further tracing experiments revealed a distinct localization patterns of vHIPP and BLA fibers targeting the region containing mPFC^→MRR^ neurons (Fig. 10M–P). This aligns with previous findings regarding BLA fibers^55^. These data demonstrated that the fibers from the contextual and cued fear memory recall-related HIPP and BLA establish synaptic contacts on the mPFC^→MRR^ projection neurons completing the described fear recall circuit.

## Discussion

In the canonical tripartite fear memory circuit, the prelimbic mPFC was found to be involved in fear memory recall, the infralimbic mPFC in fear extinction, whereas the BLA and the HIPP were found to be necessary for both^8,19,58^. Although, we previously demonstrated that MRR^vGluT2^ neurons play a role in the acquisition of negative experiences^31^, their involvement in recalling those memories was unknown. Learning and recall are computationally and mechanistically distinct operations: while aversive learning requires the integration of ongoing nociceptive/aversive and contextual/cue information to form or update the memory circuits, recall reactivates an already stored representation and re‑evaluate its affective value in the absence of the original aversive input^59^. However, whether the MRR is required to re-engage the aversive affective component associated with the recalled memory has not been investigated.

Here, we demonstrated that MRR^vGluT2^ neurons have an important role in fear memory recall, because their suppression induced both impaired fear recall and enhanced fear extinction. This has not been previously demonstrated in its upstream tripartite fear circuit. Our finding that MRR^vGluT2^ neurons are necessary for both contextual and cued fear memory recall and for maintaining a fear response during fear extinction clearly suggests that MRR^vGluT2^ neurons are necessary for re-engaging the negative emotional component previously linked to the experience, thereby reinstating the affective state associated with the recalled memory. Because recall intensity must be flexible, for instance during fear extinction, neuronal responses must also be scalable, like those observed in the amygdala^8^. Here, using both optogenetic and electrophysiological methods, we demonstrated that the activity of MRR^vGluT2^ neurons scale with the aversiveness of the memory (i.e. with the degree to which a stimulus, event, or experience is unpleasant or negatively valued), suggesting that it can help appropriately update related fear memory circuits.

Neural circuits of fear recall, anxiety and depression are thought to exhibit great overlap^6,8^. PTSD is thought to be a memory disorder, whereas some of its treatments aim to modify trauma recall^60^. The LHb, and specifically its hyper-excitability, are widely accepted to have a central role in major depression both in humans and in rodents^47,48,61^ with ketamine offering a remedy for treatment-resistant major depression^48,62^ by inducing plasticity^48,63,64^. The LHb plays a key role in aversive information processing and is controlled by MRR^vGluT2^ neurons^31^. Here we demonstrated that this connection is essential for controlling LHb-activity during fear memory recall. We showed that chronic-stress-induced, depression-related anhedonia significantly elevated c-Fos expression in MRR^vGluT2^ neurons even two days after stress induction. Because ketamine is one of the most promising recent therapeutic agents against major depression and it is currently offered as a single clinical treatment, we tested its effect on this pathway. We found that a single ketamine treatment significantly decreased the above mentioned stress-induced c-Fos expression in MRR^vGluT2^ neurons. This suggests that MRR^vGluT2^ neurons may also contribute to the antidepressant effect of ketamine treatment. Moreover, MRR^vGluT2^ neuronal activity may be at least partly responsible for maintaining depressive states. Indeed, we found that the stimulation of MRR^vGluT2^ neurons caused anhedonia and learned helplessness behaviors. Conversely, we found that suppression of MRR^vGluT2^ neuronal activity in stressed animals restored normal reward-seeking behavior, suggesting a neuronal circuit for treating depression. Importantly, these depression-related observations involve the MRR^vGluT2^ neurons that mediate fear recall, reinforcing our proposal that maladaptive reactivation of aversive memories through this circuit may contribute to mood disorders.

Because of their clinical translational potential, we investigated MRR^vGluT2→LHb^ neurons further. Based on their in vitro electrophysiological properties and their gene expression profiles, we found that the MRR^vGluT2^ → LHb pathway is served by a diverse population of neurons. The largest population of MRR^vGluT2→LHb^ neurons are PV-positive, have homogeneous electrophysiological features and can be divided into at least two genetic subtypes (*Type gG* and *Type gC*), whereas the third genetic subtype (*Type gH*) is PV-negative. Our mRNA sequencing data in this study, together with our previous findings^31^, revealed that MRR^vGluT2→LHb^ neurons receive inputs from multiple neurotransmitter systems and aversion-related brain regions. Dysregulation of these inputs may underlie various fear-related pathological states. Our analyses confirmed that PV is a specific marker for MRR^vGluT2^ neurons around the MRR, enabling their identification in both rat and monkey brains, which suggests a strong translational potential.

The mPFC and its forebrain and brainstem connections are known to be involved in fear memory processing^8,15,16,18,65–67^, but the circuit described in the literature is still incomplete. Here we demonstrated that MRR^vGluT2^ neurons are directly targeted by a specific population of mPFC neurons, whose activity is both sufficient to induce and necessary to recall negative experience. We found that the activity of these mPFC^→MRR^ neurons scales with the severity of negative experiences that is, it activates MRR^vGluT2^ neurons stronger in case of a stronger negative experience. We also demonstrated that mPFC^→MRR^ neuronal activity can rapidly adapt to negative experiences and their inhibition during contextual fear memory recall caused impaired fear expression similarly to the direct suppression of MRR^vGluT2^ neuronal activity. Furthermore, our TRAP2 experiments showed that fear encoding mPFC^→MRR^ neurons are specifically reactivated during fear memory recall. Finally, detailed neuronal pathway tracing experiments demonstrated that these MRR targeting mPFC^→MRR^ neurons receive direct synaptic connections from both amygdalar and hippocampal principal neurons.

Our findings show that the mPFC→MRR→LHb pathway has a direct role in recall processes. In contrast to the canonical tripartite fear circuit, which primarily orchestrates the contextual and sensory components of fear, the mPFC→MRR→LHb pathway is specifically required to reinstate and flexibly scale the aversive emotional component of a retrieved memory, as shown by its necessity for both contextual and cued fear recall, its graded activity with memory aversiveness, and its bidirectional control of extinction and depression‑like behaviors. Additionally, impaired fear behavior after inhibition of this pathway is best interpreted as disruption of the negative affective/fear state normally reinstated during recall, rather than as loss of the contextual or auditory memory trace itself.

By dissociating the circuitry that assigns negative valence to recalled memories from the circuitry that initially encodes them, our findings reveal a specific organizing principle for fear networks and provide a defined pontine brainstem pathway through which aversive recall can be selectively targeted in stress‑ and trauma‑related disorders.

In summary, our results suggest that during the recall of negative experience, information flows from the amygdala and the hippocampus to the mPFC, and a population of mPFC neurons directly convey these activities to the MRR^vGluT2^ neurons (Fig. S17). In response, MRR^vGluT2^ neurons activate negative experience-related areas, including the LHb^68^ and the mVTA^31,69^. MRR^vGluT2^ neurons also facilitate memory formation via the septo-hippocampal axis^31,70^ and the nucleus incertus^32,33^, the latter two of which can directly control the hippocampus as well (Fig. S17). When memories are recalled, their underlying neural representation that was active during encoding is reactivated^71^. However, later it becomes plastic and if reinforcement is missing, it eventually becomes less active^8,72^. Our results suggest that MRR^vGluT2^ neurons act as a hub for reassigning negative valence to recalled memories. The canonical tripartite fear memory circuit helps recall the contextual elements of memories, while the MRR^vGluT2^ neurons are directly reactivated to reinforce its association with negative valence. They could modulate the strength of reinforcement during extinction learning and compete with inhibitory memory during fear extinction and have a role in balancing the affective valence of memories. We show that MRR^vGluT2^ neurons are present in primate brain as well, which highlights that they may be an effective therapeutic target for negative affect-related psychiatric disorders in the future.

## Methods

### Ethical considerations

Freely moving fiber photometry experiments were performed in accordance with institutional guidelines at the Friedrich Miescher Institute for Biomedical Research and were approved by the Veterinary Department of the Canton of Basel-Stadt. All other experiments were performed in accordance with the Institutional Ethical Codex and the Hungarian regulations on animal research (Act XXVIII of 1998, Government Decree 40/2013), in line with the EU Directive 2010/63/EU. The Animal-welfare Body of the Institute of Experimental Medicine and the Government Office of Pest County authorized the experiments at project number PE/EA/00063-5/2022 and PE/EA/00513-6/2025.

### Mice and rats

We used vGluT1-iRES-Cre (Stock No.: 023527), vGluT2-iRES-Cre (Stock No.: 028863), PV-Cre (Stock No.: 017320) and Fos2a/iCre-ERT2 (Stock No.: 030323) mice. All Cre-mice were from The Jackson Laboratory. We used C57Bl/6 J mice from Charles River. We used adult (at least 50 days-old) mice from both sexes in our experiments. Mice had access to food and water ad libitum and were housed in a vivarium (3–5 mice/cage) until used in experiments. All mice were maintained on a normal 12 h light-dark cycle, with experiments performed during the light phase of the cycle. Animal holding rooms were climate-controlled, maintaining a temperature range, and humidity range of 21 °C, 35% to 25 °C, 65%.

In case of the in vivo electrophysiology experiments we used 8–16-week-old vGluT2-iRES-Cre mice. Ten of those were used for optogenetical tagging and 8 for lateral habenula-median raphe experiments. Mice were individually housed for at least 14 days before electrophysiological experiments.

In case of the freely moving MRRvGluT2 fiber photometry experiments, we used 8 male vGluT2-iRES-Cre mice. Only heterozygous (Cre/wild type) mice were used for experiments and were backcrossed to a C57BL/6 J background for more than seven generations. Mice were individually housed for at least 14 days before starting behavioral paradigms and had access to a shelter and a running wheel.

We used adult (46–48 days old) male and female Wistar rats also from Charles River.

### Rhesus monkeys (*Macaca mulatta*)

Adult female rhesus monkeys (*Macaca mulatta*) were previously studied by Rovo et al. in 2012^73^. All experimental procedures were performed according to the ethical guidelines of the Institute of Experimental Medicine and Institute of Physiology of the Hungarian Academy of Sciences and approved by the Ethical Committee (Approval 22.1/77/001/2010). Here we investigated the stored brainstem blocks of one of those monkeys.

### Stereotaxic surgeries for viral gene transfer and perfusions in rats

All surgeries were performed in a stereotaxic frame (David Kopf Instruments, CA, USA) under deep anesthesia [achieved using a mixture of ketamine (50 mg/kg; Medicus Partner), xylazine (20 mg/kg; Medicus Partner) and pipolphen (0.2 ml/kg; Egis)]. After a midline incision, a hand drill was used to expose the brain surface above the target site. Ophthalmic ointment (Corneregel or Artelac gel, Bausch&Lomb) was applied to avoid eye drying. Tracer or viral vectors were microinjected through a glass pipette (the diameter of the tip was 20–30 μm) by using Drummond nanoinject III (Drummond, Broomall, PA) into our target brain regions (MRR). For viral labeling, we injected 2 x 60 nl of AAV 2/9-EF1α-DO-hChR2(H134R)-mCherry-WPREpA (Addgene 37082) into the rat MRR. After the nanoinjection, rats received 0.1 ml Bupaq (buprenorphine 0.05 mg/kg) solution subcutaneously to minimize postoperative discomfort and we housed them in their home cage for three weeks until the injected transgenes were expressed. The rats were then anesthetized using a mixture of ketamine (50 mg/kg; Medicus Partner), xylazine (20 mg/kg; Medicus Partner), and pipolphen (0.2 ml/kg; Egis) solution and transcardially perfused with 150 ml of ice-cold saline, followed by 300 ml of 4% paraformaldehyde in 0.1 m phosphate-buffered saline (PBS, pH 7.4). Brains were removed and postfixed.

### Stereotaxic surgeries for viral gene transfer, retrograde tracing, and optic fiber implantations for behavior- and head-fixed fiber photometry experiments in mice

Mice were anesthetized with 2.5% isoflurane in 95% O_2_ (Praxair) vapor followed by an intraperitoneal injection of an anesthetic mixture (containing 8.3 mg/ml ketamine and 1.7 mg/ml xylazine-hydrochloride in 0.9% saline, 10 ml/kg body weight) or we kept mice under deep anesthesia with 1–1.5% isoflurane in 95% O_2_ (Praxair). Then mice were mounted in a small animal stereotaxic frame (David Kopf Instruments, CA, USA) and the skull surface was exposed. Ophthalmic ointment (Corneregel or Artelac gel, Bausch&Lomb) was applied to avoid eye drying. The body temperature of the experimental animal was maintained at 35–37 °C using a feedback-controlled heating pad. A Nanoject III precision microinjector pump (Drummond, Broomall, PA) was used for microinjections. Before the virus injection mice received 0.03 ml 10% Bupaq (buprenorphin) solution subcutaneously to minimize postoperative discomfort. For viral labeling experiments, we injected 30–100 nl of the used virus preparations into the target brain areas (Table S4). The titer of the used viruses were between 4.4–29 × 10^12^ colony forming units/ml. We always (except experiment in Fig. S13) used AAV2/1 viruses in concentration lower than 10^13^ to avoid any anterograde trans-synaptic labeling, and indeed we have never observed such labeling^74^. The coordinates for the injections were defined by a stereotaxic atlas^75^; the null coronal plane of the anteroposterior (AP) axis was defined by the position of Bregma; the null sagittal plane of the mediolateral (ML) axis was defined by the superior sagittal sinus; the null horizontal plane of the dorso-ventral (DV) axis was defined by the positions of Bregma and Lambda points. The injection coordinates can be found in Table S5 (always given in mm at the AP, ML and DV axes, respectively). After the surgeries, mice received 0.3–0.5 ml saline and 0.1 mg/kg meloxicam (Metacam, Boehringer Ingelheim, Germany) intraperitoneally, and were placed into separate cages until further experiments or perfusions. Some illustrations were created using the atlas of Feng et al. (2025)^76^.

### Optic fiber implantations for behavioral and head-fixed fiber photometry experiments

For behavioral optogenetic and head-fixed fiber photometry experiments, virus injections were followed by optic fiber implantation surgeries. These were similar to the virus injections and were carried out 3–6 weeks after virus injections. Optic fibers for behavioral experiments had 200 μm core diameter for mPFC axonal inhibition fear experiment, soma stimulation conditional place aversion experiment, and in vivo LHb electrophysiology experiment. For mPFC head-fixed fiber photometry experiments, we used 400 μm core diameter (0.5 NA, Thorlabs GmbH, Dachau/Munich, Germany) fibers, which were implanted into the brain with the tip at the coordinates listed in Table S5. In all other behavioral experiments, optic fibers had a 105 μm core diameter (0.22 NA, Thorlabs GmbH, Dachau/Munich, Germany).

For secure fixture of the implantable optic fiber and for fixing the head-plate for head-fixed experiments, we disinfected the surface of the skull and dried it with 70% ethanol, then a three component Super-Bond (Sun Medical Kft., Hungary) was applied onto the skull and finally a dental cement (Paladur, M + W Dental, Hungary) was added between the Super-Bond and the base of the ceramic ferrule of the fiber implant. The positions of the optic fibers are illustrated in Fig. S16. After the surgeries, mice received 0.3–0.5 ml saline and 0.1 mg/kg meloxicam (Metacam, Boehringer Ingelheim, Germany) intraperitoneally, and were placed into separate cages until the start of the experiments.

### Surgical procedure for the freely moving fiber photometry experiments of MRR^vGluT2^ neurons

We performed surgeries when mice were between 9 to 10 weeks of age. Mice were anesthetized using isoflurane (3 to 5% for induction, 1 to 2% for maintenance; Attane, Provet) in 95% O_2_ (Praxair) and fixed in a stereotactic frame (Kopf Instruments). We used injections of buprenorphine (Temgesic, Indivior UK Limited; 0.1 mg/kg of body weight) subcutaneously, 30 min before anesthesia. For local anesthesia, we used a mixture of lidocaine (lidocaine HCl, Bichsel; 10 mg/kg of body weight) and ropivacaine (Naropin, AstraZeneca, 3 mg/kg of body weight) injected under the scalp. Postoperative pain medication included carprofen (Rimadyl, target dose: 10 mg/kg body weight, applied in the drinking water, one day before surgeries and 3 days post-operatively). Ophthalmic ointment (Corneregel or Artelac gel, Bausch&Lomb) was applied to avoid eye drying. The body temperature of the experimental animal was maintained at 35–37 °C using a feedback-controlled heating pad.

400 nl of AAV2/1-hSyn-FLEX-GCaMP6f-WPRE-SV40 (3.10 × 10^13^ GC/ml, AV-1-PV2822, UPenn Vector Core) was injected into the MRR using a precision micropositioner (Model 2650, Kopf Instruments) and pulled glass pipettes connected to a Picospritzer III microinjection system (Parker Hannifin Corporation). To avoid unnecessary bleeding and compression of liquor spaces in the midline, the virus was injected at a 15° angle tilted laterally at the following coordinates from bregma: AP: –4.30 mm; ML: +/–1.25 mm; DV: –4.50 to –4.70 mm. During the same surgery, a sterile needle was used to make an incision above the imaging site and a custom-made optic fiber connector (FP400URT, 0.50 NA, Ø400 μm; Thorlabs) was implanted in the MRR. The optical fiber was subsequently lowered into the brain using a precision micro-positioner (coordinates from bregma: 15° lateral tilt, AP: –4.30 mm; ML: +/− 1.25 mm; DV: −4.55 mm) with a custom-built fiber holder and fixed to the skull using ultraviolet light-curable glue (Henkel, Loctite 4305). For the further fixation of the optical fiber implant, a veterinary glue (Vetbond) and a blue-light curable glue (Scotchbond) were applied on the surface of the skull. A mix of dental acrylic (Paladur, Heraeus) and black acrylic paint was used to seal the skull. Mice were allowed to recover for at least 14 days after optic fiber implantation before investigating GCaMP expression.

### Perfusions for anatomy, behavioral, in vivo electrophysiology and head-fixed fiber photometry experiments

Mice were anesthetized with 2% isoflurane vapor followed by an intraperitoneal injection of an anesthetic mixture (containing 8.3 mg/ml ketamine, 1.7 mg/ml xylazine-hydrochloride, 0.8 mg/ml promethazinium-chloride) to achieve deep anesthesia. Mice were then perfused transcardially with 0.1 M phosphate-buffered saline (PBS, pH 7.4) solution for 2 min, followed by 4% freshly depolymerized paraformaldehyde (PFA) solution for 45 min, followed by PBS for 10 min, then the brains were removed from the skull.

For corticosterone-induced depression model, mice were perfused with 0.1 M PBS (pH 7.4) solution for 30 s, followed by a fixative solution containing 2% PFA and 1% glutaraldehyde (GA) and 15% picric acid for 5 min (all salts were obtained from Sigma-Aldrich); the brains were then removed from the skull on ice, cut sagittally or coronally into 5 pieces and immersion fixed in 4% PFA and 0.1% GA in 0.1 M phosphate-buffer (PB, pH 7.4) for 4 h at room temperature, then the fixative was washed out with 0.1 M PB.

After perfusion, brains were cut into 50–60 or 100 μm-thick coronal or 60–100 μm-thick sagittal sections using a vibrating microtome (Leica VT1200S or Vibratome 3000).

### Antibodies

The list and specifications of the primary and secondary antibodies used can be found in Table S6 and Table S7. The specificities of the primary antibodies were extensively tested, using either knock-out mice or other reliable methods as shown in Table S6. Secondary antibodies were extensively tested for possible cross-reactivity with the other antibodies used, and possible tissue labeling without primary antibodies was also tested to exclude auto-fluorescence or specific background labeling. No specific-like staining was observed under these control conditions. Combinations of the primary and secondary antibodies used in the different experiments are listed in Table S8.

### Fluorescent immunohistochemistry and laser-scanning confocal microscopy for anatomy, behavior, in vitro electrophysiology and head-fixed fiber photometry experiments

Perfusion-fixed sections were washed in 0.1 M PB (pH 7.4) and incubated in 30% sucrose overnight for cryoprotection. Sections were then freeze-thawed over liquid nitrogen three times for antigen retrieval. Sections were subsequently washed in PB and Tris-buffered saline (TBS, pH 7.4) and blocked in 1% human serum albumin in TBS (HSA; Sigma-Aldrich) and then incubated in a mixture of primary antibodies for 48–72 h. This was followed by extensive washes in TBS, and incubation in the mixture of appropriate secondary antibodies overnight. In some cases, we used 4’,6-diaminido-2-phenylindole (DAPI) staining (Sigma-Aldrich) to visualize cell nuclei. Then, sections were washed in TBS and PB, put on slides and covered with Aquamount (BDH Chemicals Ltd). For the behavioral optogenetic, fiber photometry, in vivo electrophysiology experiments, and for viral anterograde and retrograde tracing experiments, each injection site was validated from 50–60 µm sections using an epifluorescent Zeiss Axioplan2 microscope or a Pannoramic Midi II automatic slide scanner (3DHISTECH) equipped with a pco.edge 4.2 camera (PCO). Every part of the injected tissue containing even low levels of tracer was considered as part of the injection site. For anatomical analyses, sections were evaluated using a Nikon A1R confocal laser-scanning microscope system built on a Ti-E inverted microscope with a ×4 or ×10 or ×20 air objective or with a 1.4 NA CFI Plan Apo VC 60× oil objective both operated by NIS-Elements AR 4.3 software. Regions of interest were reconstructed in z-stacks; the distance between the focal planes was 0.3–0.5 μm for the examination of synaptic contacts and 2-3 μm for the investigation of neuronal somata. The cell and synaptic counting were performed using the NIS-Elements AR 4.3 software.

### Analysis of axonal innervation density

To compare the axonal arborization density of subcortical brain regions innervated by MRR^vGluT2^ or MRR^vGluT2→LHb^ neurons, immunohistochemistry was performed, followed by epifluorescence imaging using a Zeiss Axioplan 2 microscope. At least two coronal sections from each of the two mice per group were analyzed. Images were reconstructed and quantified using Adobe Photoshop CS6 Extended. Borders of subcortical regions innervated by MRR^vGluT2^ neurons were delineated based on the mouse brain anatomical atlas. Within each region of interest, mean pixel intensity was measured as a proxy for axonal fiber density. Background signal was measured in adjacent axon-free regions. To account for inter-section variability in baseline fluorescence, background intensity was adjusted to a comparable level across sections. For each section, background-corrected axonal density was calculated by subtracting the background intensity from the mean pixel intensity measured within the corresponding region of interest. The average axonal density values obtained for individual subcortical regions were presented as a percentage of the total axonal signal and visualized as pie charts for MRR^vGluT2^ and MRR^vGluT2→LHb^ neurons.

### Histology for the freely moving fiber photometry experiments

After completion of the behavioral experiments, mice were transcardially perfused for 2 min with PBS followed by perfusion with 4% paraformaldehyde (PFA) dissolved in PBS for 40 min. Brains were removed from the skull and cut with a VT1000S vibratome (Leica) with thickness of 100 μm. Sections were washed extensively with PBS, mounted on glass slides, coverslipped and imaged with an Axioscan Z1 microscope (Zeiss). The fiber tip positions and virus injection sites were mapped against the mouse brain atlas^75^.

### Immunoperoxidase staining and imaging for rat and macaque brain slices

Perfusion-fixed (4% PFA, 0.5% glutaraldehyde) macaque brainstems were embedded into 2% agarose and were cut into 80 μm-thick coronal sections using a vibrating microtome. These sections were washed in 0.1 M PB (pH 7.4) and incubated in 30% sucrose overnight for cryoprotection. Sections were then freeze-thawed over liquid nitrogen three times for antigen retrieval. After a subsequent wash in PB, sections were incubated in 100x citrate buffer (pH 6.0) at 80 Celsius degree for 30 min. Then sections were washed in PB (2 × 10 min) and treated with 1% sodium borohydride in PB for 15 min. After extensive washes in PB (5×5 min) endogenous peroxidase-like activity was blocked by 1% hydrogen peroxide in TBS for 10 min. Then sections were transferred into 0.05 M TBS (pH 7.4) and blocked in 2% bovine serum albumin (Sigma-Aldrich) and 0.1 g/ml lysine and glycine in TBS. Then, sections were incubated without primary antibodies (CTRL stainings) or in a solution of mouse anti-PV primary antibodies, diluted in TBS. After repeated washes in TBS, the sections were incubated in biotinylated secondary antibodies overnight followed by extensive washes in TBS (3×10 min) and in avidin–biotinylated horseradish peroxidase complex for 3 h (1:300 in TBS; Standard ABC, Vector Laboratories). The immunoperoxidase reaction was developed using ammonium nickel sulfate-intensified 3-3’-diaminobenzidine (DAB-Ni) as the chromogen. After washes in PB, sections were contrasted with 0.25% osmium-tetroxide in PB on ice for 10 min. The sections were then dehydrated in ascending ethanol series and in acetonitrile and embedded in Durcupan (ACM; Fluka). The same protocol was used for rat slices. For imaging we used Zeiss Axioplan2 microscope in brightfield mode.

### Immunogold-immunoperoxidase double labeling for array scanning electron microscopy (SEM)

Sections were rinsed in PB, cryoprotected in 30% sucrose in PB overnight, and freeze-thawed above liquid nitrogen 3 times. After extensive washes in PB and 0.05 M TBS (pH 7.4) sections were blocked in 1% human serum albumin (HSA; Sigma-Aldrich) in TBS. Then, they were incubated in a mixture of primary antibodies diluted in TBS containing 0.05% sodium azide for 3 days. After repeated washes in TBS, the sections were incubated in blocking solution (Gel-BS) containing 0.2% cold water fish skin gelatine and 0.5% HSA in TBS for 1 h. Next, sections were incubated in gold-conjugated and biotinylated secondary antibodies diluted in Gel-BS overnight. After extensive washes in TBS the sections were treated with 2% glutaraldehyde in 0.1 M PB for 15 min to fix the gold particles into the tissue. This was followed by incubation in 50 mM glycine in PB, then avidin–biotinylated horseradish peroxidase complex (Elite ABC; 1:300; Vector Laboratories) diluted in TBS for overnight at 4 °C. To enlarge immunogold particles, GoldENHANCE EM kit (Nanoprobes) was used for 5–10 min. The immunoperoxidase reaction was developed using 3,3-diaminobenzidine (DAB; Sigma-Aldrich) as chromogen.

### Sample preparation for array SEM

For array scanning electron microscopy (SEM) a modified protocol of Deerinck et al. was performed^77^. After washes in PB, sections were postfixed in 1% osmium-tetroxide reduced with 0.75% potassium ferrocyanide in PB on ice for 30 min and at room temperature for another 30 min. After extensive washes in distilled water (DW), sections were incubated in 1% aqueous uranyl-acetate in dark for 30 min. After DW washes, Walton’s lead aspartate staining was performed at 60 °C for 15 min. The Walton’s lead aspartate solution consisted of 0.066 g lead-nitrate dissolved in 10 ml of aspartic acid stock solution. The stock solution was prepared by dissolving 0.998 g L-aspartic acid in 250 ml DW, then the pH was adjusted to 5.5 with 1 N KOH. After DW washes, the sections were dehydrated through ascending concentration series of ethanol (30% EtOH 3 min, 50% EtOH 5 min, 70% EtOH 2 × 5 min, 90% EtOH 2 × 5 min, absolute ethanol 2 × 7 min), infiltrated with acetonitrile for 2 × 7 min (first on ice, second at room temperature). The sections were transferred into aluminum boats and infiltrated with embedding resin (Durcupan, ACM; Fluka) overnight. The next day, sections were mounted on glass slides, covered with coverslips, and baked at 60˚C for 48 h.

### Block preparation, serial sectioning and SEM imaging

The tissue samples containing the selected areas were cut out using a scalpel and mounted on the top of resin blocks with cyanoacrylate glue. Consecutive 70 nm-thick sections were cut with a diamond knife (Diatome, Biel, Switzerland) mounted on an ultramicrotome (EM UC6, Leica, Wetzlar, Germany) and picked up on silicon wafer (Ted Pella).

For SEM imaging, we used a FEI Apreo field emission gun (FEG) SEM (Thermo Scientific) and a T1 in column detector to record the backscattered electrons (BSE). The SEM micrographs were acquired with array tomography plugin in MAPs software (version: 3.23, ThermoFisher Scientific), which can facilitate the automatic acquisition of SEM images. The following imaging parameters were used: 4 mm working distance at high vacuum with 50 pA beam current, 2 kV high voltage, 5 μs/pixel dwell time, and 3 nm pixel size. The micrographs were 6000 × 4000 or 3000 × 4000 pixels and we recorded 100–150 sections with 70 nm thickness.

### Surgical procedures for in vivo electrophysiological experiments

Surgeries were performed under general anesthesia (isoflurane 0.5–1.5%, in 95% O2) and mice were fixed in a standard stereotactic frame (Kopf Instruments, USA or Stoelting Co., USA). Buprenorphine was injected (0.1 mg/kg of body weight subcutaneously) 30 min before surgery and lidocaine (Egis Pharmaceuticals PLC, Hungary) was applied to the scalp area before incision. Ophthalmic ointment (Vidisic, Bausch & Lomb, Canada) was used to prevent eye drying. A small lightweight headplate was attached to the skull using Paladur dental acrylic (Kulzer, Hanau, Germany). For electrophysiological experiments, where optogenetic tagging was performed, cranial windows (1.0 mm diameter) above the left or right hippocampal formation (AP, −2.5 mm; ML, ± 2.5 mm) and above the median raphe (AP, −6.1 mm; ML, 0 mm) were created under stereotaxic guidance. Holes were drilled above the median raphe (AP, −4.5 mm; ML, 0 mm) for optical fiber insertion and above the cerebellum (AP, −5.6 mm; ML, 2.0 mm left side) for ground electrode.

In mice where the median raphe - lateral habenula functional connections were investigated, craniotomies were drilled above the left and right lateral habenula (1.8 mm diameter, center position: AP, −1.8 mm; ML, ±  1.3 mm), a hole was drilled for the median raphe optical fiber (AP, −6.1 mm; ML, 0 mm) and for ground electrode (AP, −5.6 mm; ML, 2.0 mm left side).

The craniotomies and the holes were covered with fast sealant (Body Double, Smooth-On, USA). After surgery, mice were continuously monitored until full recovery, then they were returned to their home cages.

### Multichannel electrophysiological recordings in head-fixed fear conditioning and fear recall experiments

Mice were habituated to the head-fixation (downward tilted head position, pitch angle: 20°) and to a pair of custom-made tail shock electrodes daily 10–20 min for 3–6 days. Then on the first recording day, mice were head-fixed and UCLA128 channel silicon probes (Yang et al, 2020) (Masmanidis lab, UCLA 128 K, USA) were lowered through the cranial windows into the dorsal CA1 pyramidal layer and into the median raphe (in a 20° angle from caudal) under isoflurane anesthesia (0.75–1.5%, 95% O2). An optical fiber was inserted above the median raphe at a depth of 4.2 mm from skull surface (Table S5). The ground electrode was placed above the cerebellum. Probes and optical fiber were coated with lipophilic fluorescent dye, DiI (Thermo Fisher Scientific, USA) for later histological verification of the locations. Coil electrodes for tail shock delivery were pulled on the mice tail. Mice were allowed to recover from anesthesia for about 1 h before recording.

Head restrained mice were free to run, walk or sit on an air supported, free-floating, 20 cm diameter polystyrene ball. The probes in the median raphe were advanced using a micromanipulator (Inchworm Exfo, Photonic Solutions Inc, Canada). In a depth of 4400 micrometers, brief blue light pulses were applied (10 pulses at 4 Hz, pulse length: 1 ms, 473 nm, 10–15 mW, Roithner Lasertechnik GmbH, Austria) to the median raphe to identify responsive units (MRR^vGluT2^ neurons) at the beginning and end of each recording session. Recording was commenced when “tagged” units appeared. During the recordings, 12 tones were presented (20 s, 7.5 kHz, 60–80 s intertrial intervals, 70 dB intensity). The 9–12 tones were co-terminated with tail shocks (2 × 2 ms, 1 mA pulses at 10 Hz, DC Shocker, Supertech Instruments UK Ltd). On the second recording day probes and optical fiber were lowered the same way as described above (Table S5). MRR^vGluT2^ neurons were identified by optogenetical tagging as detailed above. Tones were replayed without applying tail shocks. On both recording days the mouse’s pupil was continuously illuminated by an infrared LED and imaged by a video camera (Basler AG, Germany). Pupil diameter was used as a behavioral readout for tone-fear association (first day) and fear memory recall (second day).

### Multichannel in vivo electrophysiological recordings in MRR^vGluT2^ → LHb experiments

Mice were habituated to head-fixation for 2 days then freely moving animals underwent cued fear conditioning paradigm. The conditioning context “A” consisted of a rectangular 25 × 25 cm arena with blue striped walls with custom made metal grid floor (for mild foot shocking), scented with 1% acetic-acid, and bright light conditions. The recall context “B” had round shape, green dotted walls, smooth floor, an environment scented with arganoil soap, and dim light conditions. On the first day of the behavioral paradigm, mice were placed in context “A” in a custom made sound attenuated chamber. After a 3 min baseline period, 5 times 20-second-long tones (7.5 kHz) were delivered, each co-terminated with a 2-second-long, 0.5 mA foot-shock. 30 seconds after the last foot-shocks, animals were removed from context “A” and placed in their home cages. On day 2, we lowered two 128 channel silicone probes (Masmanidis lab, UCLA 128DN, USA) into the anesthetized (isoflurane 0.75 – 1.5%, 95% O2), head-fixed animals (at 14 degree angle from lateral) into both lateral habenulae identified by emerging unit activity below the dentate gyrus. An optical fiber was inserted above the median raphe (Table S5) using micromanipulators (Luigs & Neumann, Germany). Probes and optical fibers were coated with lipophilic fluorescent dye, DiI (Thermo Fisher Scientific, USA) for later histological verification of the locations. The ground electrode was placed above the cerebellum (Table S5). Mice were allowed to fully recover from anesthesia for about 1 h before recording. During the recordings 10 tones were presented (20 s, 7.5 kHz, 60-80 s intertrial intervals, 70 dB intensity) for head-fixed fear memory recall. Each tone’s first or second half was paired in an alternating way with 10 s long laser illumination (589 nm, 8 mW, Laserglow Technologies, Canada) to the median raphe. Following the head-fixed electrophysiological recording, the animal was removed from the setup and placed back in the home cage for 1 h. Then animals were placed in context “B” and after a 3 min baseline period 5 tones were presented (20 s, 7.5 kHz, 60-80 s intertrial intervals, 70 dB intensity) to test cued fear recall in freely moving animals.

In vivo electrophysiological recordings were performed by a signal multiplexing head-stage (RHD 128, Intan Technologies, USA) and an Open Ephys data acquisition board (Open Ephys, USA). Signals were acquired at 20 kHz sample/s (data acquisition software: Open Ephys v0.4.4.1 or v0.6.3). Mouse locomotor activity on the ball was monitored with an optical computer mouse positioned close to the polystyrene ball at the equator. A custom-built microprocessor-based (Arduino) behavioral control system controlled the delivery of laser stimulation, tones and tail shocks.

### Spike sorting and neuron classification in in vivo electrophysiological experiments

Neuronal spikes were detected and automatically sorted from the high pass filtered (0.3–6 kHz) recordings by a template matching algorithm using the Spyking Circus software^78^, followed by manual curation of the clusters using the Phy software^79^ to obtain well-isolated single units. Multiunit or noise clusters were discarded from the analysis. Spike sorting quality was assessed with refractory period violation and visual inspection of auto- and cross correlations. Poor quality clusters were discarded. In MRR^vGluT2^ neuron optogenetic tagging experiments, units were categorized as “tagged” if their response followed the laser pulse with short latency (within 4 ms) and more than 50 % success rate^31^.

### Quantification and statistical analysis of in vivo physiological experiments

All in vivo electrophysiological and pupillometry data were analyzed by custom procedures written in Igor Pro 9 (Wavemetrics Inc, USA). For z-score normalization the mean baseline firing rate values and standard deviations were calculated from a 5 s time window preceding stimulus onset. All traces on figures indicate mean ± s.e.m. unless stated otherwise.

In MRR^vGluT2→LHb^ neurons in vivo electrophysiological experiments, for each LHb neuron, the average firing rates were calculated for the 10 s long half tone epochs (total of 20 half tone epochs for each neuron). The half tone epochs with and without MRR laser illumination were compared pairwise (Wilcoxon’s signed-rank test) for each LHb neuron. LHb neurons with significantly decreased firing rate upon laser illumination were analyzed (29/153).

A brief rebound activation we observed could potentially influence the results, however only when inhibition occurred during the first 10 s of the cue presentation. Although such short rebounds were occasionally observed, they did not affect the conclusions. Specifically, neuronal activity during the 10 s no-light periods did not differ depending on whether these periods occurred in the first or second half of the cue presentation (z-scored firing rate, first half: 0.062 [ − 0.007, 0.167]; second half: 0.068 [0.014, 0.113]; Wilcoxon signed-rank test, *p* = 0.83, *n* = 29 units from 8 mice). Additionally, firing rate reduction was not different between these paradigms (first half: −0.150 [−0.231, −0.089], second half: −0.127 [−0.218, −0.061]; Wilcoxon signed rank test; *p* = 0.27, *n* = 29 units from 8 mice).

For fear memory recall in freely moving animals, freezing behavior was quantified using custom-written Igor Pro 9 procedures. Freezing was defined as no movement for an at least 1-second-long period and was expressed as a percentage of time spent freezing. All statistical analyses were performed with standard Igor Pro 9 functions. For comparisons, Mann-Whitney- and Wilcoxon-test were used (Table S3).

### Fiber photometry of MRR^vGluT2^ neurons in freely moving mice

A modified Doric Fiber Photometry system (Doric) was used to perform the recordings as described previously^80^. Briefly, two different excitation wavelengths were used: 465 nm for Ca^2+^-dependent GCaMP6s activity and 405 nm to record an isosbestic, Ca^2+^-independent reference signal, the latter of which served to correct for photobleaching and movement-related artifacts. Data were preprocessed and analyzed using custom programs written in MATLAB (2017b, MathWorks). Data with obvious motion artifacts in the isosbestic channel were discarded. Demodulated raw Ca^2+^ traces were downsampled to 1 kHz and then de-trended using a low-cut filter (Gaussian, cutoff of 2 to 4 min) to correct for slow drift of the baseline signal due to bleaching. Filtered traces were z-scored by the mean and SE of the entire trace. During fear conditioning, the timing control of conditioned (CS) and unconditioned stimuli US, video acquisition for animal behavior monitoring, and synchronization with photometry recording were achieved using custom-written Python and Bonsai^81^ scripts (https://github.com/nikolaskaralis/ OE_Bonsai_network_sync). The peristimulus histograms were z-scored based on a 4-second-long baseline period preceding the tone onset. All traces on figures indicate mean ± s.e.m. unless stated otherwise.

### Fear conditioning during fiber photometry of MRR^vGluT2^ neurons in freely moving mice

Two different contexts were used. Context “A” (conditioning context) contained a clear square chamber (26.1 cm by 26.1 cm) with an electrical grid floor (Coulbourn Instruments) for mild foot shock delivery. Context “A” was in a sound-attenuating chamber under bright-light conditions and was scented and cleaned with 70% ethanol. Context “B” (retrieval context) consisted of a clear cylindrical chamber (diameter: 23 cm) with a smooth floor, placed in a dark-walled, sound-attenuating chamber under dim-light conditions. The chamber was scented and cleaned with 1% acetic acid. A stimulus isolator (ISO-Flex, A.M.P.I.) was used for the delivery of mild foot shocks. Both chambers contained overhead speakers for the delivery of auditory stimuli (conditioning stimuli, CS), which were controlled using custom Python code. We used infrared cameras for tracking animal behavior. Custom python code was used to generate precise TTL pulses to control behavioral protocols, and all the TTL signals were recorded by an OpenEphys device to synchronize behavioral protocols and behavioral tracking. Mice were extensively handled and connected to the optic fibers for 5 minutes each day, for at least 3 days, before starting fear conditioning. On day 1 (habituation day), mice were placed into context “B” and were exposed to blocks of CS- or CS+ (white noise or a pip frequency of 7.5 kHz, respectively; 75 dB sound pressure level, total CS duration of 30 second, consisting of 50-ms pips repeated at 0.9 Hz (27 pips per CS presentation, 1100 ms interpip interval; inter-trial interval: 20–180 s). On day 2 (fear conditioning session), mice were placed in context “A” and, after a 2-min baseline, subjected in an interleaved order to five CS- and five pairings of the CS+ with the US (1-sec, DC of 0.65 mA applied back-to-back after the last pip). Animals remained in the context for 1 min after the last CS presentation and were then returned to their home cages. On day 3 and day 4 (memory extinction sessions), after a 2 min baseline period in context “B”, first the CS- was presented five times then the CS+ was presented twenty-five times (inter-trial interval of 20–70 s). The first five presentations of the CS+ on day 3 were considered as fear retrieval trials.

### Fiber photometry experiments of the mPFC→MRR pathway in head-fixed mice

Fiber photometry experiments were conducted in a custom built, head-fixed-mouse setup described in detail before^82^. After optic fiber implantations, mice were transferred to the animal facility to rest. After full recovery, they received 5 days of handling. Animals were used in a few consecutive experiments. Each experimental day started with head-fixation of the mice under a brief isoflurane anesthesia if necessary. Head-fixed mice were free to run, walk or sit on an air supported, floating, 20 cm diameter polystyrene ball. Each day a 2 to 3 min long accommodation and photobleaching period preceded the measurements. Between each stimulus, an inter-stimulus interval lasted 20 to 30 s, randomly, unless noted otherwise (see below). On experimental day 6, mice were exposed to 0.2-second-long airpuffs (13 events per mouse). On experimental day 7, mice received ten-second-long auditory tone cue events (7500 Hz, 60 dB, 20 events per mouse), then 0.2 second-long LED light events (10 events per mouse). On the following 3 days, mice underwent a three-day-long cued fear conditioning paradigm, that was not evaluated due to technical reasons. Next, mice “A” and “B” were allowed to rest for two weeks, because they were later measured in a separate cohort. Finally, on experimental day 8, mice were exposed to airpuff trains. Each train consisted of ten 0.2 s-long airpuffs with an about 0.6–1.4 seconds long intervals between airpuffs (randomized in mice “A” and “B”, in all other mice this was fixed to 1 s). Inter-train intervals were 30–40 s long (randomized). Trains were repeated ten times (100 airpuffs in total per mouse).

### Data processing for fiber photometry of the mPFC→MRR pathway in head-fixed mice

In vivo fiber photometry was used to record cell type specific dynamic activity using a setup built from commercially available parts including Doric Neuroscience Studio 5.4.1.23 (Doric Lenses), Fiber Photometry Console (Doric Lenses), a 2 channel LED driver (Doric Lenses), 405 nm and 465 nm connectorized LEDs (Doric Lenses), Minicube with built in amplifier (Doric Lenses), and a 400 μm fiber patch cord (FP400URT Thorlabs). The fiber photometry setup was connected to the optic fiber that was implanted into the brain using a ceramic ferrule (Thorlabs) and a ceramic split mating sleeve (Thorlabs). Sinusoidal light intensity modulation was used to make cells emit activity dependent fluorescent signal (excitation 465 nm, 40 μW at fiber tip, 208.616 Hz modulation) and a theoretically activity-independent control isosbestic signal [excitation 405 nm isosbestic wavelength, 4 μW at fiber tip, 572.205 Hz modulation,^83^] simultaneously. The emitted green light was detected by a photodiode, amplified, converted to a digital signal with 12048 Hz sampling frequency, lock-in amplified by the data acquisition software and saved in a file for offline analysis. Digital 5 V signals were registered along with the neural recording to ease post-hoc synchronization of different events.

Data processing was done with custom written MATLAB (2023a, MathWorks) programs. Activity dependent and isosbestic signals were demodulated using the same pipeline, but independently of each other. The processing steps were designed mostly based on a previous study^84^. First, both demodulated signals were median filtered (window width = 0.0083 s) and decimated (100x, resulting in 120.48 Hz sampling rate). To remove photobleaching trend, a moving median (30 s-wide window) was subtracted from the signal. Division, instead of subtraction, yielded the same presented findings qualitatively and statistically. Since we used a head-fixed fiber setup, motion-related signal artifacts were minimal, but each recording was manually quality-checked to be artifact-free. Finally, between-session and between-subject signal variation was decreased by normalization: the detrended signal was divided by the detrended signal’s interquartile range (IQR) of the entire recording. The detrended, normalized readings were separated into peri-event sections with the help of the synchronization signals.

### Fear conditioning tests

After optic fiber implantations, mice were transferred to the behavioral unit to rest, then after recovery, they received 5 days of handling. On day 6, each mouse was placed into a plexiglass mild foot-shock chamber (25 cm × 25 cm × 35 cm) that was enriched with a specific combination of cues (“context A”): olfactory (washed with baby soap scent before each trial), visual (black and white vertical stripes on walls), spatial (rectangular chamber walls) and tactile (metal bars on the floor). Mice were allowed to freely move in the environment for 3 min and we recorded their baseline freezing levels. The rest of the experimental protocols were tailored to the specific questions specified below:

#### ArchT inhibition of the MRR^vGluT2^ neurons, MRR^vGluT2→LHb^ neuronal fibers and mPFC^→MRR^ somata during contextual fear conditioning experiments

After the exploration of context “A”, mice received mild foot-shocks (three 2-second-long, 1 mA (1.5 mA in case of the mPFC^→MRR^ soma experiment), 1 min-long inter-shock-interval). After this, mice were placed in their home cage. On day 7 and 8 mice were placed back into “context A” for 8 min. They received light exposures via the optic fiber as follows: ArchT mice received yellow light (continuous, 15 mW intensity, 593 nm LASER light power at tip), 4 minutes ON – 1 min OFF – 3 min ON. After that, mice were placed in their home cage. On day 9, mice were placed back into context “A” for 5 min without light exposure to evaluate the effect of the inhibitory treatment.

#### Inhibition of MRR^vGluT2^ neurons in cued fear conditioning experiment

After exploration of context “A”, mice received three repetitions of 30 s-long auditory cues (7500 Hz tone) co-terminated with a foot-shock (2 s long, 1 mA, 1-minute inter-shock-interval). After that, mice were placed in their home cage. On day 7 and day 8 mice were placed into a novel context (context “B”: elongated plastic chamber 40 cm  x 20 cm x 20 cm, black and white horizontal stripes on walls, plastic floor, argan oil scent). They were allowed to explore the context for 3 min. Next, they received five repetitions of the auditory cue (30 s-long, 30 s inter-cue-interval) combined with 34 s light exposures (continuous, 15 mW intensity, 593 nm LASER light power at tip) via the optic fibers starting 2 s before the cue and finished 2 s after the cue. After this, mice were placed in their home cage. In the morning of day 9, mice were placed into context “B”. They were allowed to explore the context for 3 min. Next, they received three repetitions of the auditory cue (30-second-long, 30 s inter-cue-interval) without light exposure. In the afternoon of day 9, mice were placed into context “A”. They were allowed to explore the context for 5 min while they received light exposures (continuous, 15 mW intensity, 593 nm LASER light power at tip) via the optic fiber.

#### Inhibition of MRR^vGluT2^ neurons in cued and contextual (mixed) fear conditioning experiment

The experiment was performed as described in the type (2) experiment above with some modifications. On day 7 and day 8, mice were placed into the original conditioning context (context “A”) for cued fear experiments and light exposure (34 s continuous during the cues, 15 mW intensity, 593 nm LASER light power at tip) via the optic fibers. Starting with day 9 afternoon, we could only proceed with one of the two independent cohorts, this is why there are only 7 out of the total 13 CTRL animals and 5 of the total 13 ArchT mice on Fig. 1P. In the morning of day 9, mice were placed back into context “A”. They were allowed to explore the context for 3 min. Then they received three auditory cues without light exposure, to evaluate the effect of the extinction treatment in the extinction context. In the afternoon of day 9, mice were placed into a novel context “B” (as described above). They were allowed to explore the context for 3 minutes. Then they received three auditory cues without light exposure, to evaluate the effect of the extinction treatment in a novel context.

#### TRAP2 tagging of mPFC^→MRR^ neurons during contextual fear conditioning experiment

After exploration of context “A”, all mice received foot-shocks (four 2 s long, 2 mA, 1-minute inter-shock-interval). Later, mice were placed in their home cage. After 1 h, mice received 4-hydroxytamoxifen (4-OHT, 20 mg/kg) injection intraperitoneally. After one week rest and eYFP expression period, on day 13, “ctxA” mice were placed back into context “A” for 8 min, homecage mice (“home mice”) were left in their homecage and were perfused directly from their home cage. After 1 h of context “A” exposure, “ctxA” mice were perfused for cFos immunostaining.

### Measuring general locomotor activity during contextual fear conditioning test

Animal movement was tracked using DeepLabCut^85^ to extract body keypoint coordinates from the video recordings. Data were post-processed in Python using scripts based on the movement library^86^.

### MRR^vGluT2^ activation paired to a cue to produce place aversion test

After optic fiber implantations, mice were transferred to an animal room in the behavioral unit till recovery, then they received 5 days of handling. On day 6 (habituation day), mice were placed into a box (40 cm×20 cm x 20 cm), divided into two chambers with striped walls and floor on one side and dotted walls and floor on the other side (CPA box) and were allowed to freely move for 10 min. Before each experiment, the box was washed with “macadamia nut-scented” soap. On day 7, mice were placed into a novel context “A” [plexiglass chamber (25 cm × 25 cm × 35 cm) that was enriched with a specific combination of cues: olfactory (washed with soap before each trial), visual (grey walls), spatial (square-shaped chamber walls) and tactile (metal bars on the floor)]. They were allowed to explore the context for 3 min. Next, they received eight repetitions of the auditory cue (30s-long, 30 s inter-cue-interval) combined with 15-second-long light exposures via the optic fibers (10 ms pulses at 25 Hz, 15 mW intensity, 473 nm LASER light power at tip) starting 15 s after cue onset. On day 8, mice were placed back into the CPA box. They were allowed to explore the context for 2 min. Next, if they entered the striped chamber, the cue was played until they left the chamber. The position of the mice in the CPA box was tracked using Noldus EthoVision 15 software.

### Corticosterone model of depression

This test was based on a previously published model^63^. In total, 6 groups of mice were prepared for two experiments. 1: C57Bl/6 J control (CTRL), 2: C57Bl/6 J CORT (corticosterone treated), 3: C57Bl/6 J KET (corticosterone and ketamine treated); 4: vGluT2-Cre CTRL, 5: vGluT2-Cre CORT, 6: vGluT2-Cre KET. Group 1-3 included wild type C57Bl/6 J mice, group 4–6 mice were vGluT2-Cre mice. After virus injections (only in the vGluT2-Cre mice), all mice were transferred to an animal room in the behavioral unit till recovery. Next, CORT and KET mice were exposed to corticosterone (Sigma c2505-500mg) in the drinking water from day 1 to day 20, afterwards mice received regular drinking water. The corticosterone solution was prepared by first dissolving the corticosterone in 100% ethanol (Molar Chemicals Kft. 02910-101-340), then this solution was diluted with regular drinking water to a final concentration of 0.1 mg/ml corticosterone and 0.8% ethanol. CTRL mice received 0.8% ethanol without corticosterone. On day 21, all groups received water bottles with regular drinking water. Next, on day 22, CTRL and CORT mice received an intraperitoneal saline injection, KET mice received intraperitoneal ketamine (CP-Ketamin 10% injection A.U.V., 10 mg/kg, Hungaropharma Zrt., 2302/2/07 MgSzH ÁTI) dissolved in saline. To validate this corticosterone model of depression, the cohort of C57Bl/6 J mice was tested for signs of anhedonia (see Fig.  S4A, B for results). On day 23, we carried out a sucrose preference test as described below and in ref. ^51^. The vGluT2-Cre mice were used to test the immediate effect of the ketamine injection on cFos expression in MRR^vGluT2^ neurons, thus they were not tested using the SPT but perfused on day 23 as described above.

### Unpredictable mild shock model of depression

This test was based on a previously published model^87^. After the virus injection surgery, mice were transferred to an animal room in the behavioral unit till recovery, then they received 5 days of handling. On day 6, mice were placed into a plexiglass foot-shock chamber (25 cm × 25 cm × 35 cm) that was enriched with a specific combination of olfactory (baby soap scent), visual (walls with black and white vertically stripes), geometric (rectangular chamber walls) and tactile (metal bars on the floor) cues (context “A”: CTXA). Mice were allowed to freely move in the environment for 3 min and we recorded their baseline freezing levels. After this, we carried out an “unpredictable mild shock” paradigm (UMS). FS and hM4Di mice received 19 mild foot-shocks [0.5 s long, 1.1 mA, unpredictable inter-shock-interval (ISI) length: 30 s or 60 s or 90 s] in 20 min.

### Sucrose preference test

All subjects were habituated to two leak-free water bottles in their home cage (1 mice/cage) for three days prior to testing. Then, they were water-restricted for 16 h before the test. Next day, mice were given two identical bottles, one containing regular drinking water and the other containing a 2% sucrose-in-water solution. Bottles containing sucrose and water were altered through the cages, to prevent any potential differences in consumption caused by side preference. Each mouse had access to the two bottles for at least 6 hours ad libitum. Water consumption and sucrose water consumption were calculated by weighing the bottles before and after the testing session, and sucrose preference was calculated by calculating the fraction (%) of sucrose water consumption divided by total water consumption.

### Tail suspension test

Mice were taken out of their home cages, then their tail was threaded through a plastic tube (shortened 1 ml pipette tip) to prevent tail climbing. Later, 20 cm long lab tape stripes were taped on the end of their tail to suspend mice for 6 min. Video recordings were post-hoc analyzed by an observer blind to the previous experimental treatment of the animals. Each immobile timespan was marked, where mice were motionless except for respiratory movement. Learned helplessness was quantified by the ratio of time spent with immobility.

### Chemogenetic stimulation or inhibition

To manipulate the DREADD (designer receptors exclusively activated by designer drugs) that we introduced using the AAV vectors, we gave mice an intraperitoneal injection of freshly prepared clozapine N-oxide (CNO) dissolved in physiological saline (1 mg/kg, Tocris).

### Place aversion test

After optic fiber implantations, mice were transferred to an animal room in the behavioral unit till recovery, then they received 5 days of handling. On day 6 (habituation day), mice were placed into a box (CPA box, 40 cm×20 cm x 20 cm), divided into two chambers with striped walls and floor on one chamber and dotted walls and floor on the other chamber and were allowed to freely move for 10 min. Before each experiment, the box was washed with “argan oil-scented” soap. On day 7 (place aversion test day), mice were placed back into the CPA box for 10 min. They received light exposures via the optic fibers (10 ms pulses at 25 Hz, 10 mW, 473 nm LASER light power at tip) when they entered one chamber of the box. The experiments were performed in a counter-balanced way, some mice received light exposures in the striped chamber, while others in the dotted chamber, to exclude the possibility of innate aversion/preference for any of the two chambers.

To precisely investigate, whether mice distinguished the stimulated and non-stimulated chambers in the place aversion paradigm, the box was divided into three virtual areas: an area where mice were exposed to light via fiber optics (illuminated area, ILLUM area), a non-illuminated area (“non” area) and a so-called decision zone. The decision zone was a rectangle with an area of 12 cm × 8 cm (12% of the total box area of 800 cm2) placed in the middle of the box. We excluded the time spent in the decision zone to exclude any possible uncertainty in the mouse position tracking. To define the percentage of time spent by the mice in different compartments, we divided the time spent in the ILLUM area with the sum of the time spent in the ILLUM area plus the “non” area.

### Analysis for behavioral experiments

The behavior of the mice was recorded with a Basler acA1300-60gc camcorder, and the experimental data was collected and analyzed using the Noldus EthoVision 15.0 software. Freezing behavior of mice was recorded with a custom-made freezing detection head-mounted Axonda sensor, a trained observer or ezTrack software^88^. Periods of immobility had to last at least 2 s to qualify as freezing behavior.

### In vitro electrophysiology and single cell mRNA collection

200–250 μm thick horizontal brain slices were prepared from the brainstem of vGlut2-Cre mice that were injected with AAVretro-EF1α-DIO-mCherry into the LHb 2–5 weeks prior the experiments. The mice were decapitated under deep isoflurane anesthesia and slices were cut in ice-cold cutting medium containing 85 mM NaCl, 75 mM sucrose, 2.5 mM KCl, 25 mM glucose, 1.25 mM NaH_2_PO_4_, 4 mM MgCl_2_, 0.5 mM CaCl_2_, and 24 mM NaHCO_3_ using a vibratome (VT1200S; Leica). The slices were incubated at 35 °C for about 30 min and then kept at room temperature until use. During experiments, slices were transferred to a recording chamber mounted on an upright microscope (Eclipse FN-1; Nikon). The chamber was continuously perfused with artificial cerebrospinal fluid (ACSF) at a flow rate of 2 ml/min. The ACSF composition was 126 mM NaCl, 2.5 mM KCl, 26 mM NaHCO_3_, 2 mM CaCl_2_, 2 mM MgCl_2_, 1.25 mM NaH_2_PO_4_, and 10 mM glucose, saturated with carbogen gas (5% CO_2_ and 95% O_2_). All chemicals were obtained from Sigma-Aldrich, Merck, and recordings were performed at a temperature of 35 °C. Fluorescently labeled MRR neurons were visualized using epifluorescent illumination (Lambda FLED @ 530 nm; Sutter Instrument) combined with a CMOS camera (Davinci 1k; RedShirtImaging). mCherry+ neurons were patched using thin-wall borosilicate pipettes (BF150-117-10, Sutter Instrument) with a pipette resistance of 3.86 ± 0.06 MΩ. The pipettes were filled with approximately 1 µl of intracellular solution containing 133.5 mM K-gluconate, 1.8 mM NaCl, 1.7 mM MgCl_2_, 0.05 mM EGTA, 10 mM HEPES, 2 mM Mg-ATP, 0.4 mM Na_2_-GTP, 10 mM phosphocreatine, and 8 mM biocytin (adjusted to pH 7.25; 290–300 mOsm). The recordings were made using a HEKA EPC800 amplifier (HEKA Elektronik), low-pass filtered at 10 kHz, and digitized at 250 kHz with a Digidata1440A A/D converter (Molecular Devices). The data acquisition was controlled by the pClamp10 software package (Molecular Devices). After patching the identified mCherry+ neurons, we measured the resting membrane potential, the passive electrical parameters, and the firing characteristics of the cell. For all current-clamp recordings, pipette capacitance and bridge balance were carefully compensated. The duration of the recording session was limited to 5 min from the break-in. After the recording, the cytosol together with the nucleus was extracted from the neuron by the application of small negative pressure. The fluid content of the recording pipette was then rapidly released into a microtube containing ice cold lysis buffer (10x buffer, Takara). The sample containing tubes was spun briefly and snap-frozen on dry ice for subsequent processing. To suppress RNase contamination, the experimental setup and the surrounding area were cleaned each day before the start of the sample collection with RNase AWAY (Thermo Fisher).

### In vitro electrophysiology data processing

The electrophysiological data were analyzed using Clampfit (v10.7, Molecular Devices) and custom written Python scripts (Python 3.11). “Input resistance” of the cells and “cellular capacitance” were measured in voltage-clamp mode using the response to 100-ms voltage steps from −70 mV to −90 mV. Input resistance was determined from the steady-state response, while membrane capacitance was calculated by integrating the transient capacitive component of the response. The membrane time constant was calculated by fitting a monoexponential function to the voltage response evoked by small hyperpolarizing current steps in current clamp. The “sag” was calculated as the ratio of the steady-state amplitude to the peak amplitude of the voltage response during a hyperpolarizing step (−40 pA, 1 s). Action potential (AP) properties were determined on the first AP evoked at the rheobase stimulation. The “AP threshold” was defined as the membrane voltage at which the rate of the upstroke reached 100 V/s. Using the time derivative of the membrane voltage, we also quantified the maximum rates of rise and decay (“Maximal AP rise and decay slopes”). “AP rise time” was measured as the duration of the upstroke from the AP threshold to its peak. “AP amplitude” was defined as the voltage difference between the AP threshold and its peak. The “AP half-width” was determined as the duration of the AP at half of its amplitude. “AHP amplitude” was defined as the voltage difference between the most negative point of the afterhyperpolarization and the AP threshold. “Time to AHP peak“ was measured as the duration of the repolarization from the AP peak to the most negative point of the afterhyperpolarization. To describe the firing dynamics, we selected a recorded sweep where the neuron exhibited steady firing without a depolarization block in response to 1-s-long depolarizing current step (mean firing frequency ranged from 5 to 32 Hz). We determined the maximum instantaneous firing frequency and the mean firing frequency from this sweep and the ratio of these two values was used to calculate “burstiness” (max/mean). “AP amplitude adaptation,” “half-width adaptation,” and “frequency adaptation” were defined as the ratio of each parameter (amplitude, half-width, and instantaneous frequency) of the last AP to that of the first.

### Single-cell mRNA sequencing and analysis

#### Sample collection

After electrophysiological recordings, the cytosol of the cell was aspirated via the same recording glass pipette that used for recording, and using a positive pressure, expelled into microtubes containing 1 µl of 1x lysis buffer with RNase inhibitor (SMART-Seq HT PLUS Kit, Takara). The samples were immediately frozen on dry ice and stored at −80 °C until further processing.

#### cDNA and library preparation for next-generation sequencing

Single-cell mRNA processing was performed using the SMART-Seq HT PLUS Kit (Takara) and according to manufacturer’s protocol. In brief, samples were reverse transcribed to cDNA, amplified, and purified with AMPure XP beads (Beckman Coulter). The quantity and the quality of the purified cDNA were assessed using Fragment Analyzer (Advanced Analytical). Subsequently, the cDNA was fragmented and adapters including indices for cell identification were added to the fragments. The quality and quantity were again assessed using Fragment Analyzer. For library preparation, the single cell samples with unique index adapters were pooled in equal amounts. The sequencing was performed on a NovaSeq 6000 (Illumina) instrument using 150 bp paired end configuration and a full SP-flowcell.

#### Processing of sequencing data

After sequencing, raw reads were de-multiplexed with Illumina’s bcl2fastq2 (v2.20). Read qualities were checked with fastqc. Low phred score read ends and adapters were trimmed using Trimmomatic^89^. Reads with only 3 type of bases or less were removed. STAR aligner^90^ was used to map reads to the mm39 reference genome using the v110 Ensembl annotation. Three sequences were added to the genome and annotation: Addgene: pAAV-hSyn-DIO-mCherry (Plasmid #50459); Cre (NCBI/Nucleotide:NC_005856.1); and YFP (NCBI/Nucleotide:GQ221700.1). Expression was evaluated on the level of genes. Cells that had read or gene count numbers more than three times the median absolute deviation below the median were excluded. In the Fig. S8, we presented heatmaps based on the percentage of tpm (transcripts per million) count of each genes.

### Cluster analysis of in vitro electrophysiological and genetic data

The cluster analysis was performed using the ClustVis software^91^. For clustering the in vitro electrophysiological data, we utilized 20 independent electrophysiological parameters obtained from 74 MRR^vGluT2→LHb^ neurons, which are shown in Fig. S5. For clustering the genetic data, we utilized 404 genes involved in neuronal firing and signaling processes obtained from 38 MRR^vGluT2→LHb^ neurons. Specifically, we used the top 100 genes with the largest interquartile range, measured in tpm. Both the electrophysiological and genetic data were clustered using identical settings and we used the hierarchical clustering method. Pre-processing included unit variance scaling (Z-score). For constructing the dendrograms and heatmaps, only the neurons (columns) were clustered, while the rows were left unchanged. The following settings were applied consistently for both analyses: distances - correlation-based, clustering method - average linkage, and ordering - tightest cluster first.

Clustering of in vitro electrophysiological data, including all 74 neurons and their 20 measured in vitro electrophysiological parameters, is visualized as a dendrogram and heatmap in Figure S5. We named the electrophysiological clusters as: *Type eN* and *Type eB* (*Type eN* − 40 neurons vs. *Type eB* − 34 neurons).

Clustering of genetic data, including all neuronal function related genes (presented in Figure S6) that were significantly different (using an unpaired *t*-test, *p* < 0.05) between any two clusters, is visualized as a dendrogram and heatmap in Figure S5. We named the genetic clusters by following objective criteria: the average gene expression (measured in tpm) of a gene within a given cluster was divided by its average expression across the other two clusters, then the cluster was named after the gene with the highest ratio and a statistically significant difference between clusters (using an unpaired *t*-test, *p* < 0.05). Based on this approach, the clusters were named as follows: *Type gG* (Glra1), *Type gC* (Cacng5), and *Type gH* (Htr2c).

### Neurolucida cell reconstruction

Biocytin-labeled MRR neurons that were recorded in in vitro experiments were reconstructed in 3 dimensions. We used the Neurolucida System (MBF Bioscience) to reconstruct the neuronal processes from z-stacks of images. We used a Nikon A1R confocal laser-scanning microscope system built on a Ti-E inverted microscope with a 20× air objective operated by NIS-Elements AR 4.3 software. The distance between the focal planes was 2 μm. Section shrinkage was corrected along the X, Y and Z axes.

### Statistics for behavioral, fiber photometry, in vivo, in vitro and genetic experiments

For statistical analysis, we used Matlab 2023a, GraphPad Prism 9 and Tibco Statistica 13.4 softwares. In case of data groups that did not show a Gaussian distribution, we used median and 25% to 75% interquartile range to present data and were tested using non-parametric tests. Homogeneity of variance was tested using F-test and if it was significant then populations were compared using non-parametric tests. To test for statistical differences, we used the non-parametric Mann–Whitney U-test in independent data populations, we used the Wilcoxon’s signed-rank test in nonparametric dependent data populations and T-tests in parametric dependent data populations. For nonparametric multiple comparison, we used the Kruskal-Wallis one-way ANOVA by rank test with *posthoc* Mann-Whitney U-tests. Statistical differences have always been tested using two-sided tests. For indicating significance levels in figures, we used the following standard rules, n.s. (non-significant): *p* > 0.05; *p < 0.05; **p < 0.01; ***p < 0.001; ****p < 0.0001.

### Reporting summary

Further information on research design is available in the Nature Portfolio Reporting Summary linked to this article.

## Supplementary information

Supplementary Information

Peer Review file

Reporting Summary

## Source data

Source Data

## Data Availability

The RNA sequencing data generated in this study have been deposited in the NCBI Gene Expression Omnibus (GEO) database^92^ under accession code GSE300145. All other relevant data generated in this study are provided in the manuscript and its Supplementary Information/Source Data file.  Source data are provided with this paper.

## Code availability

Custom scripts used during the work were developed for routine data processing, analysis workflows, and figure generation, using established methods and standard approaches. All experimental procedures, methods, and software used are described in the Methods section in sufficient detail to enable reproduction of the study. Core fiber photometry analysis code was deposited to Zenodo (https://doi.org/10.5281/zenodo.21026721).
