# Supplementary material for: Prefrontal-raphe-habenular circuit drives fear memory recall and updating
Source: Nat Commun. 2026 Aug 12;17:9686. doi: 10.1038/s41467-026-76574-5 (PMC13558683; doi:10.1038/s41467-026-76574-5)
Supplement: Supplementary file 1 [file 41467_2026_76574_MOESM1_ESM.pdf]

Supplementary Materials for

Prefrontal-raphe-habenular circuit drives fear memory recall and updating

Authors: Boldizsár Zsolt Balog#, Albert M. Barth#, Krisztián Zichó#, Abel Major#, Zsuzsanna Bardóczi, János Brunner, Charlotte Seng, Márta Jelitai, Katalin E. Sos, Réka Z. Sebestény, András Szőnyi, Nikolas Karalis, Áron Orosz, Eszter Sipos, Erik Misák, György Cserey, Andreas Lüthi, Viktor Varga, Csaba Földy, János Szabadics, Gábor Nyiri*

# These authors contributed equally to this work
* Corresponding author, e-mail: nyiri.gabor@koki.hun-ren.hu

This pdf file includes:

Supplementary Figs. S1 – S17
Tables S1 – S10

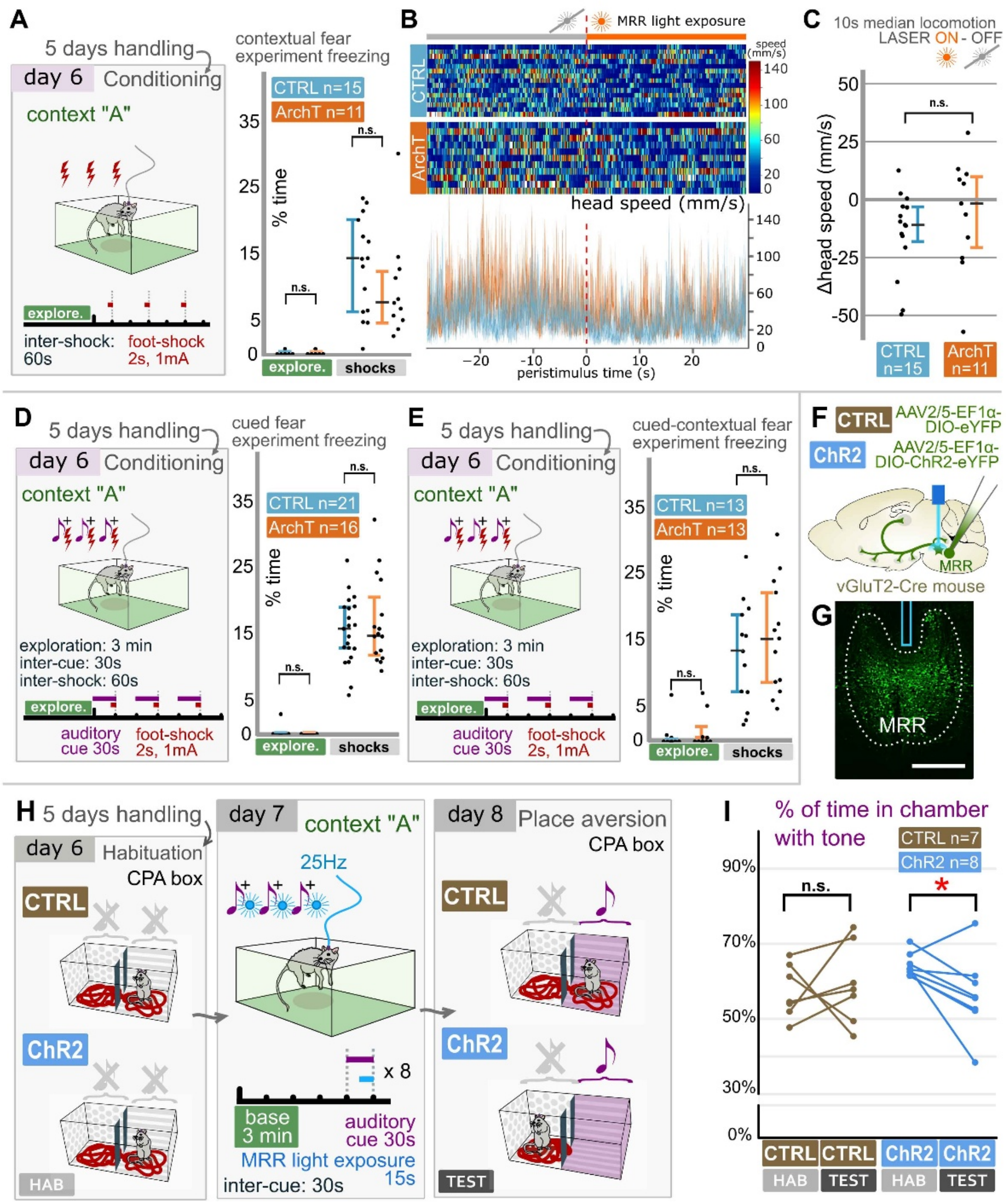

FigS1. Supplementary data for contextual and cued fear experiments.

(A, D, E) Graphs and schematics show that we found no significant differences in acute fear behavior (freezing time percentages), during conditioning in contextual (A, from 15 CTRL and 11 ArchT mice), cued (D, from 21 CTRL and 16 ArchT mice) and the simultaneous cued and contextual fear conditioning experiments (E, from 13 CTRL and 13 ArchT mice).

(B, C) No direct locomotory effect was detected after MRR light exposure was turned back on at the 5th minute during the contextual fear recall experiment. (same experiment as on Fig1C, D day7) (B) Heatmaps and peristimulus graphs show that head speed follows similar paths in CTRL and ArchT mice. (C) Graph shows no significant difference of CTRL and ArchT mice in the change

of median head speed as a response to MRR light exposure (difference of medians: 10s after LASER ON – 10s before LASER ON)

(F-I) Here, direct MRR^{vGluT2} neuronal activation was paired with an auditory cue to test whether activation of these neurons can generate an aversive memory that is later recalled by the cue. **(F)** Cre-dependent AAV was injected into the MRR of vGluT2-Cre mice. Control mice (CTRL) were injected with AAV2/5-EF1α-DIO-eYFP virus, stimulated mice (ChR2) were injected with AAV2/5-EF1α-DIO-ChR2-eYFP virus. An optic fiber was implanted over the MRR. **(G)** Fluorescent image shows eYFP expression (green) and the position of the optic fiber (blue) over the MRR. Scale bar, 500 µm. (E). **(H)** After handling, on day 6, mice were habituated (HAB) to a double-chamber (conditional place aversion, CPA) box to habituate them to the test box and we analyzed their baseline preference. On day 7, mice were placed in a novel context "A", where they received eight repetitions of 30-second-long neutral auditory cues with 15-second-long light exposure via the optic fiber at the end of each cue presentation. On day 8, mice were placed back into the dual-chamber box, where the auditory cue was played only when mice were in one of the two chambers. **(I)** Significant avoidance of the chamber with the auditory cue was detected in ChR2 mice but not in CTRL mice compared to their baseline preference (from 7 CTRL and 8 ChR2 mice).

For statistical details, see TableS1. For reconstructed optic fiber localization see FigS16. Graphs show medians and interquartile ranges. Source data are provided as a Source Data file.

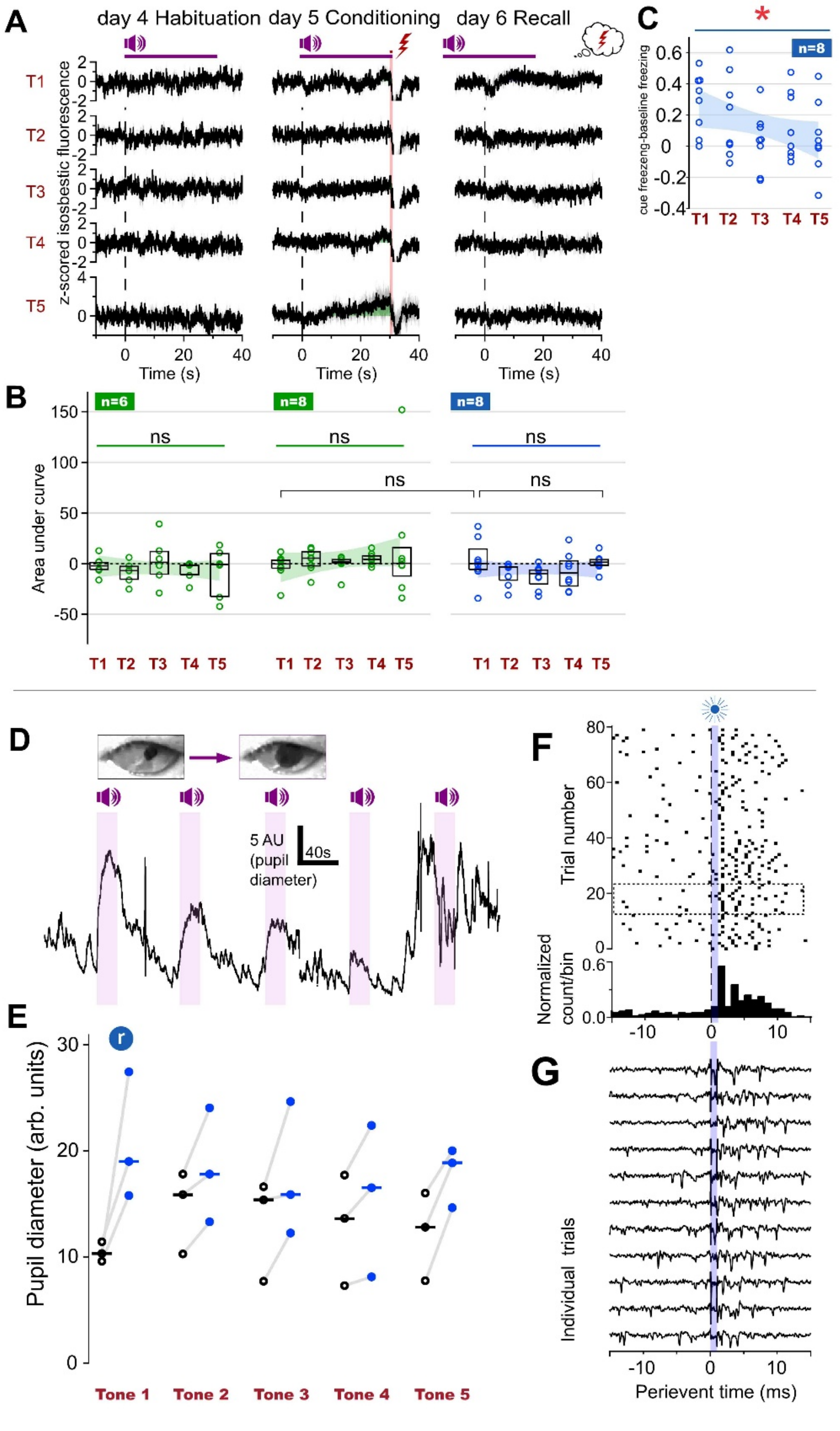

FigS2. Supplementary data for fiberphotometry and *in vivo* electrophysiological experiments

(A-B) Isosbestic control fluorescence signals for MRR^{vGluT2} neuronal fiberphotometry experiments combined with cued fear memory tests, detailed in Fig2. **(A)** Averaged peristimulus

z-scored isosbestic signals of MRR^{vGluT2} neurons (mean±SEM). **(B)** Corresponding area-under-the-curve box plots (for the 30s auditory cue period). Significant regression was absent both before and during the cued fear memory recall period (shaded areas indicate the 95 % confidence interval for linear regressions, Habituation: from 6 mice, Conditioning: from 8 mice, Recall: from 8 mice). **(C)** Baseline-corrected freezing levels show a significant negative regression across cue presentations. **(D-G)** Pupil diameter and optogenetical tagging recordings for MRR^{vGluT2} in vivo electrophysiology experiments combined with cued fear memory tests detailed in Fig2. **(D)** Pupil diameter trace during cued fear memory recall in a sample animal. Vertical shaded areas indicate the auditory cue presentations. **(E)** Average pupil diameter values for all animals during cued fear memory recall (black: 20 s baseline before auditory cue, green: during auditory cue presentations, horizontal lines indicate median values). The amount of dilation shows a decreasing trend across repeated auditory cue presentations. **(F)** Raster plot (top) with corresponding normalized, peristimulus firing rate histogram of a sample optogenetically tagged unit. **(G)** Recording sweeps of consecutive optogenetical tagging trials (indicated by dashed rectangle on E) of the unit in E. Vertical blue shaded areas on E-F indicate the 1 ms-long blue laser stimulation.
For statistical details, see TableS2. Graphs show medians and interquartile ranges unless specified otherwise. Source data are provided as a Source Data file.

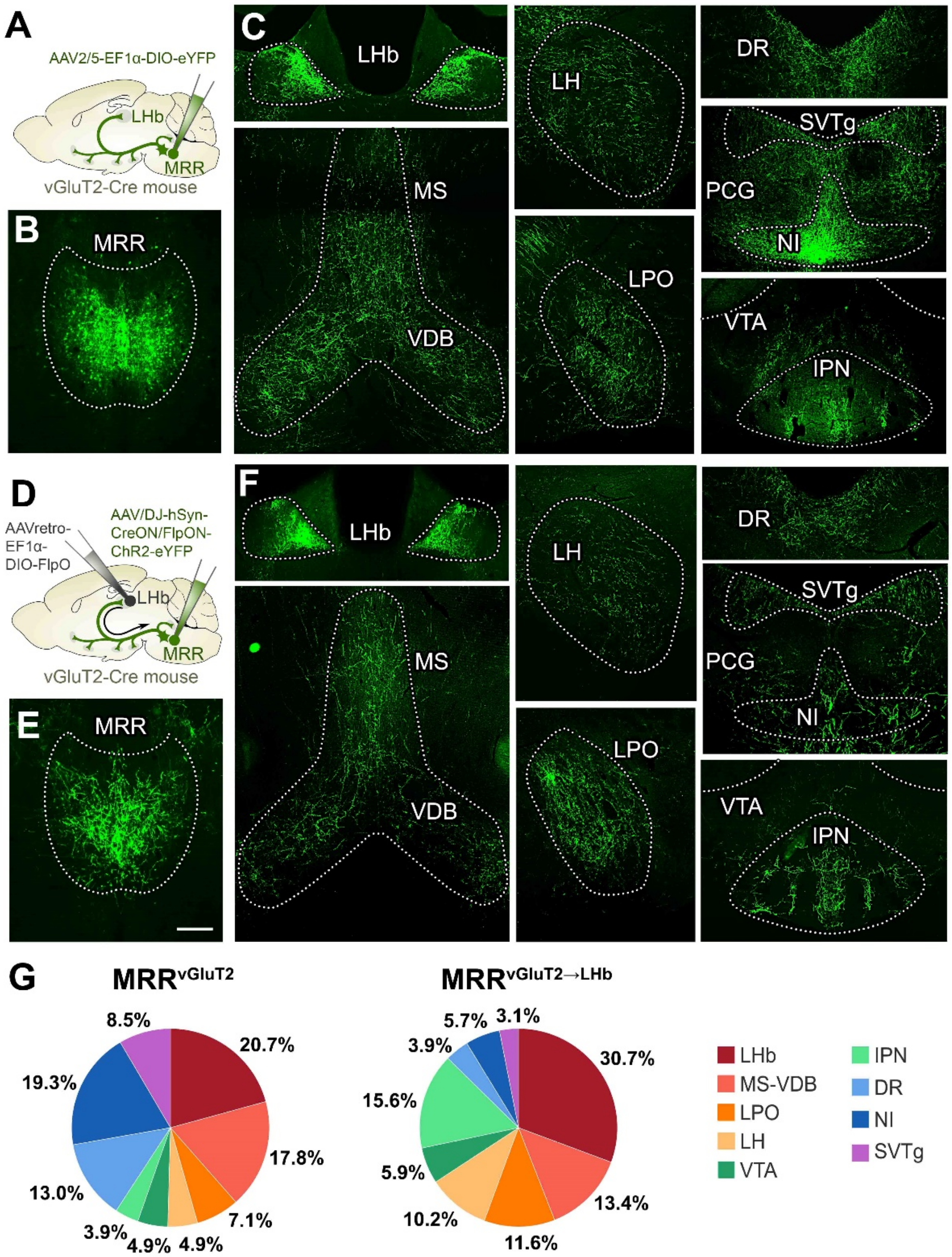

FigS3. Target selectivity of MRR^{vGluT2} and specifically MRR$^{vGluT2 \rightarrow LHb}$ neurons

(A) Cre-dependent eYFP-encoding AAV was injected into the MRR of vGluT2-Cre mice. **(B)** Fluorescent image shows the injection site in the MRR. **(C)** Some of the most prominent targets of MRR^{vGluT2} neurons were the lateral habenula (LHb), the ventral tegmental area (VTA), the medial septum (MS), the vertical limb of the diagonal band of Broca (VDB) and the nucleus incertus (NI). Other areas were also targeted including the interpeduncular nucleus (IPN), the dorsal raphe (DR), the lateral hypothalamus (LH), the lateral preoptic area (LPO), the pontine

central gray (PCG) and the subventricular tegmental nucleus (SVTg). **(D)** To selectively label $MRR^{vGluT2\rightarrow LHb}$ neurons, a Cre-dependent, Flp-encoding, retrograde AAV was injected bilaterally into the LHb of vGluT2-Cre mice. In the same mice, Cre-dependent and Flp-dependent, eYFP-encoding AAV was injected into the MRR. **(E)** Fluorescent image shows $MRR^{vGluT2\rightarrow LHb}$ neurons labeled using intersectional viral tracing method. Scale bar, 200 μm for all images. **(F)** The axon collaterals of $MRR^{vGluT2\rightarrow LHb}$ neurons were similar to that of all MRR^{vGluT2} neurons, but showed sparser axonal arborization in the VTA and in the NI. **(G)** Two pie charts show the regional distribution of axonal projection density from MRR^{vGluT2} and $MRR^{vGluT2\rightarrow LHb}$ neurons. Each chart depicts the percentage contribution of the mean axonal pixel intensity measured in individual target regions relative to the total summed pixel intensity across all analyzed regions.

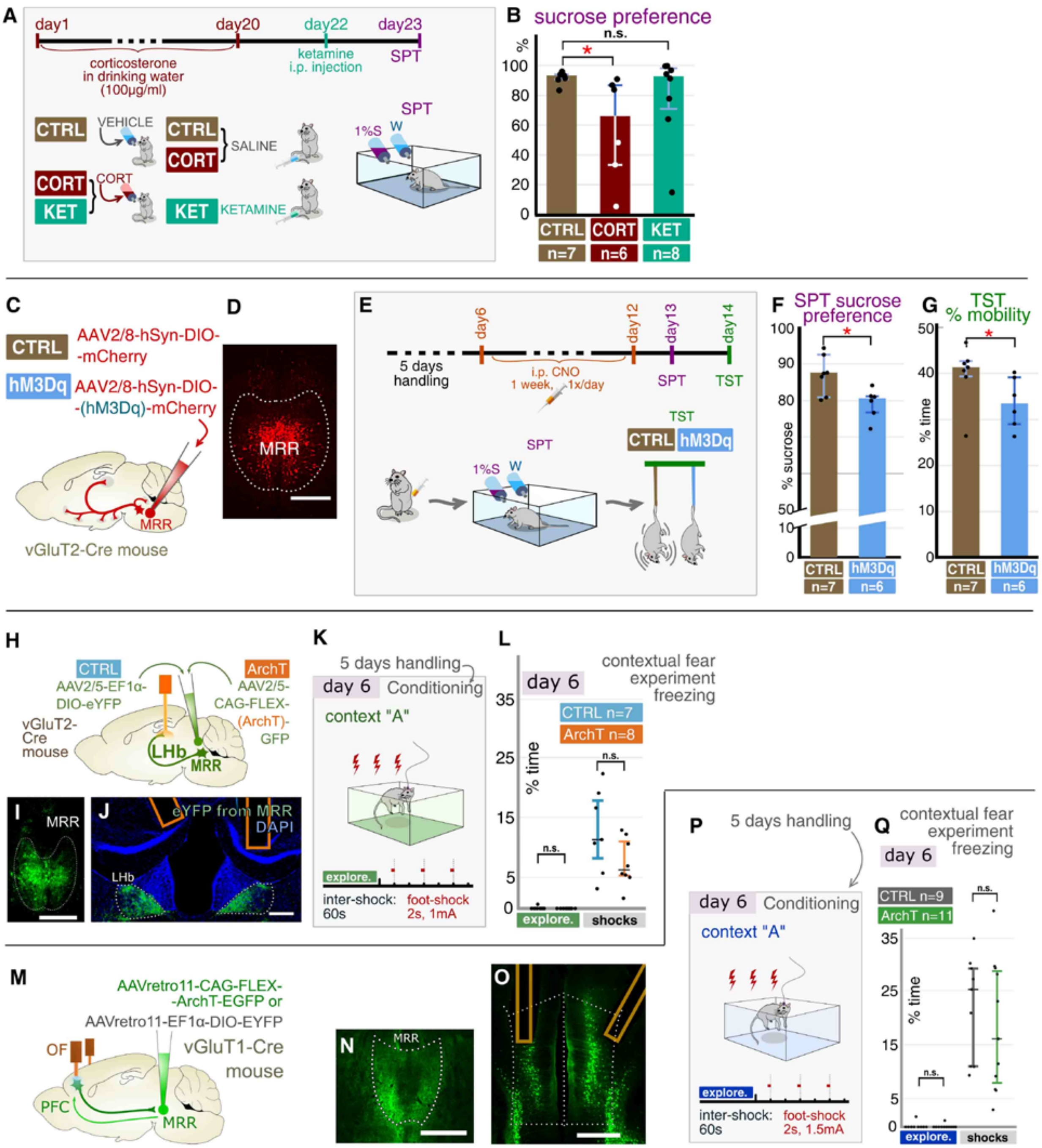

FigS4. Supplementary data for confirming the role of MRR^{vGluT2} neurons in depression models.

(A) Mice received corticosterone (100 µg/ml in drinking water) for 3 weeks (CORT and KET groups), CTRL mice did not. One day later, KET mice received ketamine (10 mg/kg, intraperitoneally), CTRL and CORT mice received saline. Sucrose preference test (SPT) was

subsequently performed. **(B)** Sucrose preference was lower in CORT mice compared to CTRL mice and restored to CTRL levels by a single ketamine treatment, validating the depression model and ketamine effect. **(C)** Cre-dependent excitatory hM3Dq AAV (hM3Dq mice) or control AAV (CTRL mice) injection into the MRR of vGluT2-Cre mice. **(D)** Fluorescent image showing MRR^{vGluT2} neurons (red). Scale bar, 500 µm. **(E)** Mice received daily CNO injections (1 mg/kg, i.p.) for 1 week, followed by SPT and tail suspension test (TST). **(F-G)** hM3Dq mice showed reduced sucrose preference and lower mobility during TST compared to CTRL mice. **(H–L)** Control experiment for Fig3N–R. **(H-I)** Cre-dependent inhibitory ArchT AAV (ArchT-GFP mice) or control AAV (CTRL mice) injection into the MRR of vGluT2-Cre mice. Scale bar, 500 µm. **(J)** Bilateral optic fiber (orange) implantation above the $MRR^{vGluT2 \rightarrow LHb}$ fibers (green) with DAPI (blue). Scale bar, 200 µm. **(K)** Days 1-5: handling; day 6: three foot-shocks (2 s, 1 mA) in context "A". **(L)** No significant differences in acute freezing behavior were observed (n_{CTRL}=7, n_{ArchT}=8). **(M-Q)** Control experiment for Fig9A-E. **(M-N)** Cre-dependent, retrograde inhibitory ArchT AAV (ArchT-GFP mice) or control AAV (CTRL mice) injection into the MRR of vGluT1-Cre mice. Scale bar, 500 µm. **(O)** Bilateral optic fiber (orange) implantation above the mPFC→MRR neurons (green). Scale bar, 200 µm. **(P)** Days 1-5: handling; day 6: three foot-shocks (2 s, 1.5 mA) in context "A". **(Q)** No significant differences in acute freezing behavior were observed (n_{CTRL}=9, n_{ArchT}=11).

Graphs show medians and interquartile ranges. For statistical details, see TableS1. Source data are provided as a Source Data file.

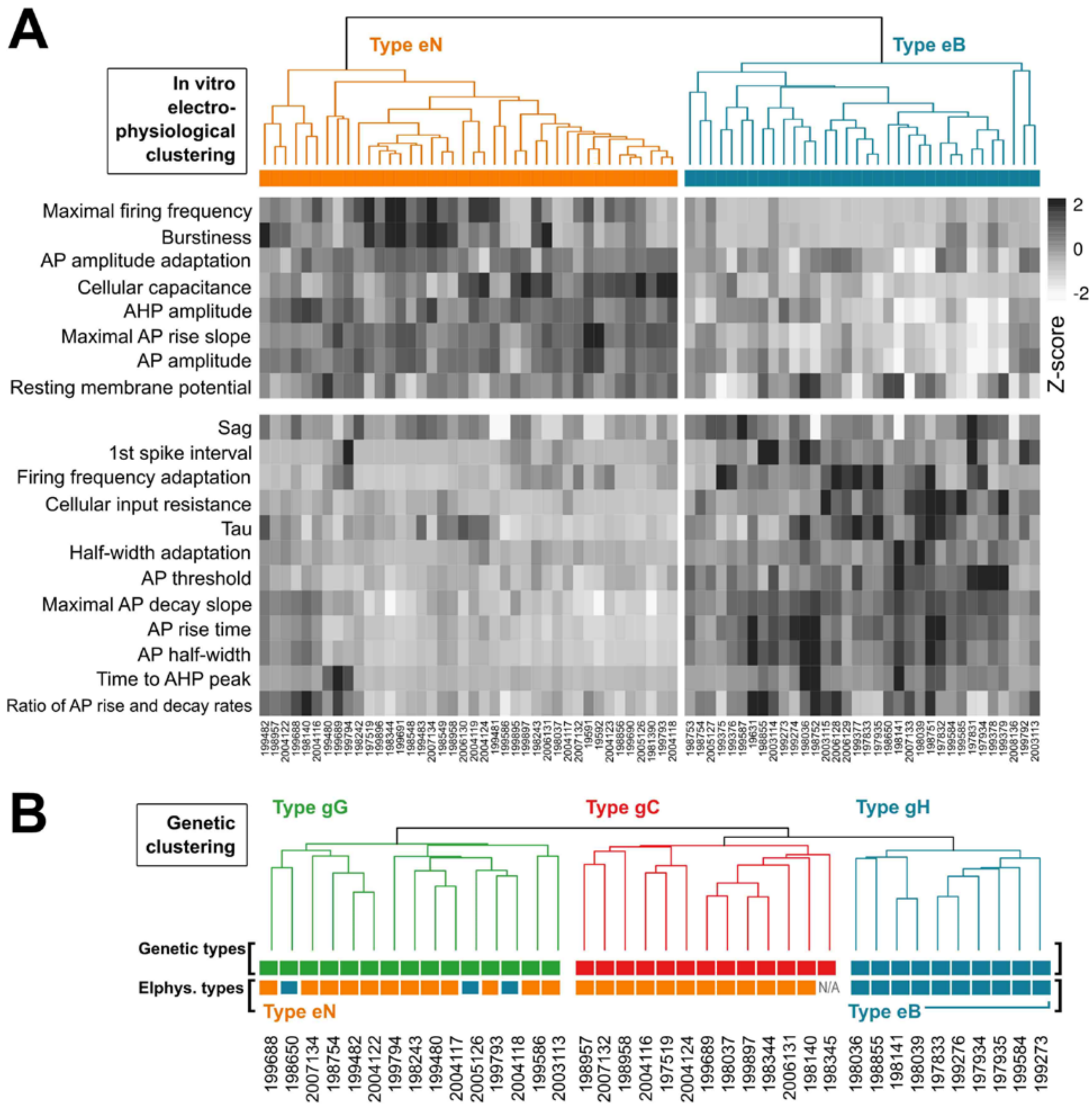

FigS5. Hierarchical cluster analysis of electrophysiological and gene expression data of MRR$^{vGluT2\rightarrow LHb}$ neurons.

(A) Dendrogram of hierarchical clustering (distances: correlation based, method: average, ordering: tightest cluster first) based on 20 electrophysiological parameters of MRR$^{vGluT2\rightarrow LHb}$ neurons. Two clusters were identified: *Type eN* (orange) and *Type eB* (blue), see Fig5, FigS6 and Results for details on clustering. The heatmap shows the differences (presented as z-scores) in 20 *in vitro* electrophysiological parameters between the two clusters. Neuron IDs are at the bottom. **(B)** Hierarchical clustering of genetic data (distances: correlation based, method: average,

ordering: tightest cluster first) based on 100 genes with the highest variability (highest interquartile ranges) among all neuronal-function-related genes. We found 3 clusters: *Type gG* (green), *Type gC* (red), and *Type gH* (blue), see Fig5, FigS6 and Results for details. Neuron IDs are at the bottom. ClustVis software [91] was used for cluster analysis and visualization. For cluster analyses, see Methods. For electrophysiological properties without z-scoring, see TableS9. For an overview of measurement participation of each individual neuron see TableS10.

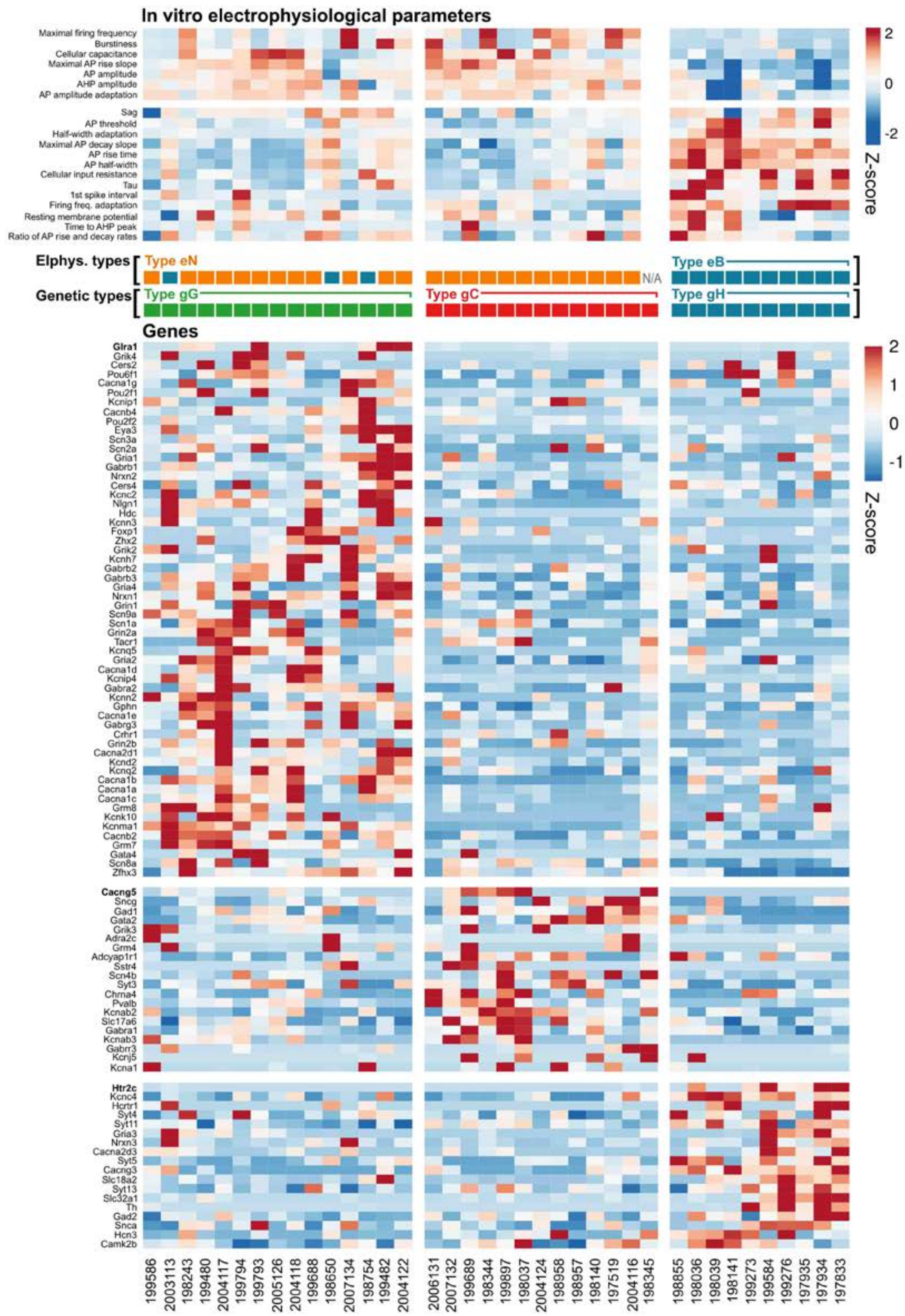

FigS6. Extended data for clustering of MRR$^{vGluT2\rightarrow LHb}$ neuronal subtypes.

Upper heatmaps show the z-score differences in 20 electrophysiological parameters and lower heatmaps show differences in 96 gene expressions among the MRR$^{vGluT2\rightarrow LHb}$ neurons. The

heatmap includes all typical neuronal-function-related genes that were significantly different (using an unpaired t-test, $p < 0.05$) between any two clusters. Between the tables of heatmaps, color boxes represent subtypes based on the electrophysiological and the genetic screening of $MRR^{vGluT2 \rightarrow LHb}$ neurons, see Fig5, FigS5 and Results for details. Individual neuron IDs are shown at the bottom of the heatmaps. We used the ClustVis program for cluster analysis and visualization [91]. For details of the clustering, genes and electrophysiological properties selection see Methods. For electrophysiological properties without z-scoring, see TableS9. For an overview of measurement participation of each individual neuron, see TableS10.

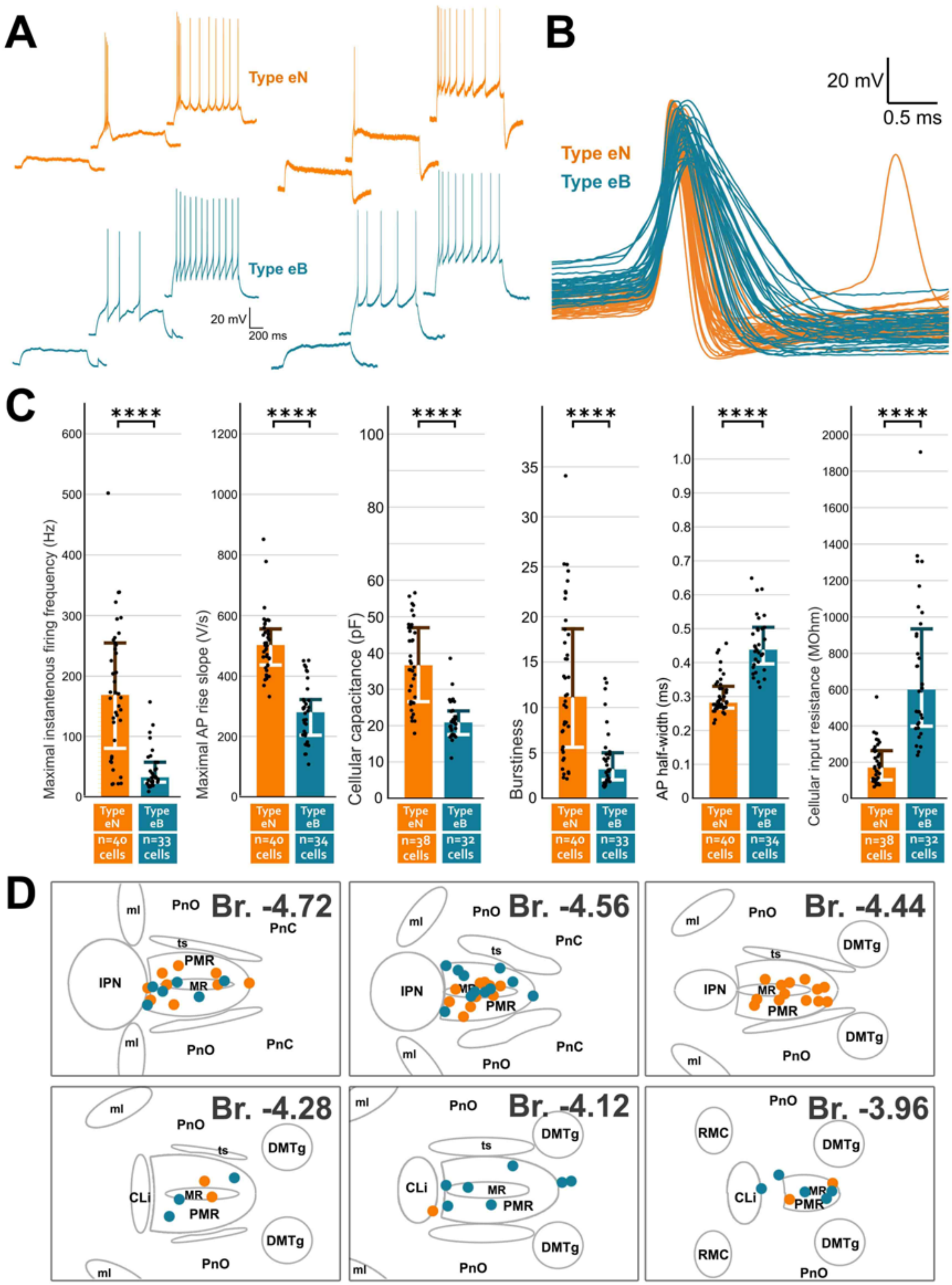

FigS7. Extended electrophysiological data and anatomical localization of MRR$^{vGluT2\rightarrow LHb}$ neurons.

(A) Firing properties from two representative *Type eN* (orange) and two representative type B (blue) MRR$^{vGluT2\rightarrow LHb}$ neurons, see Fig5, FigS5, FigS6 and Results for details. **(B)** Representative action potentials. **(C)** Graphs show significant differences between *Type eN* and *Type eB* clusters

in some electrophysiological properties. **(D)** Localization of $MRR^{vGluT2 \rightarrow LHb}$ neurons (recorded in horizontal *in vitro* slices from Bregma dorsoventral -3.96 mm until -4.72 mm) was not subtype-specific. Orange dots represent *Type eN* neurons, blue dots represent *Type eB* neurons (see FigS9 for morphological details).

Graphs show medians and interquartile ranges. For statistical details, see TableS3. For an overview of neurons in each analysis see TableS9-10. Source data are provided as a Source Data file.

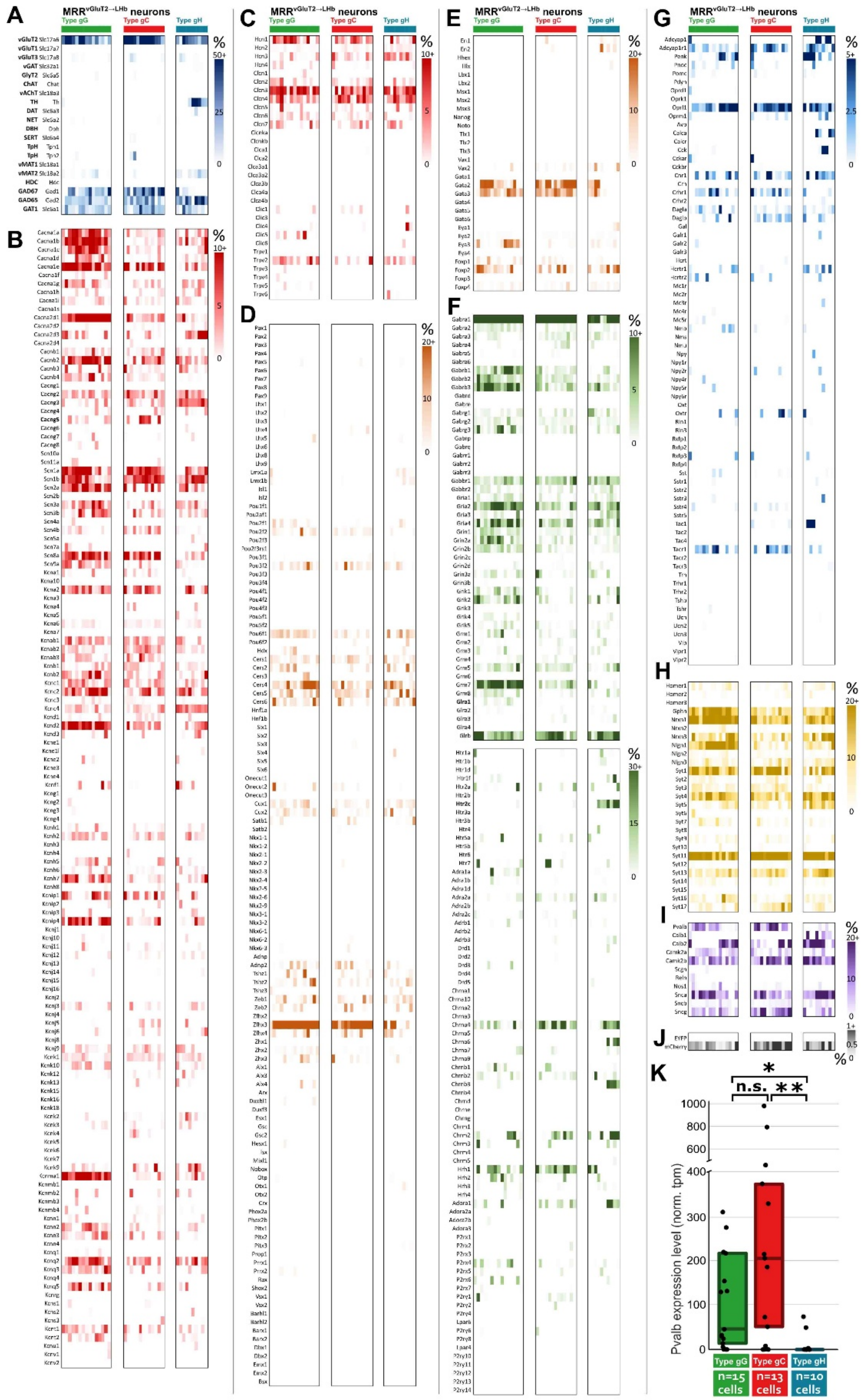

FigS8. Supplementary data for the expression of selected genes of MRR$^{vGluT2\rightarrow LHb}$ neurons. Heatmaps represent relative expression (percentage of tpm count, for details see Methods) of mRNAs collected from 38 individual MRR$^{vGluT2\rightarrow LHb}$ neurons. Three clusters: *Type gG* (15

neurons), *Type gC* (13 neurons) and *Type gH* (10 neurons), see Fig5, FigS5, FigS6 and Results for details. Heatmaps represent relative expression (in the percentage of tpm) of **(A)** major neurotransmitter-related genes (dark blue), **(B-C)** ion channel genes (red), **(D-E)** homeobox and transcription factor-related genes (orange), **(F)** major neurotransmitter-receptor genes (green), **(G)** neuropeptides and their receptor genes (blue), **(H)** synaptic protein genes (yellow), **(I)** calcium-binding and other neuronal protein genes (purple), and **(J)** test marker proteins (grey). $MRR^{vGluT2 \rightarrow LHb}$ neurons expressed the mCherry gene that we introduced, but not the EYFP gene that should not be expressed. All mRNA sequencing data have been deposited in NCBI's Gene Expression Omnibus and are accessible through GEO Series accession number GSE300145 (https://www.ncbi.nlm.nih.gov/geo). **(K)** Graph shows significant differences in normalized Pvalb gene expression levels between clusters of $MRR^{vGluT2 \rightarrow LHb}$ neurons. Multiple comparisons with Kruskal-Wallis one-way ANOVA by rank test with posthoc Mann-Whitney U-tests with Bonferroni-corrected p-values ($n_{Type\ gG}$=15 neurons, $n_{Type\ gC}$=13 neurons, $n_{Type\ gH}$=10 neurons, medians and interquartile ranges). Source data are provided as a Source Data file.

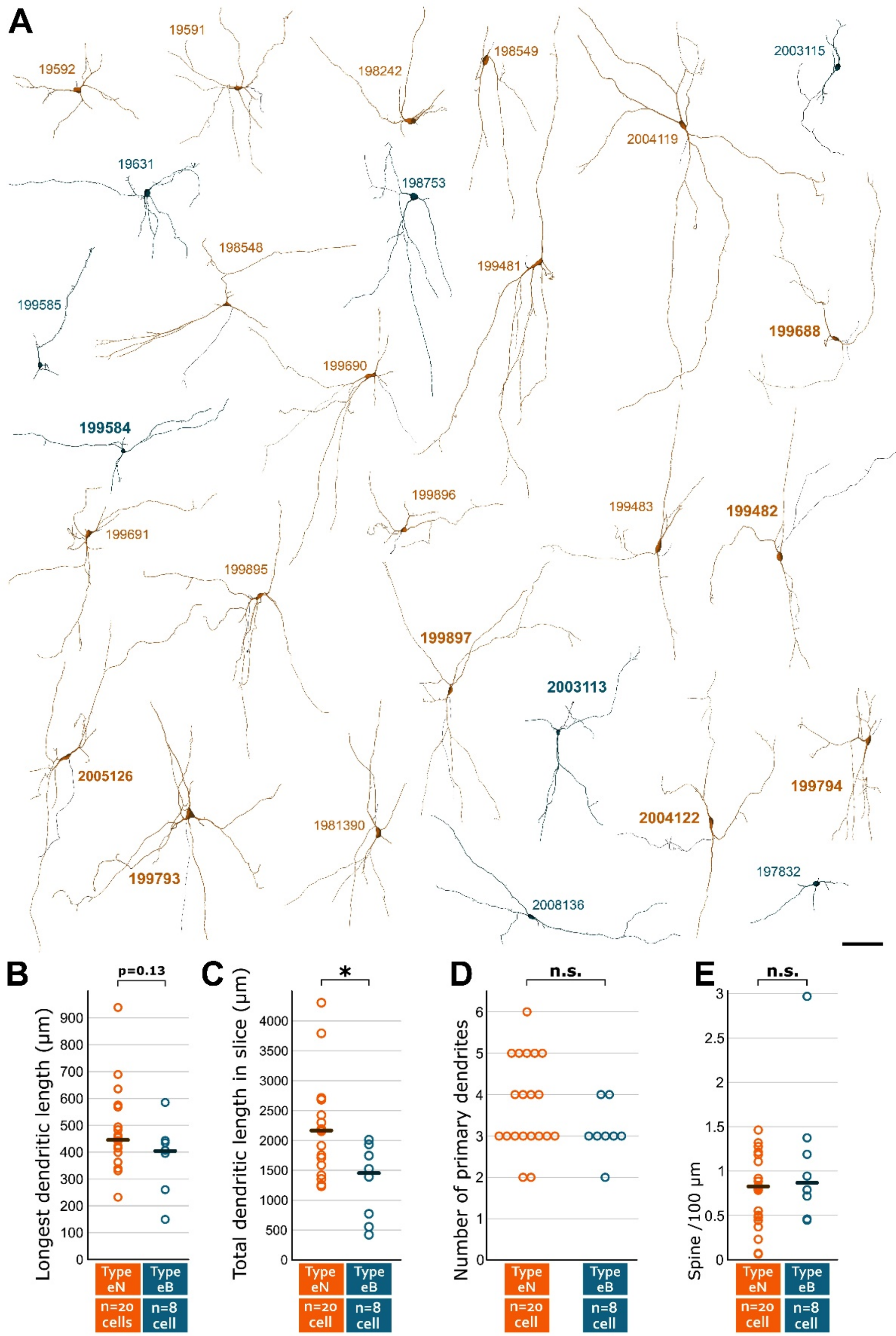

FigS9. Comparison of dendritic arbors of MRR$^{vGluT2\rightarrow LHb}$ neurons.

(A) Three dimensional reconstructions of 28 individual MRR$^{vGluT2\rightarrow LHb}$ neurons from 250 µm thick sections. These MRR$^{vGluT2\rightarrow LHb}$ neurons were recorded in *in vitro* electrophysiological experiments

and filled with biocytin. RNA content of neurons 199688, 199584, 199482, 199897, 2005126, 199793, 2003113, 2004122, 199794 (bold typeface) were also harvested for mRNA sequencing analysis and their data are presented in other figures (Fig5, FigS5-S8). 19592, 19591 and 19631 neurons were recorded and reconstructed from coronal slices (the top of their illustrations are dorsally oriented in the brain). All other neurons were recorded and reconstructed from horizontal slices (the top of their illustrations are rostrally oriented in the brain). Orange neurons belong to the electrophysiologically *Type eN* cluster, whereas blue neurons are *Type eB*, see Fig5, FigS5, FigS6 and Results for details. Scale bar, 100 µm. **(B)** The length of the longest dendrite that could be reconstructed from the 250 µm thick sections ($n_{Type\ eN}$=20 neurons, $n_{Type\ eB}$=8 neurons). **(C)** The total dendritic length per neuron in the 250 µm thick slice ($n_{Type\ eN}$=20 neurons, $n_{Type\ eB}$=8 neurons). **(D)** The number of primary dendrites ($n_{Type\ eN}$=20 neurons, $n_{Type\ eB}$=8 neurons). **(E)** The spine density ($n_{Type\ eN}$=20 neurons, $n_{Type\ eB}$=8 neurons).
Graphs show medians. For statistical details, see TableS3. Source data are provided as a Source Data file.

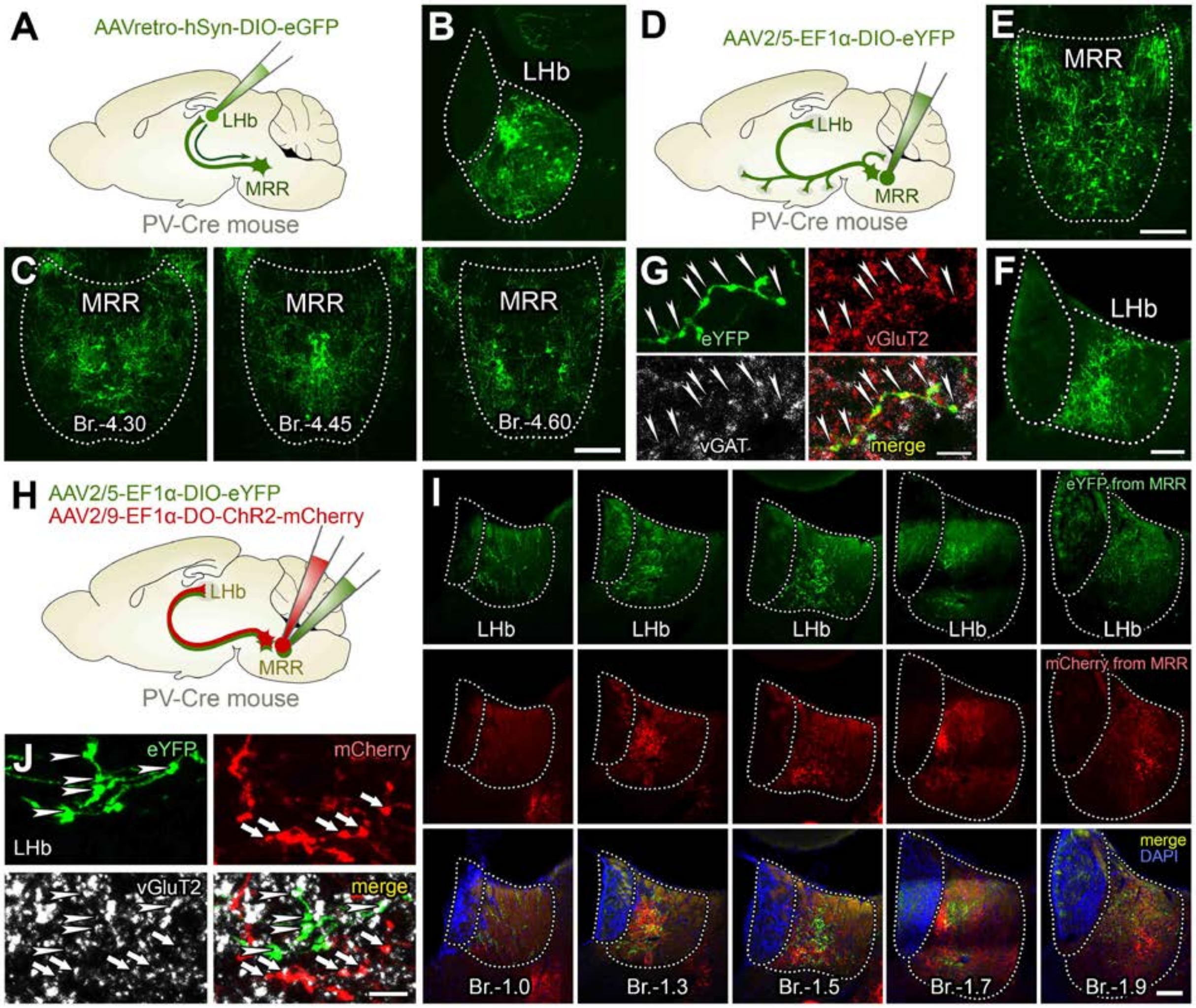

FigS10. Supplementary data for the characterization of MRR^{PV} neurons in mouse.

(A-B) We injected a Cre-dependent, retrogradely spreading, eGFP-encoding AAV into the LHb (B) of two PV-Cre mice bilaterally, which labeled neurons in the MRR (A-C). **(B)** Injection site labeling in the LHb in PV-Cre mouse. **(C)** Images show retrogradely labeled MRR$^{PV\rightarrow LHb}$ neurons (green) in PV-Cre mouse at three different Bregma coordinates (Br. -4.30 mm, -4.45 mm, -4.60 mm). Scale bar for B-C, 200 μm.

(D-E) We injected a Cre-dependent, anterograde, eYFP-encoding AAV into the MRR of two PV-Cre mice **(E)** Injection site labeling in the MRR in PV-Cre mouse. Scale bar, 200 μm. **(F)** eYFP labeling revealed abundant MRR^{PV} innervation of the LHb in PV-Cre mouse that resembles MRR^{vGluT2} neuronal innervation (see FigS3). Scale bar, 100 μm. **(G)** Images and our analyses showed that at least 87.5% (246/281, from 2 mice) of virally labeled MRR PV putative terminals (green, white arrows) were positive for vGluT2 (red), and only 5% (14/281, from 2 mice) were positive for vGAT in the LHb. Scale bar, 5 μm. **(H)** We also investigated the projection pattern of PV-positive and -negative MRR neurons in the LHb. We injected Cre-dependent (Cre-ON), eYFP-

encoding AAV into the MRR of two PV-Cre mice to label axons of MRR^{PV} neurons. To simultaneously label all neurons that do not express PV in these mice, we co-injected Cre-OFF (labeling the PV negative neurons in the MRR directly), mCherry-encoding AAV also (H). **(I)** Fluorescent images show PV-positive axonal fibers (green, eYFP) of $MRR^{vGluT2 \rightarrow LHb}$ neurons and PV-negative axonal fibers (red, mCherry) of $MRR^{vGluT2 \rightarrow LHb}$ neurons in the LHb at five different Bregma coordinates (Br. -1.0 mm, -1.3 mm, -1.5 mm, -1.7 mm, -1.9 mm). Merged images with DAPI labeling of cell nuclei show that eYFP and mCherry fibers innervate similar subregions of the LHb. Scale bar, 100 µm. **(J)** Confocal laser-scanning images showed that both eYFP-positive (reports PV) MRR fibers (green, marked by white arrowheads) and mCherry-positive (reports PV-negative fibers). MRR fibers (red, marked by white arrows) were positive for vGluT2 (white) in the LHb (2 mice). Scale bar, 5 µm.

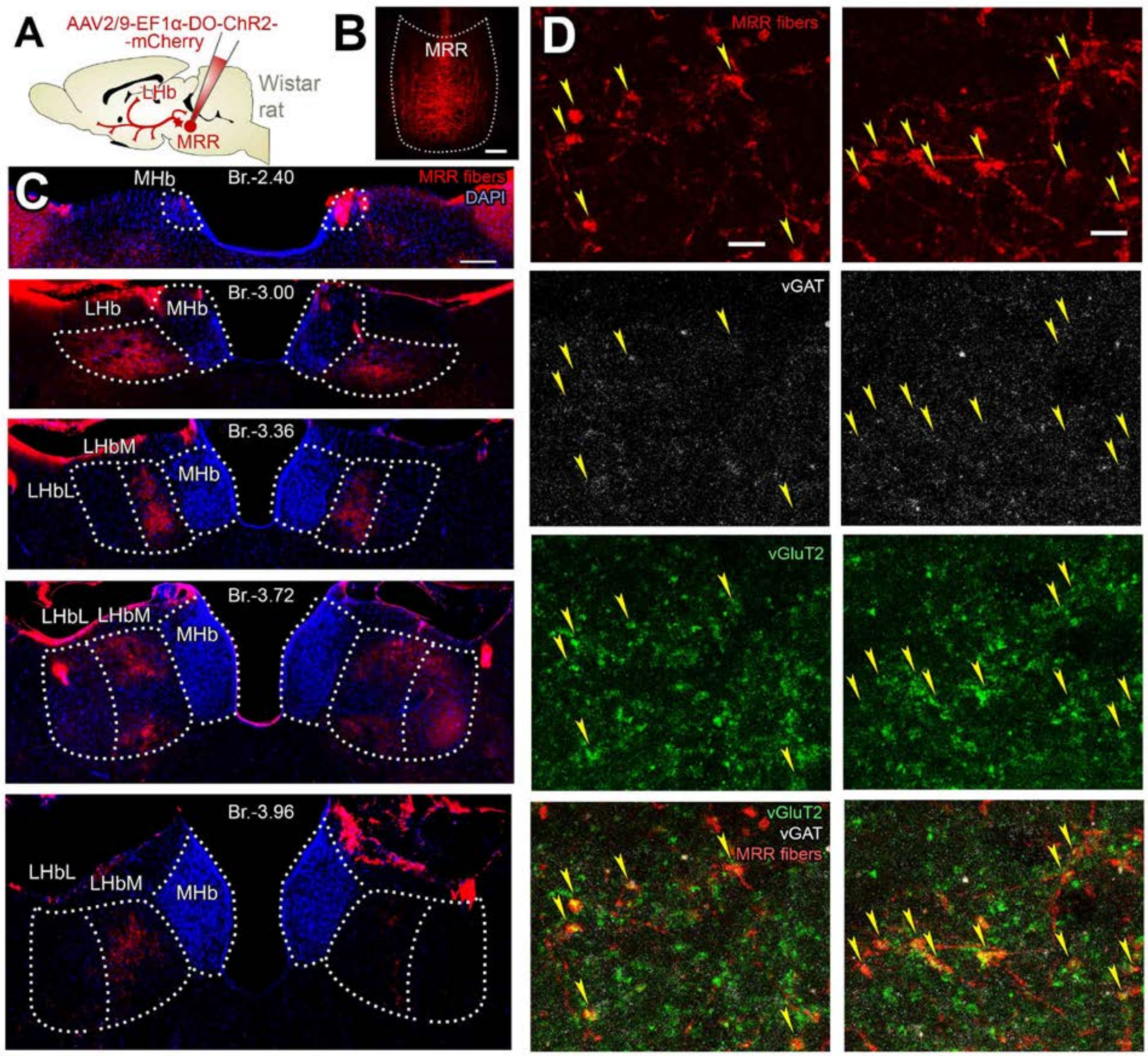

FigS11. Supplementary data for the characterization of MRR$^{vGluT2\rightarrow LHb}$ neurons in rat

(A) MRR neurons were labeled with mCherry using AAV2/9-EF1α-DO-ChR2-mCherry virus in four Wistar rats. **(B)** Fluorescent image shows mCherry expression (red) in the rat MRR, Scale bar, 200 µm. **(C)** mCherry labeling (red) and DAPI staining of cell nuclei (blue) revealed abundant MRR innervation of the LHb (mostly medial part) at multiple Bregma coordinates (Br. -2.40 mm, -3.00 mm, -3.36 mm, -3.72 mm, -3.96 mm), Scale bar, 200 µm. **(D)** Confocal laser scanning images show that MRR axonal fibers (red, yellow arrowheads) were positive for vGluT2 (green), but not for vGAT (white) in the rat LHb. Scale bar, 5 µm. Our measurement showed that at least 71% (124/169) putative MRR terminals in the LHb are vGluT2 positive.

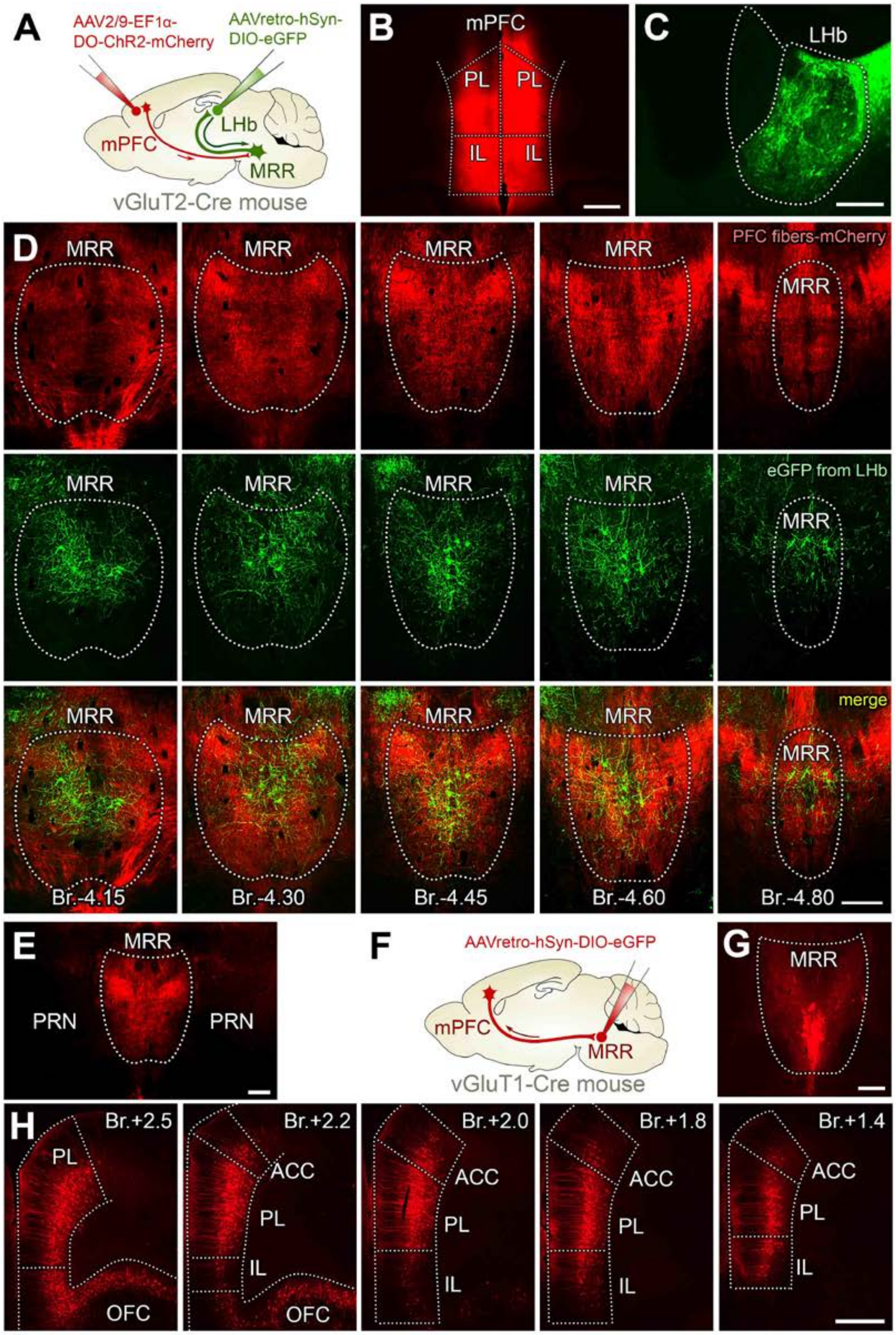

FigS12. Abundant innervation from mPFC targets MRR$^{vGluT2\rightarrow LHb}$ neurons.

(A) Retrograde, bilateral labeling of MRR$^{vGluT2\rightarrow LHb}$ neurons from the LHb was combined with the anterograde, bilateral viral labeling of mPFC$^{\rightarrow MRR}$ neurons in four vGluT2-Cre mice. **(B-C)**

Fluorescent images show mCherry expression (red) in the mPFC, including the prelimbic (PL) and the infralimbic (IL) regions, and eGFP expression (green) in the LHb. Scale bars, 500 μm for the mPFC and 200 μm for the LHb. **(D)** Images show that mPFC axonal fibers (red) densely innervate the entire rostro-caudal extent of the MRR (from Bregma -4.15 mm to -4.80 mm). Scale bar, 200 μm. **(E)** Fluorescent image demonstrates that anterogradely labeled mPFC fibers selectively innervate the MRR, but not the adjacent pontine reticular nucleus (PRN). Scale bar, 200 μm. **(F)** eGFP-encoding, Cre-dependent, retrograde AAV was injected into the MRR to label its cortical inputs in two vGluT1-Cre mice. **(G)** Image shows eGFP expression (red) in the MRR. Scale bar, 200 μm. **(H)** Representative fluorescent images from different Bregma coordinates (from Br. +2.5 mm to Br. +1.4 mm) show the cortical inputs of the MRR (red) including the anterior cingulate cortex (ACC), the prelimbic cortex (PL), the infralimbic cortex (IL) and the orbitofrontal cortex (OFC). Scale bar, 500 μm.

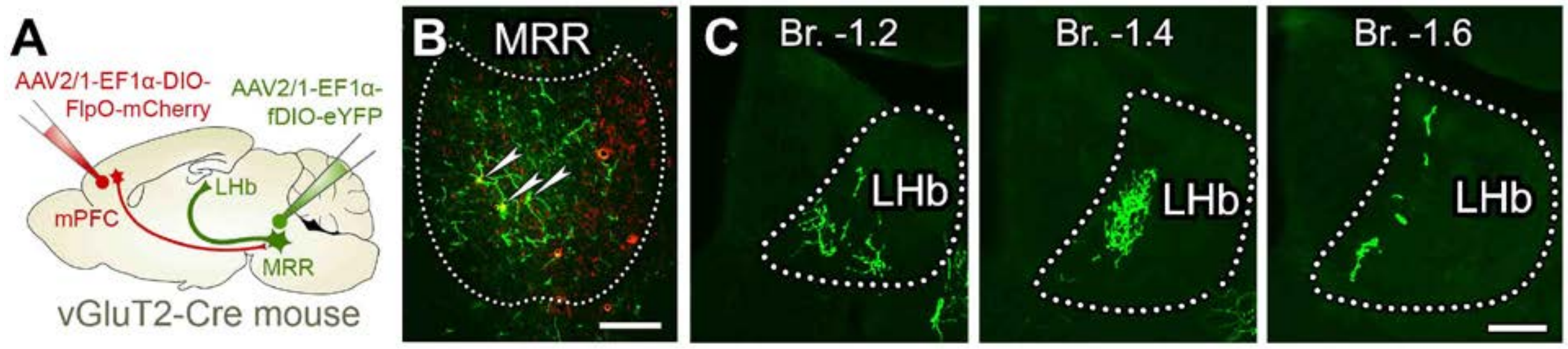

FigS13. mPFC targets LHb-projecting MRR vGluT2 neurons.

(A) Anterograde monotrans-synaptically spreading AAV2/1-EF1α-DIO-FlpO-mCherry virus was injected into mPFC bilaterally, then AAV2/1-EF1α-fDIO-eYFP virus was injected into the MRR of two vGluT2-Cre mice. **(B)** Fluorescent image shows the injection site and monotrans-synaptically labeled MRR^{vGluT2} neurons (yellow neuron, white arrows) expressing mCherry and eYFP simultaneously. Scale bar, 200 µm. **(C)** Representative fluorescent images at three different Bregma coordinates (Br. -1.2 mm, -1.4 mm, -1.6 mm) show axonal fibers of MRR^{vGluT2} neurons (directly targeted by mPFC neurons) in the LHb. Scale bar, 100 µm.

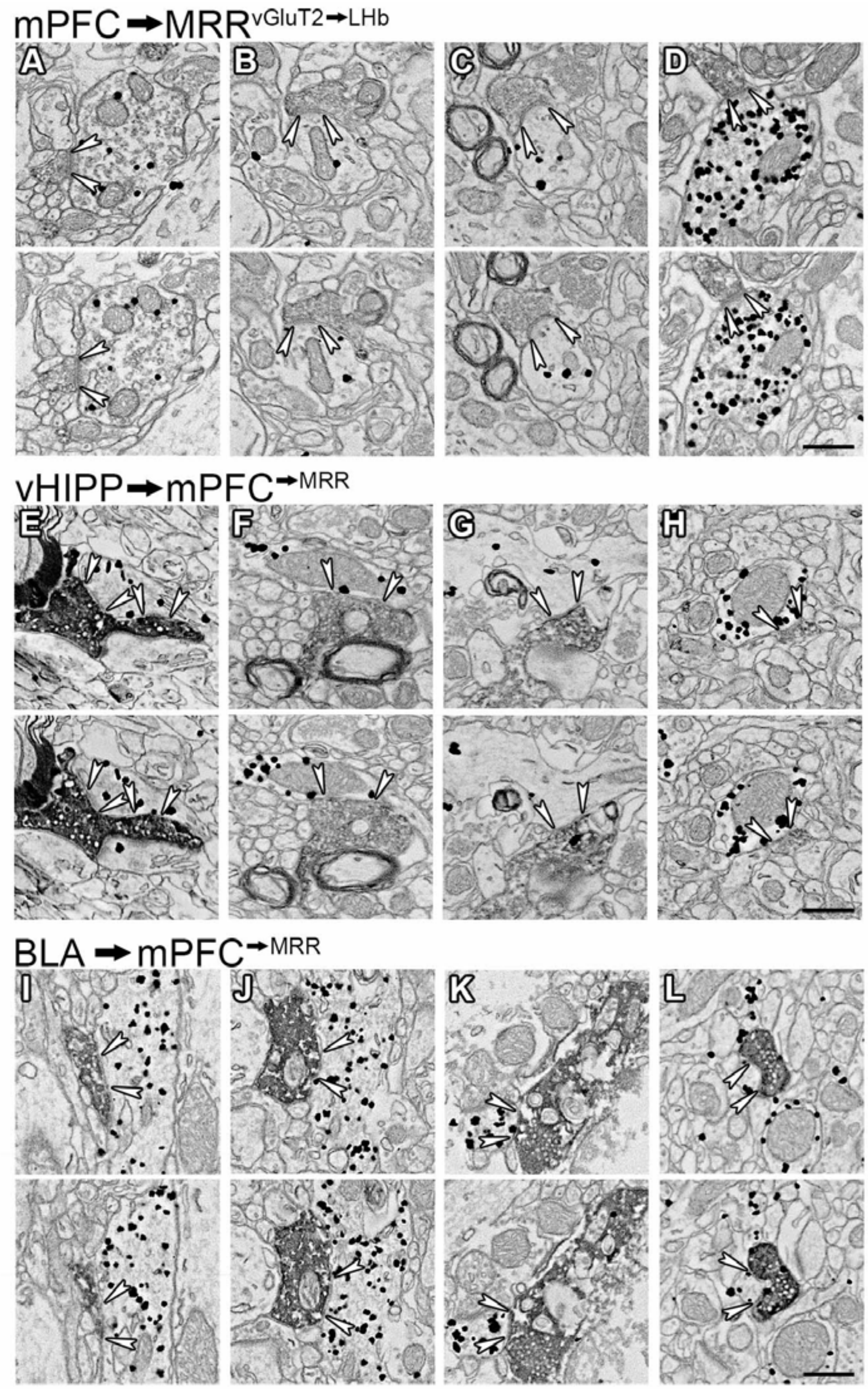

FigS14. Supplementary data for the electron microscopy experiments

(**A-D**) Representative synaptic contacts between axon terminals from the mCherry-positive mPFC neurons (DAB precipitate-labeled) and eGFP-positive dendrites of $MRR^{vGluT2 \rightarrow LHb}$ neurons (labeled with immunogold particles) in the MRR. White arrowheads indicate the edges of the synapses. Experiments detailed on Fig7D-I. **(E-H)** vHIPP axon terminals (DAB precipitate labeling) form synaptic contacts (white arrowheads) on dendritic spine (E) and dendrites (F-H) of

MRR projecting mPFC neurons (labeled with immunogold particles) in the mPFC. Experiments detailed on Fig10A-E. **(I-L)** BLA axon terminals (DAB precipitate labeling) form synaptic contacts (white arrowheads) with dendrites (I-J) and dendritic spines (K-L) of MRR projecting mPFC neurons (labeled with immunogold particles) in the mPFC. Experiments detailed on Fig10F-L. Scale bars, 500 nm.

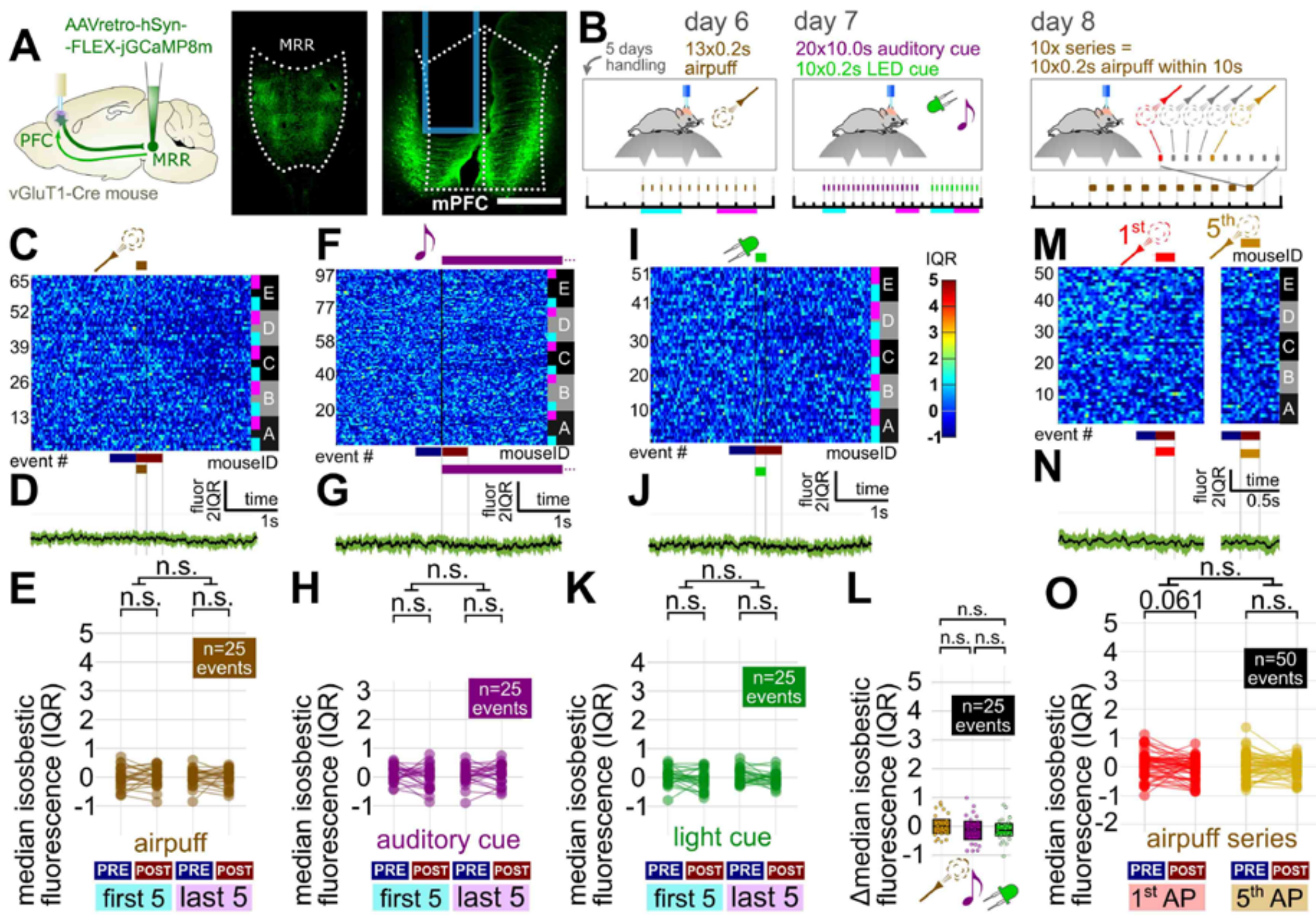

FigS15. Supplementary data for the activity of mPFC$^{\rightarrow MRR}$ neurons - the isosbestic signals.

(A) Cre-dependent, GCaMP8m-encoding, retrograde AAV was injected into the MRR of vGluT1-Cre mice to label mPFC$^{\rightarrow MRR}$ neurons. An optic fiber was implanted over the left mPFC. Fluorescent images show the injection site in the MRR (green). Retrogradely labeled mPFC$^{\rightarrow MRR}$ neurons (green), and the location of the optic fiber (blue) in the mPFC. Scale bar, 500 μm for both images. **(B)** Head-fixed fiber photometry protocol and timeline. Day 6: thirteen 0.2-second-long airpuffs. Day 7: twenty 10.0-second-long auditory tones and ten 0.2-second-long LED light flashes. Day 8: ten airpuff series, each series had ten 0.2-second-long airpuffs within 10 seconds. **(C, F and I)** Heatmap plot shows mPFC$^{\rightarrow MRR}$ isosbestic control signals (detrended, normalized signals) aligned to (C) airpuffs (F) tone cues and (I) LED light flashes, respectively (n_{events}=65, 97, 51 respectively, 5 mice). Fiberphotometry units are normalized to the interquartile range (IQR, see methods). **(D, G and J)** Peri-event plots show medians and interquartile ranges of mPFC$^{\rightarrow MRR}$ isosbestic control signals aligned to each stimulus respectively. **(E, H and K)** Plots show a comparison of mPFC$^{\rightarrow MRR}$ isosbestic control signals before (0.5s) and after (0.5s) the start of the stimuli. Data are calculated for the first and last 5 stimuli. Differences in the amount of fluorescence changes are also compared between the first 5 and the last 5 stimuli (n_{events}=25, 5 mice). **(L)** Graph shows that mPFC$^{\rightarrow MRR}$ isosbestic control signal does not follow presumed stimulus aversiveness (n_{events}=25 from 5 mice). **(M)** Heatmap plots show no adaptation of mPFC$^{\rightarrow MRR}$ isosbestic control signals to the same airpuffs within the first 5 seconds of the series

(n_{events}=50, 5 mice) **(N)** Perievent plots show medians and interquartile ranges of mPFC$^{\rightarrow MRR}$ isosbestic control signals aligned to the first (left) and the fifth (right) airpuffs in each airpuff series. **(O)** mPFC$^{\rightarrow MRR}$ isosbestic control signals (0.2s before, 0.2s after airpuff) is not significantly different in the first (left) than in the fifth (right) airpuffs in each series, these were delivered within 5 seconds (n_{events}=50, 5 mice).

Graphs show medians and interquartile ranges. For statistical details, see TableS2. For reconstructed optic fiber localization see FigS16. Source data are provided as a Source Data file.

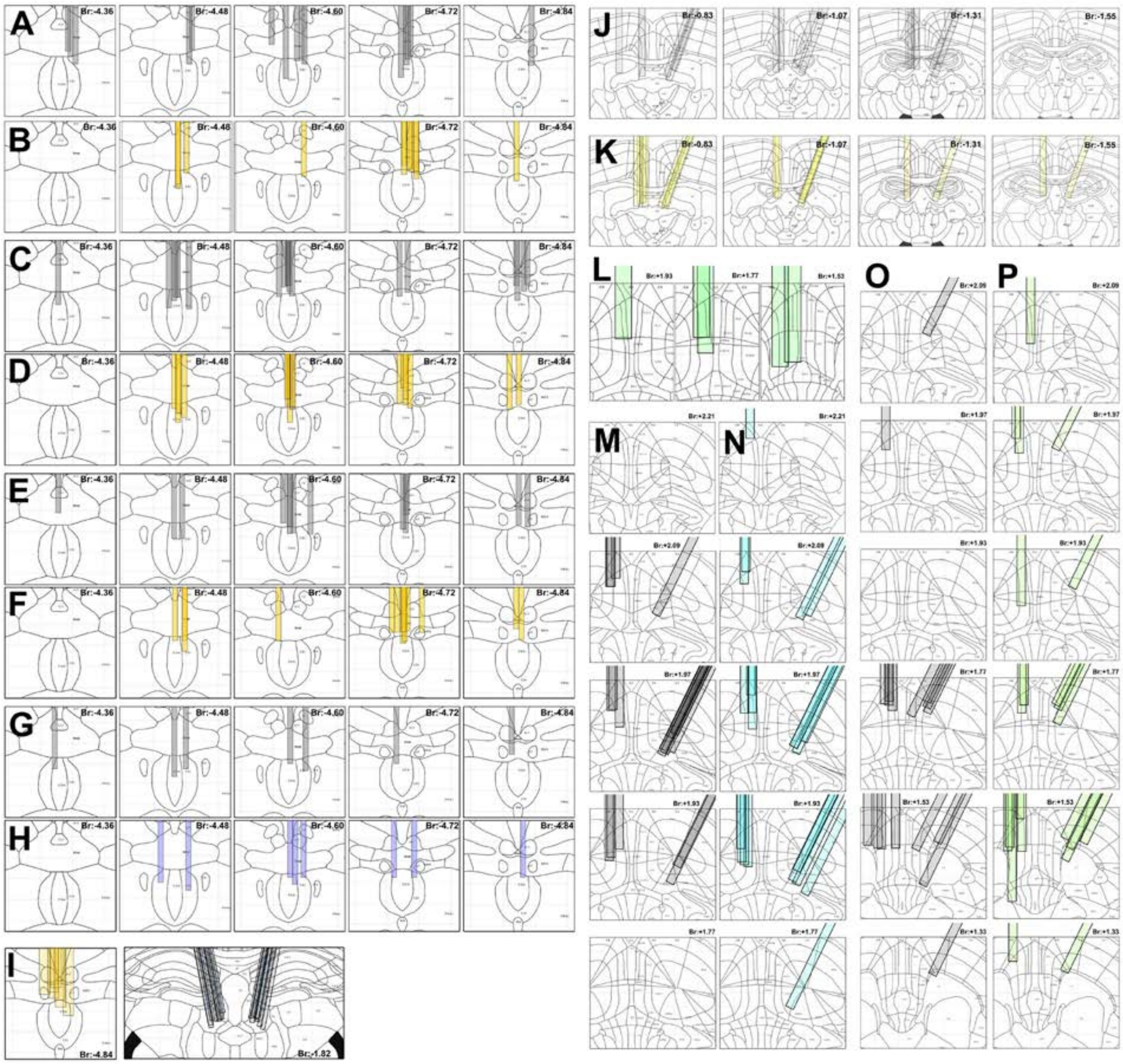

FigS16. Optic fiber localizations in mice used in behavioral, fiber photometry and *in vivo* electrophysiological recording experiments

Summary of optic fiber location in every mouse used in electrophysiological, behavioral optogenetic and fiber photometry recording experiments. Viral labeling locations were verified in all mice from all experiments. Here we illustrated the exact location of the optic fiber tips and overlayed these illustrations on top of each other to get a representative localization within the stereological atlas [76]. **(A, B)** MRR^{vGluT2} ArchT contextual fear conditioning experiments. (grey (A) CTRL mice, yellow (B) ArchT mice, described in Fig1A-D and FigS1A) **(C, D)** MRR^{vGluT2} ArchT cued fear conditioning experiments. (grey (C) CTRL mice, yellow (D) ArchT mice, described in Fig1A-B, E-K and FigS1D) **(E, F)** MRR^{vGluT2} ArchT cued-contextual fear conditioning experiments. (grey (E) CTRL mice, yellow (F) ArchT mice, described in Fig1A-B, L-P and FigS1E) **(G, H)** MRR ChR2 cued place aversion experiments. (grey (G) CTRL mice, blue (H)

ChR2 mice, described in FigS1F-I) **(I)** $MRR^{vGluT2 \rightarrow LHb}$ ArchT cued fear conditioning combined with *in vivo* electrophysiology experiments. Left optic fiber locations, right probe locations. Described in Fig3A-L **(J, K)** $MRR^{vGluT2 \rightarrow LHb}$ ArchT axon optogenetics in the LHb contextual fear conditioning experiment. (grey (J) CTRL mice, yellow (K) ArchT mice, described in Fig3N-R). **(L)** $mPFC^{\rightarrow MRR}$ fiber photometry in the mPFC. Described in Fig8 and FigS15 **(M, N)** $mPFC^{\rightarrow MRR}$ ChR2 optogenetics in the mPFC place aversion experiment. (grey (M) CTRL mice, blue (N) ChR2 mice, described in Fig7J-N). **(O, P)** $mPFC^{\rightarrow MRR}$ ArchT optogenetics in the mPFC contextual fear conditioning experiment. (grey (O) CTRL mice, green (P) ArchT mice, described in Fig9A-E). Atlas reference panels were generated from the Stereotaxic Topographic Atlas of the Mouse Brain (STAM; Feng et al., 2025) and adapted under the Creative Commons Attribution 4.0 International License.

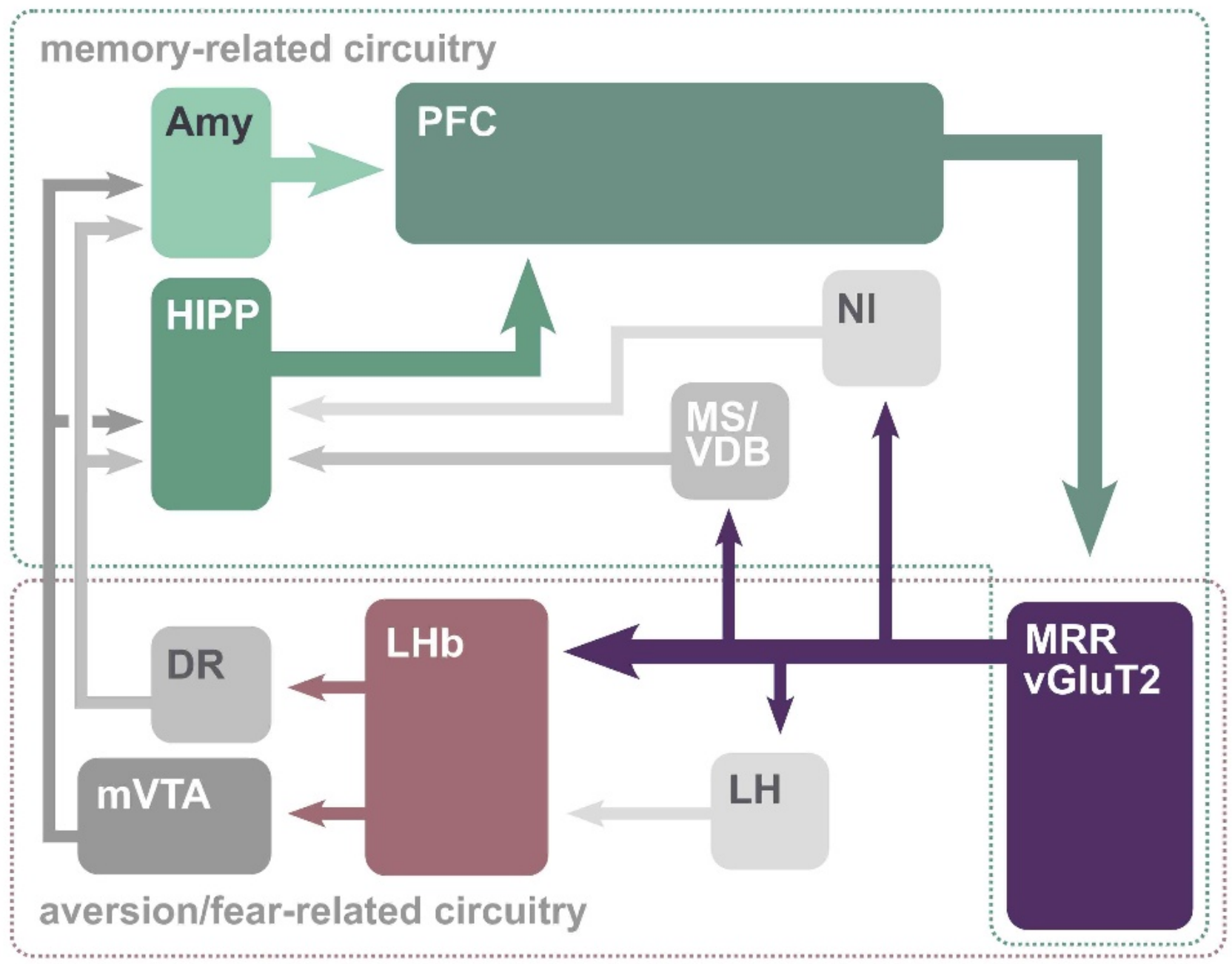

FigS17. Highly simplified illustration of the anatomical connections of MRR^{vGluT2} neurons in the fear memory recall circuit.

The canonical tripartite memory circuit (Amy, HIPP and PFC) directly targets the MRR^{vGluT2} neurons that in turn can update both the aversion/fear-related negative valence centers and memory-related neural populations.

Abbreviations: Amy: amygdala, HIPP: hippocampal formation, PFC: prefrontal cortex, NI: nucleus incertus, MS/VDB: medial septum – vertical limb of the diagonal band of Broca complex, vGluT2: vesicular glutamate transporter 2, MRR: median raphe region, LH: lateral hypothalamus, LHb: lateral habenula, DR: dorsal raphe, mVTA: medial part of the ventral tegmental area

TableS1. Statistical details of behavioral experiments

Figure	experimental condition	group	n mice	quartile 1	median	quartile 3	statistical test and p-value
FigS1A	day 6, baseline (freezing time %)	CTRL	15	0.00	0.00	0.00	Mann-Whitney U-test: CTRL vs. ArchT p=0.9188
		ArchT	11	0.00	0.00	0.00	
	day 6, shocks (freezing time %)	CTRL	15	6.60	14.60	20.46	Mann-Whitney U-test: CTRL vs. ArchT p=0.2372
		ArchT	11	5.33	7.93	13.03	
	day 6, whole 6min trial (freezing time %)	CTRL	15	2.64	5.85	8.19	Mann-Whitney U-test: CTRL vs. ArchT p=0.0973
		ArchT	11	0.83	3.00	5.21	
FigS 1C	day 6, baseline locomotion (head speed mm/s)	CTRL	15	-18.17	-10.93	-3.14	Mann-Whitney U-test: CTRL vs. ArchT p=0.2812
		ArchT	11	-20.74	-1.66	9.89	
Fig1D	day 7, 8min (freezing time %)	CTRL	15	16.33	23.44	32.49	Mann-Whitney U-test: CTRL vs. ArchT: **: p=0.0020
		ArchT	11	3.46	8.19	19.47	
	day 8, 8min (freezing time %)	CTRL	15	15.92	18.17	33.09	Mann-Whitney U-test: CTRL vs. ArchT: *: p=0.0473
		ArchT	11	3.42	10.00	18.46	
	day 9, 5min (freezing time %)	CTRL	15	12.36	22.44	35.85	Mann-Whitney U-test: CTRL vs. ArchT: **: p=0.0092
		ArchT	11	4.11	8.85	17.17	
FigS1D	day 6, baseline (freezing time %)	CTRL	21	0.00	0.00	0.00	Mann-Whitney U-test: CTRL vs. ArchT p=0.8204
		ArchT	16	0.00	0.00	0.00	
	day 6, shocks (freezing time %)	CTRL	21	12.93	15.96	19.16	Mann-Whitney U-test: CTRL vs. ArchT p=0.9879
		ArchT	16	11.87	14.96	20.69	
	day 6, whole 6min trial (freezing time %)	CTRL	21	6.47	7.98	9.67	Mann-Whitney U-test: CTRL vs. ArchT p=0.9879
		ArchT	16	5.93	7.48	10.34	
Fig1F	day 7, baseline, 3min (freezing time %)	CTRL	21	0.00	2.15	3.96	Mann-Whitney U-test: CTRL vs. ArchT p=0.6386
		ArchT	16	0.00	0.00	4.54	
	day 7, 1. cue (freezing time %)	CTRL	21	27.73	43.47	63.73	Mann-Whitney U-test: CTRL vs. ArchT p=0.8916
		ArchT	16	30.80	48.53	59.73	
	day 7, 2. cue (freezing time %)	CTRL	21	22.93	35.73	46.67	Mann-Whitney U-test: CTRL vs. ArchT p=0.8916
		ArchT	16	17.20	28.53	61.47	
	day 7, 3. cue (freezing time %)	CTRL	21	13.33	39.20	58.40	Mann-Whitney U-test: CTRL vs. ArchT p=0.1226
		ArchT	16	2.40	13.33	40.93	
	day 7, 4. cue (freezing time %)	CTRL	21	19.47	32.27	40.53	Mann-Whitney U-test: CTRL vs. ArchT *: p=0.0471
		ArchT	16	0.00	11.07	33.47	
	day 7, 5. cue (freezing time %)	CTRL	21	11.20	27.47	45.33	Mann-Whitney U-test: CTRL vs. ArchT *: p=0.0112
		ArchT	16	0.00	9.33	19.47	
Fig1G	day 8, baseline, 3min (freezing time %)	CTRL	21	0.00	3.51	5.00	Mann-Whitney U-test: CTRL vs. ArchT *: p=0.0135
		ArchT	16	0.00	0.00	1.69	
	day 8, 1. cue (freezing time %)	CTRL	21	36.53	45.87	58.40	Mann-Whitney U-test: CTRL vs. ArchT ***: p=0.0007
		ArchT	16	5.33	25.60	37.87	
	day 8, 2. cue (freezing time %)	CTRL	21	23.73	38.40	58.67	Mann-Whitney U-test: CTRL vs. ArchT **: p=0.0076
		ArchT	16	7.20	26.00	31.07	
	day 8, 3. cue (freezing time %)	CTRL	21	16.80	32.80	43.47	Mann-Whitney U-test: CTRL vs. ArchT **: p=0.0015
		ArchT	16	0.00	8.67	22.93	
	day 8, 4. cue (freezing time %)	CTRL	21	10.67	30.67	42.67	Mann-Whitney U-test: CTRL vs. ArchT *: p=0.0229
		ArchT	16	0.00	6.80	26.53	
	day 8, 5. cue (freezing time %)	CTRL	21	5.07	15.47	34.40	Mann-Whitney U-test: CTRL vs. ArchT *: p=0.0229
		ArchT	16	0.00	4.53	17.33	
Fig1I	day 9, context "B" baseline (freezing time %)	CTRL	21	0.00	0.67	5.00	Mann-Whitney U-test: CTRL vs. ArchT p=0.3385
		ArchT	16	0.00	0.31	1.56	
	day 9, context "B" cues (freezing time %)	CTRL	21	17.87	26.13	36.98	Mann-Whitney U-test: CTRL vs. ArchT *: p=0.0471
		ArchT	16	11.20	20.04	24.18	

continued on next page…

Figure	experimental condition	group	n mice	quartile 1	median	quartile 3	statistical test and p-value
**Fig1 K**	day 9, context "A" (freezing %, trial duration: 5 mins)	CTRL	21	9.68	20.27	25.92	Mann-Whitney U-test: CTRL vs. ArchT: ***: p=0.0003
		ArchT	16	1.12	3.49	9.01	
**FigS1E**	day 6, baseline (freezing time %)	CTRL	13	0.00	0.00	0.00	Mann-Whitney U-test: CTRL vs. ArchT p=0.7241
		ArchT	13	0.00	0.00	0.62	
	day 6, shocks (freezing time %)	CTRL	13	7.46	13.64	19.10	Mann-Whitney U-test: CTRL vs. ArchT p=0.4184
		ArchT	13	8.93	15.50	22.48	
	day 6, whole 6min trial (freezing time %)	CTRL	13	3.73	6.82	9.56	Mann-Whitney U-test: CTRL vs. ArchT p=0.336
		ArchT	13	4.47	8.76	12.44	
**Fig1M**	day 7, baseline, 3min (freezing time %)	CTRL	13	14.44	23.78	27.16	Mann-Whitney U-test: CTRL vs. ArchT p=0.1857
		ArchT	13	9.01	14.93	17.78	
	day 7, 1. cue (freezing time %)	CTRL	13	30.40	49.07	50.40	Mann-Whitney U-test: CTRL vs. ArchT ***p=0.0004
		ArchT	13	4.27	15.47	26.13	
	day 7, 2. cue (freezing time %)	CTRL	13	28.53	42.67	63.20	Mann-Whitney U-test: CTRL vs. ArchT **p=0.0029
		ArchT	13	4.53	13.60	27.20	
	day 7, 3. cue (freezing time %)	CTRL	13	21.33	38.40	48.27	Mann-Whitney U-test: CTRL vs. ArchT **p=0.0051
		ArchT	13	0.00	12.00	25.60	
	day 7, 4. cue (freezing time %)	CTRL	13	6.67	31.47	57.33	Mann-Whitney U-test: CTRL vs. ArchT *: p=0.0191
		ArchT	13	0.00	9.07	9.87	
	day 7, 5. cue (freezing time %)	CTRL	13	9.87	22.40	36.27	Mann-Whitney U-test: CTRL vs. ArchT p=0.1014
		ArchT	13	0.00	8.80	18.93	
**Fig1N**	day 8, baseline, 3min (freezing time %)	CTRL	13	1.64	3.76	14.53	Mann-Whitney U-test: CTRL vs. ArchT *: p=0.0191
		ArchT	13	0.00	0.62	3.76	
	day 8, 1. cue (freezing time %)	CTRL	13	20.00	42.67	53.87	Mann-Whitney U-test: CTRL vs. ArchT *: p=0.0164
		ArchT	13	0.00	9.33	29.33	
	day 8, 2. cue (freezing time %)	CTRL	13	18.40	30.13	52.80	Mann-Whitney U-test: CTRL vs. ArchT **: p=0.0035
		ArchT	13	0.00	7.47	13.60	
	day 8, 3. cue (freezing time %)	CTRL	13	5.07	33.60	41.60	Mann-Whitney U-test: CTRL vs. ArchT *: p=0.0191
		ArchT	13	0.00	5.87	11.47	
	day 8, 4. cue (freezing time %)	CTRL	13	8.80	24.80	41.60	Mann-Whitney U-test: CTRL vs. ArchT **: p=0.0019
		ArchT	13	0.00	0.00	4.27	
	day 8, 5. cue (freezing time %)	CTRL	13	14.93	34.13	45.60	Mann-Whitney U-test: CTRL vs. ArchT ***: p=0.0008
		ArchT	13	0.00	4.27	5.60	
**Fig1P**	day 9, context "A" baseline (freezing time %)	CTRL	13	3.42	4.44	8.18	Mann-Whitney U-test: CTRL vs. ArchT *: p=0.0222
		ArchT	13	0.00	0.89	3.91	
	day 9, context "A" cues (freezing time %)	CTRL	13	18.76	28.09	48.36	Mann-Whitney U-test: CTRL vs. ArchT **: p=0.0012
		ArchT	13	4.44	9.16	13.96	
	day 9, context "B" baseline (freezing %)	CTRL	7	0.67	0.80	5.00	Mann-Whitney U-test: CTRL vs. ArchT p=0.3434
		ArchT	5	0.00	0.00	1.56	
	day 9, context "B" cues (freezing %)	CTRL	7	20.80	37.69	50.84	Mann-Whitney U-test: CTRL vs. ArchT p=0.4318
		ArchT	5	13.87	24.09	37.69	
**FigS1I**	day 6, hab (striped time %)	CTRL	7	52.01	54.60	64.41	Wilcoxon Matched Pairs Test: CTRL hab vs. test p=0.6121
	day 8, test (striped time %)	CTRL	7	49.42	57.84	71.72	
	day 6, hab (striped time %)	ChR2	8	62.06	63.25	65.89	Wilcoxon Matched Pairs Test: ChR2 hab vs. test *: p=0.0357
	day 8, test (striped time %)	ChR2	8	52.37	55.68	60.55	

continued on next page…

Figure	experimental condition	group	n mice	quartile 1	median	quartile 3	statistical test and p-value
**Fig3R**	day 7, 8min (freezing time %)	CTRL	7	23.80	32.60	38.95	Mann-Whitney U-test: CTRL vs. ArchT p=0.0721
		ArchT	8	10.50	17.85	23.40	
	day 8, 8min (freezing time %)	CTRL	7	14.35	27.70	33.25	Mann-Whitney U-test: CTRL vs. ArchT p=0.1206
		ArchT	8	8.58	12.85	16.58	
	day 9, 5min (freezing time %)	CTRL	7	15.95	21.50	31.55	Mann-Whitney U-test: CTRL vs. ArchT: *: p=0.0401
		ArchT	8	5.38	10.25	19.18	
**Fig4D**	MRRvGluT2 activity in corticosterone stress model (c-Fos+ | EGFP+ %, 3 sections from each mouse)	CTRL	2	11.10	13.85	16.14	Mann-Whitney U-test: CTRL vs. CORT: *: p=0.0496 CTRL vs. KET: p=0.4848 KET vs. CORT: *: p=0.0120
		CORT	3	14.85	19.30	22.22	
		KET	2	8.56	10.33	13.57	
**FigS4B**	SPT in corticosterone stress model (sucrose preference %)	CTRL	7	91.00	93.33	93.94	Mann-Whitney U-test: CTRL vs. CORT: *: p=0.0140 CTRL vs. KET: p=0.9551 KET vs. CORT: p=0.1079
		CORT	6	37.07	66.03	86.08	
		KET	8	74.33	92.80	97.55	
**FigS4L**	day 6, baseline (freezing time %)	CTRL	7	0.00	0.00	0.00	Mann-Whitney U-test: CTRL vs. ArchT p=1.0000
		ArchT	8	0.00	0.00	0.00	
	day 6, shocks (freezing time %)	CTRL	7	8.40	11.50	18.05	Mann-Whitney U-test: CTRL vs. ArchT p=0.1520
		ArchT	8	5.50	6.45	11.20	
**FigS4Q**	day 6, baseline (freezing time %)	CTRL	9	0.00	0.00	0.00	Mann-Whitney U-test: CTRL vs. ArchT p=0.9408
		ArchT	11	0.00	0.00	0.00	
	day 6, shocks (freezing time %)	CTRL	9	11.22	25.46	29.32	Mann-Whitney U-test: CTRL vs. ArchT p=0.4561
		ArchT	11	8.17	16.32	28.85	
**Fig4H**	UMS, baseline (freezing %)	CTRL	12	0.00	0.00	1.00	Mann-Whitney U-test: CTRL vs. FS: p=0.3451 CTRL vs. hM4Di: *: p=0.0148 FS vs. hM4Di: p=0.3054
		FS	9	0.00	0.00	5.91	
		hM4Di	14	0.00	2.09	17.02	
	UMS, shocks (freezing %)	CTRL	12	0.82	1.50	2.88	Mann-Whitney U-test: CTRL vs. FS: ****: p=0.00005 CTRL vs. hM4Di: ****: p=0.0000002 FS vs. hM4Di: p=0.9754
		FS	9	32.25	33.06	53.59	
		hM4Di	14	26.39	42.24	53.73	
**Fig4I**	SPT after UMS protocol (sucrose preference %)	CTRL	12	88.29	91.51	93.70	Mann-Whitney U-test: CTRL vs. FS: *: p=0.0278 CTRL vs. hM4Di: p=0.3744 FS vs. hM4Di: *: p=0.0456
		FS	9	78.72	87.18	87.76	
		hM4Di	14	82.98	90.19	92.75	
**FigS4F**	SPT after CNO injections (sucrose preference %)	CTRL	7	80.80	87.47	92.51	Mann-Whitney U-test: CTRL vs. hM3Dq *: p=0.0349
		hM3Dq	6	76.65	80.41	81.13	
**FigS4G**	TST after CNO injections (mobility %)	CTRL	7	39.30	41.40	42.70	Mann-Whitney U-test: CTRL vs. hM3Dq *: p=0.0349
		hM3Dq	6	29.00	33.50	39.20	
**Fig7N**	day 6, hab (illum time %)	CTRL	9	41.63	51.21	58.13	Wilcoxon Matched Pairs Test: CTRL hab vs. test p=0.1641
	day 7, RTPA (illum time %)	CTRL	9	35.78	41.83	52.49	
	day 6, hab (illum time %)	ChR2	11	41.62	52.58	62.45	Wilcoxon Matched Pairs Test: ChR2 hab vs. test *: p=0.0322
	day 8, RTPA (illum time %)	ChR2	11	17.66	27.35	55.38	

continued on next page…

**Fig9E**	day 7, 8min (freezing time %)	CTRL	9	23.24	26.02	45.73	Mann-Whitney U-test: CTRL vs. ArchT p=0.7664
		ArchT	11	13.99	35.68	47.47	
	day 8, 8min (freezing time %)	CTRL	9	27.55	31.36	35.80	Mann-Whitney U-test: CTRL vs. ArchT **: p=0.0097
		ArchT	11	9.09	17.05	23.25	
	day 9, 5min (freezing time %)	CTRL	9	30.26	32.16	43.50	Mann-Whitney U-test: CTRL vs. ArchT: *: p=0.0465
		ArchT	11	14.36	20.49	28.68	
**Fig9J**	mPFC$^{\rightarrow MRR}$ reactivation (c-Fos+ | eGFP+ %)	home	5	4.89	5.00	6.62	Mann-Whitney U-test: home vs. ctxA *: p=0.0159
		ctxA	4	23.96	24.94	29.33	

TableS2. Statistical details of fiberphotometry experiments

figure	channel	day #	day name	event ID	quant	diff or val	unit	t windows (s)	n mice	n stat	quartile 1	median	quartile 3	statistical test and p-value
FigS2B, Fig2C	Isosbestic	4	habituation	T1	AUC	post	z-score	30	6	6	-6.31320	-2.14356	1.82647	Wilcoxon Matched Pairs Test:<br>conditioning T1 vs. conditioning T5: p=0.46<br>conditioning T1 vs. recall T1: p=0.46<br>conditioning T1 vs. recall T5: p=0.20<br>recall T1 vs. recall T5: p=0.84<br><br>Regression:<br>habituation: p=0.66<br>conditioning: p=0.90<br>recall: p=0.10
				T2				30	6	6	-16.08676	-6.97284	-2.07094	
				T3				30	6	6	-11.13792	1.31538	12.67034	
				T4				30	6	6	-11.54515	-1.59175	-1.06259	
				T5				30	6	6	-33.06369	-0.81522	10.50809	
		5	conditioning	T1				30	8	8	-5.24302	-0.36651	3.92619	
				T2				30	8	8	-2.86353	5.39166	12.52230	
				T3				30	8	8	0.11327	1.74006	4.62686	
				T4				30	8	8	-0.38053	4.31179	8.39079	
				T5				30	8	8	-12.87794	0.34995	16.59306	
		6	recall	T1				30	8	8	-6.43409	0.17848	15.20405	
				T2				30	8	8	-17.00286	-3.45755	-2.67228	
				T3				30	8	8	-20.84865	-9.84951	-5.68535	
				T4				30	8	8	-22.78835	-9.20806	3.45030	
				T5				30	8	8	-2.20067	1.40032	4.60939	
	Ca2+ dependent	4	habituation	T1				30	6	6	-36.65158	-16.73821	43.98748	Wilcoxon Matched Pairs Test:<br>conditioning T1 vs. conditioning T5: p=0.0781<br>conditioning T1 vs. recall T1: **: p=0.0078<br>conditioning T1 vs. recall T5: **: p=0.0078<br>recall T1 vs. recall T5: *: p=0.04995<br><br>Regression:<br>habituation: p=0.50<br>conditioning: p=0.49<br>recall: *: p=0.028
				T2				30	6	6	-19.93846	-8.82392	26.34797	
				T3				30	6	6	-63.89489	-12.78323	5.78613	
				T4				30	6	6	-19.36707	-10.30306	6.82875	
				T5				30	6	6	-22.03506	12.58886	47.06818	
		5	conditioning	T1				30	8	8	-35.24078	-8.92658	1.74081	
				T2				30	8	8	-12.51643	22.75440	44.83384	
				T3				30	8	8	-23.88029	14.56358	31.30450	
				T4				30	8	8	-40.62131	25.70260	38.57851	
				T5				30	8	8	11.04063	19.42710	59.49253	
		6	recall	T1				30	8	8	39.44828	87.67993	110.26069	
				T2				30	8	8	-10.46264	20.18579	60.92327	
				T3				30	8	8	-1.67634	15.94774	30.33560	
				T4				30	8	8	-2.36115	11.20430	31.10155	
				T5				30	8	8	13.90133	23.39376	57.45679	
FigS2C	freezing	6	recall	T1, T2, T3, T4, T5	-	cue-baseline	% (s/s)	30	8	8	-	-	-	Regression:<br>recall: *: p=0.046
FigS15E, Fig8E	Isosbestic	6	airpuff	1st5	median	pre	IQR	0.5	5	25	-0.27230	-0.01787	0.26511	Wilcoxon Matched Pairs Test:<br>1st5 pre vs. 1st5 post: p=0.8949<br>last5 pre vs. last5 post: p=0.5782<br>Mann-Whitney U-test:<br>1st5 post-pre vs. last5 post-pre: p=0.7124
				1st5	median	post	IQR		5	25	-0.20825	0.01400	0.28246	
				1st5	median	post-pre	IQR		5	25	-0.24888	0.00187	0.23706	
				last5	median	pre	IQR		5	25	-0.13357	0.02734	0.19960	
				last5	median	post	IQR		5	25	-0.20026	0.00773	0.22370	
				last5	median	post-pre	IQR		5	25	-0.22807	-0.08278	0.22475	
	Ca2+			1st5	median	pre	IQR	0.5	5	25	-0.64382	-0.28719	0.17535	Wilcoxon Matched Pairs Test:<br>1st5 pre vs. 1st5 post: ****: p=2.980e-7<br>last5 pre vs. last5 post: ****: p=1.192e-7<br>Mann-Whitney U-test:<br>1st5 post-pre vs. last5 post-pre: *: p=0.0161
				1st5	median	post	IQR		5	25	2.23702	2.88536	3.58436	
				1st5	median	post-pre	IQR		5	25	1.97567	3.09827	4.14886	
				last5	median	pre	IQR		5	25	-0.51725	-0.22157	0.09020	
				last5	median	post	IQR		5	25	1.31627	1.76257	2.36442	
				last5	median	post-pre	IQR		5	25	1.05189	2.11738	2.69696	

continued on next page…

Figure	channel	day #	day name	event ID	quant	diff or val	unit	t windows (s)	n mice	n stat	quartile 1	median	quartile 3	statistical test and p-value
**FigS15K, Fig8K**	Isosbestic	7	LED light cue	1st5	median	pre	IQR	0.5	5	25	-0.10863	0.08556	0.18113	Wilcoxon Matched Pairs Test: 1st5 pre vs. 1st5 post: p=0.1135 last5 pre vs. last5 post: p=0.1199 Mann-Whitney U-test: 1st5 post-pre vs. last5 post-pre: p=0.7710
				1st5	median	post	IQR		5	25	-0.28837	-0.08867	0.14053	
				1st5	median	post-pre	IQR		5	25	-0.33401	-0.13710	0.08992	
				last5	median	pre	IQR		5	25	-0.17670	0.05825	0.25729	
				last5	median	post	IQR		5	25	-0.21108	-0.05848	0.12922	
				last5	median	post-pre	IQR		5	25	-0.31443	-0.20298	0.13460	
	Ca2+			1st5	median	pre	IQR	0.5	5	25	-0.48408	-0.11754	0.59822	Wilcoxon Matched Pairs Test: 1st5 pre vs. 1st5 post: ****: p=1.788e-7 last5 pre vs. last5 post: ****: p=5.960e-8 Mann-Whitney U-test: 1st5 post-pre vs. last5 post-pre: **: p=0.001785
				1st5	median	post	IQR		5	25	1.77838	2.22310	3.03070	
				1st5	median	post-pre	IQR		5	25	1.50187	2.42933	2.98327	
				last5	median	pre	IQR		5	25	-0.55732	-0.13022	0.28528	
				last5	median	post	IQR		5	25	0.83224	1.19049	1.71317	
				last5	median	post-pre	IQR		5	25	1.06862	1.36688	1.75506	
**FigS15H, Fig8H**	Isosbestic	7	tone cue	1st5	median	pre	IQR	0.5	5	25	-0.06994	0.14412	0.30778	Wilcoxon Matched Pairs Test: 1st5 pre vs. 1st5 post: p=0.1817 last5 pre vs. last5 post: p=0.8119 Mann-Whitney U-test: 1st5 post-pre vs. last5 post-pre: p=0.1870
				1st5	median	post	IQR		5	25	-0.36084	0.03999	0.27007	
				1st5	median	post-pre	IQR		5	25	-0.45842	-0.10817	0.17054	
				last5	median	pre	IQR		5	25	-0.08624	0.05515	0.29273	
				last5	median	post	IQR		5	25	-0.14940	0.13012	0.39986	
				last5	median	post-pre	IQR		5	25	-0.28559	-0.00259	0.36524	
	Ca2+			1st5	median	pre	IQR	0.5	5	25	-0.64216	-0.21927	0.11812	Wilcoxon Matched Pairs Test: 1st5 pre vs. 1st5 post: **: p=0.00251 last5 pre vs. last5 post: p=0.1199 Mann-Whitney U-test: 1st5 post-pre vs. last5 post-pre: p=0.1683
				1st5	median	post	IQR		5	25	-0.09904	0.39016	1.03699	
				1st5	median	post-pre	IQR		5	25	-0.10306	0.49315	1.34135	
				last5	median	pre	IQR		5	25	-0.31478	0.16310	0.44764	
				last5	median	post	IQR		5	25	0.01391	0.42774	0.77949	
				last5	median	post-pre	IQR		5	25	-0.29107	0.18045	0.91541	

continued on next page…

Figure	channel	day #	day name	event ID	quant	diff or val	unit	t windows (s)	n mice	n stat	quartile 1	median	quartile 3	statistical test and p-value
**FigS15L, Fig8L**	Isosbesti	6, 7	airpuff	1st5	median	post-pre	IQR	0.5	5	25	*-0.24888*	*0.00187*	*0.23706*	*(same median and quartile data as above)*<br>Mann-Whitney U-test:<br>1st5 post-pre airpuff vs. 1st5 post-pre LED light isosbestic: p=0.1806<br>1st5 post-pre airpuff vs. 1st5 post-pre tone isosbestic: p=0.2143<br>1st5 post-pre LED light vs. 1st5 post-pre tone isosbestic: p=0.9227<br>1st5 post-pre airpuff vs. 1st5 post-pre LED light Ca2+ dependent: *: p=0.03971<br>1st5 post-pre airpuff vs. 1st5 post-pre tone Ca2+ dependent: ****: p=1.9973e-6<br>1st5 post-pre LED light vs. 1st5 post-pre tone Ca2+ dependent: ****: p=3.5836e-5
			LED light	1st5	median	post-pre	IQR		5	25	*-0.33401*	*-0.13710*	*0.08992*	
			tone cue	1st5	median	post-pre	IQR		5	25	*-0.45842*	*-0.10817*	*0.17054*	
	Ca2+ dependent		airpuff	1st5	median	post-pre	IQR		5	25	*1.97567*	*3.09827*	*4.14886*	
			LED light	1st5	median	post-pre	IQR		5	25	*1.50187*	*2.42933*	*2.98327*	
			tone cue	1st5	median	post-pre	IQR		5	25	*-0.10306*	*0.49315*	*1.34135*	
**FigS15O, Fig8O**	Isosbestic	8	airpuff series (1st airpuff in each train VS 5th airpuff in each train)	1st in train	median	pre	IQR	0.2	5	50	-0.26681	0.13712	0.33247	Wilcoxon Matched Pairs Test:<br>1st in train pre vs. 1st in train post: p=0.06070<br>5th in train pre vs. 5th in train post: p=0.5399<br>Mann-Whitney U-test:<br>1st in train post-pre vs. 5th in train post-pre: p=0.3574
				1st in train	median	post	IQR		5	50	-0.26390	0.03055	0.19022	
				1st in train	median	post-pre	IQR		5	50	-0.47060	-0.12512	0.15710	
				5th in train	median	pre	IQR		5	50	-0.19965	0.03260	0.36577	
				5th in train	median	post	IQR		5	50	-0.18943	0.05202	0.21433	
				5th in train	median	post-pre	IQR		5	50	-0.27969	-0.06504	0.37078	
	Ca2+ dependent			1st in train	median	pre	IQR		5	50	-1.00033	-0.57559	-0.19928	Wilcoxon Matched Pairs Test:<br>1st in train pre vs. 1st in train post: ****: p=3.553e-15<br>5th in train pre vs. 5th in train post: ****: p=6.011e-6<br>Mann-Whitney U-test:<br>1st in train post-pre vs. 5th in train post-pre: ****: p=2.030e-14
				1st in train	median	post	IQR		5	50	1.27182	2.20274	3.51884	
				1st in train	median	post-pre	IQR		5	50	1.71332	2.45398	4.48078	
				5th in train	median	pre	IQR		5	50	-0.12980	0.29413	0.71773	
				5th in train	median	post	IQR		5	50	0.41310	0.73526	1.18267	
				5th in train	median	post-pre	IQR		5	50	0.04757	0.30260	0.84576	

TableS3. Statistical details for *in vivo* and *in vitro* electrophysiological and genetic experiments and neuronal reconstructions

figure	day #	experimental condition	event ID	quantity	diff or val	unit	t window (s)	n_mice	n_stat	quartile 1	median	quartile 3	statistical test and p-value
Fig2G, Fig2I, Fig2J	4	habituation	T1	median firing rate	baseline	Hz	20	6	21 units	9.39	18.08	32.52	Wilcoxon Matched Pairs Test:<br>habituation T1 baseline vs. habituation T1 post: n.s.: p=0.11<br>conditioning T12 baseline vs. conditioning T12 post: **:p=0.0080<br>recall T1 baseline vs. recall T1 post: **:p=0.0039<br>recall T2 baseline vs. recall T2 post: ***: p=0.00049<br>recall T3 baseline vs. recall T3 post **: p=0.0020<br>recall T4 baseline vs. recall T4 post: n.s.: p=0.31<br>recall T5 baseline vs. recall T5 post: n.s.: p=0.08<br><br>Regression:<br>habituation: n.s.: p=0.54<br>recall: *:p=0.014
					post	Hz		6		10.39	18.88	37.21	
					post-baseline	z-score		6		-0.01	0.10	0.21	
			T2		post-baseline	z-score		6		-0.04	0.04	0.16	
			T3		post-baseline	z-score		6		-0.05	0.02	0.19	
			T4		post-baseline	z-score		6		-0.08	-0.01	0.10	
			T5		post-baseline	z-score		6		-0.06	0.02	0.19	
		conditioning	T12		baseline	Hz		6		11.99	22.08	47.65	
					post	Hz		6		19.83	30.22	62.29	
	5	recall	T1		baseline	Hz		4	13 units	6.69	18.68	22.93	
					post	Hz		4		15.13	31.07	41.91	
					post-baseline	z-score		4		-0.02	0.28	0.74	
			T2		baseline	Hz		4		0.85	14.99	22.38	
					post	Hz		4		22.03	26.12	36.46	
					post-baseline	z-score		4		0.23	0.43	1.06	
			T3		baseline	Hz		4		0.45	18.93	21.58	
					post	Hz		4		5.64	25.17	34.12	
					post-baseline	z-score		4		0.07	0.21	0.53	
			T4		baseline	Hz		4		0.55	12.74	24.18	
					post	Hz		4		0.85	20.28	30.27	
					post-baseline	z-score		4		-0.04	0.04	0.18	
			T5		baseline	Hz		4		0.75	13.99	23.58	
					post	Hz		4		4.10	19.03	27.52	
					post-baseline	z-score		4		-0.11	-0.04	0.05	

continued on next page…

figure	day #	experimental condition	event ID	quantity	diff or val	unit	t window (s)	n_mice	n_stat	quartile 1	median	quartile 3	statistical test and p-value
**Fig3I**	4	nolight	T1	median firing rate	post-baseline	z-score	20	8	29 units	-0.024	0.165	0.333	Wilcoxon Matched Pairs Test: nolight T1 vs. light T1: ****: p=2.42e-05 nolight T2 vs. light T2: **: p=0.0024 nolight T3 vs. light T3: ****: p=1.04e-07 nolight T4 vs. light T4: ****: p=2.18e-06 nolight T5 vs. light T5: ****: p=2.24e-08 nolight T6 vs. light T6: ****: p=9.31e-08 nolight T7 vs. light T7: ****: p=3.72e-09 nolight T8 vs. light T8: ****: p=3.39e-05 nolight T9 vs. light T9: ****: p=1.12e-08 nolight T10 vs. light T10: ****: p=2.24e-08
	4	light	T1		post-baseline	z-score		8		-0.172	0.040	0.129	
	4	nolight	T2		post-baseline	z-score		8		-0.016	0.072	0.294	
	4	light	T2		post-baseline	z-score		8		-0.102	-0.032	0.100	
	4	nolight	T3		post-baseline	z-score		8		0.013	0.050	0.205	
	4	light	T3		post-baseline	z-score		8		-0.182	-0.100	-0.017	
	4	nolight	T4		post-baseline	z-score		8		-0.031	0.057	0.134	
	4	light	T4		post-baseline	z-score		8		-0.164	-0.047	0.013	
	4	nolight	T5		post-baseline	z-score		8		-0.060	0.038	0.145	
	4	light	T5		post-baseline	z-score		8		-0.215	-0.166	-0.059	
	4	nolight	T6		post-baseline	z-score		8		-0.015	0.059	0.109	
	4	light	T6		post-baseline	z-score		8		-0.199	-0.076	0.011	
	4	nolight	T7		post-baseline	z-score		8		-0.033	0.022	0.141	
	4	light	T7		post-baseline	z-score		8		-0.229	-0.145	-0.065	
	4	nolight	T8		post-baseline	z-score		8		0.000	0.050	0.182	
	4	light	T8		post-baseline	z-score		8		-0.179	-0.053	0.030	
	4	nolight	T9		post-baseline	z-score		8		-0.029	0.067	0.135	
	4	light	T9		post-baseline	z-score		8		-0.186	-0.140	-0.079	
	4	nolight	T10		post-baseline	z-score		8		-0.030	0.027	0.119	
	4	light	T10		post-baseline	z-score		8		-0.234	-0.122	-0.047	
**Fig3M**	4	nolight	-	median pupil diameter	post-baseline	z-score	20	6	6 (median of 10 tones)	1.83	2.85	7.09	Wilcoxon Matched Pairs Test: baseline vs. tones: *: p=0.031
	4	light	-		post-baseline	z-score		6		-0.46	-0.16	4.31	
**Fig3K**	4	recall behavior	-	median freezing	baseline	% time	20	8	8 (median of 5 tones)	0.02	0.03	0.06	Wilcoxon Matched Pairs Test: baseline vs. tones: **: p=0.0078
	4		-		tone	% time	20	8		0.11	0.12	0.42	

continued on next page…

figure	day #	experimental condition	event ID	quantity	diff or val	unit	t window (s)	n_mice	n_stat	quartile 1	median	quartile 3	statistical test and p-value
**FigS7C**	-	*in vitro*	-	maximal instantane-ous firing frequency	*Type eN*	Hz	-	10	40	80.58	168.54	254.83	Mann-Whitney U-test: *Type eN* vs. *Type eB* ****: p=6.55e-8
	-	*in vitro*	-		*Type eB*	Hz	-	9	33	24.56	31.92	57.41	
	-	*in vitro*	-	maximal AP rise slope	*Type eN*	dV dt max V/s	-	11	40	437.22	502.65	556.55	Mann-Whitney U-test: *Type eN* vs. *Type eB* ****: p=1.97e-11
	-	*in vitro*	-		*Type eB*		-	9	34	204.57	280.62	322.55	
	-	*in vitro*	-	cellular capacitance	*Type eN*	pF	-	12	38	26.60	36.74	47.16	Mann-Whitney U-test: *Type eN* vs. *Type eB* ****: p=7.89e-9
	-	*in vitro*	-		*Type eB*	pF	-	9	32	17.53	21	24.08	
	-	*in vitro*	-	burstiness	*Type eN*	1	-	13	40	5.60	11.20	18.66	Mann-Whitney U-test: *Type eN* vs. *Type eB* ****: p=7.05e-7
	-	*in vitro*	-		*Type eB*	1	-	9	33	2.02	3.21	5.01	
	-	*in vitro*	-	AP half-width	*Type eN*	ms	-	14	40	0.27	0.28	0.33	Mann-Whitney U-test: *Type eN* vs. *Type eB* ****: p=7.55e-11
	-	*in vitro*	-		*Type eB*	ms	-	9	34	0.40	0.44	0.50	
	-	*in vitro*	-	cellular input resistance	*Type eN*	MOhm	-	15	38	102.36	168.19	262.30	Mann-Whitney U-test: *Type eN* vs. *Type eB* ****: p=6.03e-11
	-	*in vitro*	-		*Type eB*	MOhm	-	9	32	397.03	597.69	933.42	
**FigS8K**	-	*in vitro*	-	pvalb expression level	*Type gG*	norma-lized tpm	-	6	15	13.81	45.56	215.94	Kruskal-Wallis ANOVA-test: *Type gG* vs. *Type gC*: n.s.: p=0.868; *Type gG* vs. *Type gH*: *: p=0.034; *Type gC* vs. *Type gH*: **: p=0.0019
	-	*in vitro*	-		*Type gC*		-	7	13	51.15	204.86	372.06	
	-	*in vitro*	-		*Type gH*		-	6	10	0.00	0.00	2.84	
**FigS9B**	-	*in vitro*	-	longest dendritic length	*Type eN*	µm	-	6	20	407.2	449.2	530.6	Mann-Whitney U-test: *Type eN* vs. *Type eB* n.s.: p=0.134
	-	*in vitro*	-		*Type eB*	µm	-	6	8	328.2	406.9	439.3	
**FigS9C**	-	*in vitro*	-	total dendritic length	*Type eN*	µm	-	6	20	1650.9	2177.0	2682.7	Mann-Whitney U-test: *Type eN* vs. *Type eB* *: p=0.010
	-	*in vitro*	-		*Type eB*	µm	-	6	8	665.2	1459.1	1839.4	
**FigS9D**	-	*in vitro*	-	number of primary dendrites	*Type eN*	1	-	6	20	3.0	3.5	5.0	Mann-Whitney U-test: *Type eN* vs. *Type eB* n.s.: p=0.185
	-	*in vitro*	-		*Type eB*	1	-	6	8	3.0	3.0	3.5	
**FigS9E**	-	*in vitro*	-	spine density / 100µm	*Type eN*	1 / 100µm	-	6	20	0.460	0.810	1.148	Mann-Whitney U-test: *Type eN* vs. *Type eB* n.s.: p=0.431
	-	*in vitro*	-		*Type eB*	1 / 100µm	-	6	8	0.588	0.866	1.284	

TableS4. Viral constructs used in this study

Virus type	Source	Identifier
AAV/DJ-hSyn-CreON/FlpON-ChR2-eYFP	UNC Vector Core	AV6214C
AAV2/1-hSyn-FLEX-GCaMP6f-WPRE-SV40	Penn Vector Core	AV-1-PV2822
AAV2/2-EF1α-DIO-mCherry	UNC Vector Core	AV4375E
AAV2/5-EF1α-DIO-hChR2(H134R)-eYFP	UNC Vector Core	20298-AAV5
AAV2/8-hSyn-DIO-hM3D(Gq)-mCherry	Addgene	44361-AAV8
AAV2/8-hSyn-DIO-hM4D(Gi)-mCherry	Addgene	44362-AAV8
AAV2/8-hSyn-DIO-mCherry	Addgene	50459-AAV8
AAV2/9-EF1α-DO-hChR2(H134R)-mCherry-WPREpA	Salk GT3 Vector Core	Plasmid #37082
AAVretro11-CAG-FLEX-ArchT-EGFP-WPREpA	BrainCase	BC-0228
AAVretro11-EF1α-DIO-EYFP	BrainCase	BC-0242
AAV2/1-EF1α-DIO-FlpO-mCherry	BrainCase	BC-0179
AAV2/1-EF1α-fDIO-eYFP	Salk GT3 Vector Core	Addgene: 55641
AAVretro-EF1α-DIO-ChR2-mCherry	Addgene	20297-AAVrg
AAVretro-EF1α-DIO-FlpO	Addgene	87306-AAVrg
AAVretro-EF1α-DIO-mCherry	UNC Vector Core	50459-AAVrg
AAVretro-hSyn-DIO-eGFP	Addgene	50457-AAVrg
AAVretro-hSyn-FLEX-jGCaMP8m-WPRE	Addgene	162378-AAVrg
AAV2/5-CAG-FLEX-ArchT-GFP	UNC Vector Core	AV4960C
AAV2/5-EF1α-DIO-eYFP	Addgene	27056-AAV5

TableS5. Coordinates for viral injections, optic fiber implantations and electrodes

species	coordinate type	Region abbreviation	Region full name	Angle around AP, from lateral (°)	Angle around ML, from caudal (°)	AP (mm)	ML (mm)	DV (mm)
mouse	virus injection	MRR	median raphe region	0	0	-4.50	0.00	-4.60
mouse	virus injection	MRR	median raphe region	15	0	-4.30	1.25	-4.55
mouse	virus injection	LHb	lateral habenula	0	0	-1.60	0.40	-2.80
mouse	virus injection	BLA	basolateral amygdala	0	0	-1.90	3.00	-4.60
mouse	virus injection	vHipp	ventral hippocampus	0	0	3.20	3.00	-4.00
mouse	virus injection	vHipp	ventral hippocampus	0	0	3.20	3.00	-2.60
mouse	virus injection	mPFC	medial prefrontal cortex	0	0	1.80	0.30	-3.00
rat	virus injection	MRR	median raphe region	0	0	-8.00	0.00	-8.40
rat	virus injection	LHb	lateral habenula	0	0	-3.60	0.60	-5.00
mouse	optic fiber	MRR	median raphe region	0	0	-4.50	0.00	-4.23
mouse	optic fiber	MRR	median raphe region	15	0	-4.30	1.25	-4.55
mouse	optic fiber	MRR	median raphe region	20	0	-4.55	1.40	-4.00
mouse	optic fiber	MRR	median raphe region	0	20	-6.10	0.00	-4.80
mouse	optic fiber	mPFC	medial prefrontal cortex	0	0	1.60	0.30	-1.50
mouse	optic fiber	mPFC	medial prefrontal cortex	0	0	1.70	0.50	-2.00
mouse	optic fiber	mPFC	medial prefrontal cortex	26	0	1.50	1.40	-1.60
mouse	optic fiber	LHb	lateral habenula	0	0	-1.4	0.4	-2.1
mouse	optic fiber	LHb	lateral habenula	20	0	-1.4	1.25	-1.8
mouse	silicon probe	MRR	median raphe region	0	20	-6.10	0.00	-4.20
mouse	silicon probe	LHb	lateral habenula	14	0	-1.80	1.30	until emerging unit activity below DG
mouse	ground electrode	CB	cerebellum	0	0	-5.60	2.00	above

TableS6. Primary antibodies used in this study

Antigen or reagent	Host	Dilution	Source	Catalog number	Specificity
cFos	Rabbit	1:1000	Synaptic Systems	226,003	Specific labeling for cfos
RFP	Rat	1:2000	Chromotek	5F8	No staining in animals not injected with mCherry-expressing virus
PV	Mouse	1:2000	Swant	PV235	KO verified
vGAT	Guinea pig	1:2000	Synaptic Systems	131 004	KO verified
vGluT2	Rabbit	1:2000	Synaptic Systems	135,402	Specific for the mammalian vGluT2
Homer	Rabbit	1:2000	Synaptic Systems	160,003	Specific for Homer 1. Cross-reactivity of the serum to Homer 2 and 3 was removed by pre-adsorption with Homer 2 (aa 1 - 176) and Homer 3 (aa 1 - 177).
eGFP	Chicken	1:2000	ThermoFisher Scientific	A10262	No staining in mice not injected with eGFP-expressing virus
eGFP	Rabbit	1:1000	Invitrogen	11122	Same staining as native eGFP and also verified by GFP target fusion tests

TableS7. Secondary antibodies used in this study

Raised in	Raised against	Conjugated with	Dilution	Source	Catalog number
Goat	Chicken	Alexa 488	1:1000	ThermoFisher Scientific	A11039
Donkey	Rabbit	Alexa 647	1:500	Jackson Immunoresearch	711-605-152
Donkey	Rat	Alexa 594	1:500	ThermoFisher Scientific	A21209
-	Streptavidin	Alexa 488	1:2000	ThermoFisher Scientific	S11223
Horse	Mouse	biotinylated	1:500	Vector Laboratories	BA-2000
Donkey	Rabbit	Alexa 594	1:500	ThermoFisher Scientific	A21207
Donkey	Guinea pig	Alexa 647	1:500	Jackson Immunoresearch	706-605-148
Donkey	Rabbit	Alexa 488	1:1000	ThermoFisher Scientific	A21206
Donkey	Chicken	Alexa 647	1:500	Jackson Immunoresearch	AP194SA6
Donkey	Mouse	Cy5	1:500	Jackson Immunoresearch	715-175-151
Goat	Rabbit	1.4 nm gold (Fab')	1:200	NanoProbes	2004-1ML
Goat	Rat	biotinylated	1:1000	Jackson Immunoresearch	112-066-062

TableS8. Primary and secondary antibody combinations used in immunofluorescent, immunoperoxidase and electron microscopic experiments

related figure	animal species / strain	experiment types	primary antibodies or reagents	secondary antibodies	note
**Fig1B, FigS1G, FigS3, FigS10B-C**	vGluT1-Cre, vGluT2-Cre, PV-Cre	anatomical, behavioral and fiber photometry	chicken-anti-eGFP	Alexa 488-conjugated goat-anti-chicken	Intensifying native green fluorescent signal
**Fig4F, FigS4D, Fig7B-C, K-L, FigS12E, G-H, Fig9B-C**	vGluT1-Cre, vGluT2-Cre	anatomical, behavioral	rat-anti-RFP	Alexa 594-conjugated donkey-anti-rat	Intensifying native red fluorescent signal
**Fig9G, Fig9I**	TRAP2	reactivation	DAPI	-	Labeling c-Fos positive neurons in the mPFC in the TRAP2 experiment.
			chicken-anti-eGFP	Alexa 488-conjugated goat-anti-chicken	
			rabbit-anti-c-Fos	Alexa 594-conjugated donkey-anti-rabbit	
**Fig4C**	vGluT2-Cre	early gene activation	DAPI	-	Labeling c-Fos positive neurons in MRR after corticosterone treatment experiment.
			chicken-anti-eGFP	Alexa 488-conjugated goat-anti-chicken	
			rabbit-anti-c-Fos	Alexa 647-conjugated donkey-anti-rabbit	
**Fig7E-G, FigS12B, C, D, FigS13 Fig8B, Fig10C-D, G-I**	vGluT1-Cre, vGluT2-Cre	postsynaptic structure labeling	chicken-anti-eGFP	Alexa 488-conjugated goat-anti-chicken	Labeling Homer positive putative synapses in the mPFC and the MRR to characterize the connections between the BLA or the vHipp and the mPFC; also between the mPFC and the MRR.
			rat-anti-RFP	Alexa 594-conjugated donkey-anti-rat	
			rabbit-anti-Homer	Alexa 647-conjugated donkey-anti-rabbit	
**Fig6B, C, E, F**	vGluT2-Cre	PV positivity of MRR^{vGluT2} neurons	DAPI	-	Labeling of MRR neurons and PV.
			chicken-anti-eGFP	Alexa 488-conjugated goat-anti-chicken	
			mouse-anti-PV	Cy5-conjugated donkey-anti-mouse	
**Fig5F**	vGluT2-Cre	reconstruction of *in vitro* filled neurons	DAPI	-	Labeling individual MRR^{vGluT2} neurons for anatomical localization and for cell reconstruction after *in vitro* recordings.
			Alexa 488-conjugated Streptavidin	-	
			rat-anti-RFP	Alexa 594-conjugated donkey-anti-rat	
**Fig6G-H**	vGluT2-Cre	PV staining in the mouse	mouse-anti-PV	biotinylated horse-anti-mouse	Labeling MRR^{PV} neurons in the mouse brainstem.
**Fig6I-J**	Wistar rat	PV staining in the rat	mouse-anti-PV	biotinylated horse-anti-mouse	Labeling MRR^{PV} neurons in the rat brainstem.
**Fig6K-L**	Rhesus macaque	CTRL staining in macaque	-	biotinylated horse-anti-mouse	CTRL staining for PV antibody verification in macaque brainstem.
**Fig6M-N**	Rhesus macaque	PV staining in macaque	mouse-anti-PV	biotinylated horse-anti-mouse	Labeling MRR^{PV} neurons in macaque brainstem.

continued on next page…

related figure	animal species / strain	experiment types	primary antibodies or reagents	secondary antibodies	note
**FigS10E-G**	PV-Cre	MRR^{PV} axonal neurotransmitter analysis in the LHb	chicken-anti-eGFP	Alexa 488-conjugated goat-anti-chicken	vGluT2 and vGAT staining on virally labeled MRR^{PV} axonal terminals in LHb.
			rabbit-anti-vGluT2	Alexa 594-conjugated donkey-anti-rabbit	
			guinea pig-anti-vGAT	Alexa 647-conjugated donkey-anti-guinea pig	
**FigS11**	Wistar rat	rat MRR axonal neurotransmitter analysis in the LHb	DAPI	-	vGluT2 and vGAT staining on virally labeled MRR^{PV} axonal terminals in the LHb in the rat.
			rabbit-anti-vGluT2	Alexa 488-conjugated donkey-anti-rabbit	
			rat-anti-RFP	Alexa 594-conjugated donkey-anti-rat	
			guinea pig-anti-vGAT	Alexa 647-conjugated donkey-anti-guinea pig	
**Fig10N-P**	vGluT1-Cre	anatomy: BLA and vHipp->mPFC	DAPI	-	Labeling the traced vHipp and BLA axons in the mPFC and in the MRR
			chicken-anti-eGFP	Alexa 488-conjugated goat-anti-chicken	
			rat-anti-RFP	Alexa 594-conjugated donkey-anti-rat	
**FigS10I-J**	PV-Cre	anatomy: PV DIO and DO	DAPI	-	Labeling the traced MRR^{PV} and MRR_nonPV axonal projection in the LHb and verifying their vGluT2 content.
			chicken-anti-eGFP	Alexa 488-conjugated goat-anti-chicken	
			rat-anti-RFP	Alexa 594-conjugated donkey-anti-rat	
			rabbit-anti-vGluT2	Alexa 594-conjugated donkey-anti-rabbit	
**FigS13**	vGluT2-Cre	anterograde transsynaptic anatomy experiments	chicken-anti-eGFP	Alexa 488-conjugated goat-anti-chicken	Labeling the traced MRR axons that received synapses from mPFC in the LHb.
			rat-anti-RFP	Alexa 594-conjugated donkey-anti-rat	
**Fig7H-I, Fig10E, J-L, FigS14**	vGluT1-Cre	electron microscopy	-	-	Labeling presynaptic and postsynaptic components to verify the synaptic connections using electron microscopy.
			rabbit-anti-eGFP	1.4 nm gold (Fab')-conjugated goat-anti-rabbit	
			rat-anti-RFP	biotinylated goat-anti-rat	
**Fig8A, FigS15A**	vGluT1-Cre	fiber photometry	DAPI	-	Intensifying native green fluorescent signal
			chicken-anti-eGFP	Alexa 488-conjugated goat-anti-chicken	
**Fig8A, FigS15A**	vGluT1-Cre	fiber photometry	DAPI	-	Intensifying native green fluorescent signal
			chicken-anti-eGFP	Alexa 647-conjugated donkey-anti-chicken	

TableS9. Electrophysiological parameters of MRR$^{vGluT2 \rightarrow LHb}$ neurons measured *in vitro*, presented without normalization

MRR = Median Raphe Region	electrophysiological type based on cluster analysis	RMP = resting membrane potential	input resistance measured in voltage clamp mode	cellular capacitance based on the area under the current curve	membrane time constant	ratio of the steady-state voltage response and the peak upon hyperpolarization	dVdt max = maximal rate of voltage change over time in the action potential rising phase	dVdt min = maximal rate of voltage change over time in the action potential falling phase	threshold for action potential generation	amplitude of action potential (threshold to peak)	rise time between threshold and peak	action potential half width	action potential afterhyperpolarization amplitude (threshold to AHP peak)			time interval between the 1st and the 2nd AP (action potential) within an AP train	adaptation of the AP amplitude within an AP train (1st/last)	adaptation of the AP half-width within an AP train (1st/last)	adaptation of the instantaneous AP frequency within an AP train (1st/last)	maximal instantaneous AP firing frequency	max freq / (mean AP firing frequency within an AP train)
**MRR cell ID**	**Type**	**RMP (mV)**	**Rin VC (MΩ)**	**Cm AUC (pF)**	**tau (ms)**	**sag (V/V)**	**dVdt max (V/s)**	**dvdt min (V/s)**	**threshold @ dvdt 100 (mV)**	**amplitude (mV)**	**RT (ms)**	**HW (ms)**	**AHP ampl (mV)**	**time to AHP peak (ms)**	**dVdt max / dVdt min (1)**	**1st spike interval (ms)**	**amplitude adaptation**	**HW adaptation (ms/ms)**	**freq adaptation (Hz/Hz)**	**max freq (Hz)**	**burstiness (Hz/Hz)**
**19591**	*Type eN*	-50.8	108.5	47.16	9.009	0.819	783.0	-376.0	-38.4	84.7	0.210	0.270	-18.6	0.710	2.082	12.59	0.954	1.085	0.074	256.4	13.5
**19592**	*Type eN*	-56.2	102.4	43.51	10.425	0.817	857.0	-478.0	-37.2	83.3	0.210	0.220	-26.6	0.480	1.793	16.97	0.926	1.099	0.218	58.9	3.3
**19631**	*Type eB*	-59.7	478.2	19.79	23.527	1.030	358.0	-113.0	-24.9	66.9	0.415	0.623	-25.4	1.620	3.168	30.16	0.881	1.422	0.317	33.2	2.6
**197519**	*Type eN*	-62.5	357.3	22.28	17.632	0.931	511.5	-367.1	-31.8	73.7	0.236	0.268	-30.8	0.560	1.393	5.03	0.924	1.100	0.025	324.7	23.2
**197831**	*Type eB*	-70.1	589.3	17.33	15.707	1.184	140.6	-109.0	-11.9	33.1	0.340	0.404	-43.3	1.876	1.289	462.00	0.773	1.430	0.464	24.2	2.0
**197832**	*Type eB*	-52.7	1304.8	16.91	32.542	0.955	169.1	-90.1	-23.3	44.7	0.416	0.544	-33.7	2.908	1.878	60.93	0.979	1.199	0.172	30.0	5.0
**197833**	*Type eB*	-58.6	893.2	16.86	27.937	0.991	291.5	-196.2	-26.8	60.6	0.336	0.388	-34.1	1.228	1.486	140.49	0.860	1.302	0.465	18.8	1.7
**197934**	*Type eB*	-57.9	442.0	19.23	20.477	1.058	106.5	-86.5	-0.6	23.0	0.332	0.380	-44.5	1.884	1.231	162.00	0.830	1.210	0.530	24.6	4.9
**197935**	*Type eB*	-52.3	796.0	11.03	35.837	0.896	244.8	-167.5	-27.2	54.7	0.356	0.412	-36.3	1.532	1.461	174.00	0.858	1.241	0.694	17.0	1.4
**198036**	*Type eB*	-38.4	n.a	n.a	33.539	0.976	202.3	-88.7	-28.4	56.9	0.444	0.620	-23.5	4.552	2.282	619.59	0.897	1.579	0.279	31.9	2.9
**198037**	*Type eN*	-48.9	170.8	34.08	11.513	0.841	521.7	-277.5	-37.1	77.2	0.252	0.312	-20.4	0.944	1.880	137.00	0.940	1.111	0.222	32.4	2.9
**198039**	*Type eB*	-49.8	1054.4	15.96	31.439	0.946	284.6	-139.2	-17.3	56.5	0.324	0.428	-40.8	1.604	2.044	67.00	0.690	2.024	0.407	46.7	2.2
**1981390**	*Type eN*	-47.8	104.0	43.48	13.060	0.874	590.8	-439.3	-39.3	73.7	0.200	0.230	-25.7	0.480	1.345	49.00	0.970	1.057	0.089	64.3	4.3
**198140**	*Type eN*	-52.2	190.9	21.26	7.185	0.936	399.4	-139.1	-29.7	71.5	0.316	0.460	-16.2	1.384	2.872	10.23	0.989	1.088	0.028	145.0	8.1
**198141**	*Type eB*	-41.5	459.8	22.48	17.056	0.745	140.1	-64.3	-3.7	33.1	0.380	0.548	-46.1	3.896	2.178	147.00	0.441	3.453	0.258	25.0	4.2
**198242**	*Type eN*	-46.6	283.2	26.00	16.424	1.058	546.5	-273.7	-37.6	81.4	0.268	0.344	-22.8	0.824	1.996	4.03	0.910	1.144	0.040	261.5	10.5
**198243**	*Type eN*	-54.0	103.9	47.45	11.354	0.898	488.4	-291.7	-27.7	72.0	0.236	0.288	-23.5	0.688	1.674	4.19	0.831	1.294	0.213	265.3	18.9
**198344**	*Type eN*	-45.7	184.9	36.20	15.680	0.910	540.5	-434.7	-38.8	72.4	0.228	0.248	-25.1	0.472	1.243	4.83	0.974	1.030	0.007	341.4	24.4
**198345**	n.a	n.a	n.a	n.a	n.a	n.a	n.a	n.a	n.a	n.a	n.a	n.a	n.a	n.a	n.a	n.a	n.a	n.a	n.a	n.a	n.a
**198548**	*Type eN*	-65.2	262.3	30.98	13.915	1.001	586.7	-344.4	-34.1	79.3	0.232	0.280	-29.2	0.624	1.704	6.34	0.966	1.048	0.064	191.9	17.4
**198549**	*Type eN*	-49.6	558.5	26.45	22.830	0.986	419.4	-222.4	-30.5	71.9	0.284	0.352	-27.9	0.864	1.886	5.62	0.968	1.114	0.025	207.3	23.0
**198650**	*Type eB*	-41.7	476.2	17.53	22.727	0.964	240.6	-99.6	-19.7	46.3	0.324	0.456	-29.4	1.872	2.416	26.89	0.852	1.486	0.255	37.2	3.7
**198751**	*Type eB*	-46.3	1169.0	17.54	41.222	0.883	204.6	-73.5	-19.1	53.0	0.428	0.656	-39.5	2.948	2.784	147.00	0.743	1.619	0.722	22.3	1.4
**198752**	*Type eB*	-40.8	384.6	27.20	20.311	0.779	171.2	-60.3	-23.6	46.6	0.444	0.700	-25.4	4.552	2.836	302.00	0.937	1.438	0.178	31.9	3.2
**198753**	*Type eB*	-54.2	283.2	26.68	21.180	0.981	307.6	-170.1	-19.7	65.1	0.352	0.432	-30.1	1.128	1.809	9.32	0.892	1.250	0.038	119.9	8.6
**198754**	*Type eB*	-52.2	771.5	21.68	20.020	0.989	437.3	-202.4	-26.3	70.0	0.276	0.372	-37.5	1.080	2.161	21.20	0.890	1.198	0.117	68.2	5.7
**198855**	*Type eB*	-44.7	407.1	18.75	12.494	0.993	394.5	-132.8	-34.4	73.8	0.332	0.504	-23.7	2.260	2.971	724.00	0.927	1.220	0.343	33.2	3.0
**198856**	*Type eN*	-48.2	93.9	48.05	11.226	0.949	464.4	-353.6	-31.5	65.8	0.228	0.256	-24.7	0.640	1.313	10.17	0.919	1.129	0.071	164.3	7.5
**198957**	*Type eN*	-59.7	230.1	33.00	16.992	0.912	436.5	-197.5	-32.2	72.0	0.280	0.368	-22.2	1.036	2.210	7.38	0.892	1.238	0.085	171.8	15.6
**198958**	*Type eN*	-52.5	324.5	21.30	21.860	0.935	466.1	-237.7	-32.1	73.3	0.268	0.336	-26.4	0.852	1.961	26.92	0.932	1.131	0.085	239.5	20.0
**199273**	*Type eB*	-55.3	355.6	20.83	16.041	0.911	297.1	-158.0	-32.3	65.5	0.356	0.448	-20.8	1.152	1.881	123.57	0.827	1.358	0.197	43.0	4.3
**199274**	*Type eB*	-50.0	237.0	21.10	27.918	1.035	172.6	-109.2	-25.7	48.0	0.412	0.504	-27.7	1.808	1.580	236.00	0.770	1.335	0.267	39.4	4.9
**199375**	*Type eB*	-88.3	255.4	31.50	9.394	1.056	322.6	-247.7	-31.4	65.1	0.328	0.368	-26.1	0.928	1.302	120.18	0.881	1.206	0.621	27.7	1.7
**199376**	*Type eB*	-68.2	287.8	38.65	14.121	1.018	276.6	-132.8	-21.6	53.4	0.320	0.440	-26.2	1.360	2.082	112.98	0.784	1.310	0.515	18.9	1.7
**199377**	*Type eB*	-70.7	386.9	26.95	40.489	1.004	253.7	-161.5	-15.7	54.0	0.340	0.396	-34.0	1.348	1.570	99.06	0.921	1.136	0.893	16.4	1.6
**199378**	*Type eB*	-52.0	727.3	19.89	19.007	1.033	216.5	-109.2	-0.1	48.0	0.356	0.452	-37.0	1.676	1.982	9.71	0.896	1.530	0.050	107.5	13.4

continued on next page…

MRR = Median Raphe Region	electrophysiological type based on cluster analysis	RMP = resting membrane potential	input resistance measured in voltage clamp mode	cellular capacitance based on the area under the current curve	membrane time constant	ratio of the steady-state voltage response and the peak upon hyperpolarization	dVdt max = maximal rate of voltage change over time in the action potential rising phase	dVdt min = maximal rate of voltage change over time in the action potential falling phase	threshold for action potential generation	amplitude of action potential (threshold to peak)	rise time between threshold and peak	action potential half width	action potential afterhyperpolarization amplitude (threshold to AHP peak)			time interval between the 1st and the 2nd AP (action potential) within an AP train	adaptation of the AP amplitude within an AP train (1st/last)	adaptation of the AP half-width within an AP train (1st/last)	adaptation of the instantaneous AP frequency within an AP train (1st/last)	maximal instantaneous AP firing frequency	max freq / (mean AP firing frequency within an AP train)
**MRR cell ID**	**Type**	**RMP (mV)**	**Rin VC (MΩ)**	**Cm AUC (pF)**	**tau (ms)**	**sag (V/V)**	**dVdt max (V/s)**	**dvdt min (V/s)**	**threshold @ dvdt 100 (mV)**	**amplitude (mV)**	**RT (ms)**	**HW (ms)**	**AHP ampl (mV)**	**time to AHP peak (ms)**	**dVdt max / dVdt min (1)**	**1st spike interval (ms)**	**amplitude adaptation (V/V)**	**HW adaptation (ms/ms)**	**freq adaptation (Hz/Hz)**	**max freq (Hz)**	**burstiness (Hz/Hz)**
**199379**	*Type eB*	-42.2	1335.4	19.16	26.670	1.022	180.9	-112.0	-11.1	41.2	0.344	0.424	-41.4	2.124	1.616	151.00	0.775	1.366	0.137	57.4	7.2
**199480**	*Type eN*	-39.1	n.a	n.a	13.555	0.954	503.2	-262.4	-37.3	66.5	0.220	0.284	-24.8	2.528	1.918	44.75	0.928	1.141	0.197	124.3	8.3
**199481**	*Type eN*	-52.5	221.5	26.60	15.583	0.662	551.3	-309.4	-37.3	78.2	0.240	0.300	-24.8	0.732	1.782	4.72	0.951	1.063	0.024	272.9	16.1
**199482**	*Type eN*	-52.6	321.1	26.28	28.295	1.014	388.4	-168.3	-27.2	71.2	0.316	0.432	-26.2	1.120	2.308	7.80	0.890	1.264	0.063	131.4	26.3
**199483**	*Type eN*	-57.4	258.1	35.43	25.421	1.043	504.8	-293.5	-33.8	75.4	0.248	0.300	-20.5	0.768	1.720	9.75	0.960	1.051	0.030	246.4	22.4
**199584**	*Type eB*	-63.9	961.0	21.04	27.356	0.949	253.7	-135.2	-22.8	55.1	0.340	0.436	-28.8	1.392	1.877	28.79	0.931	1.435	0.074	98.3	12.3
**199585**	*Type eB*	-67.2	1305.5	17.23	22.280	1.021	208.5	-98.9	-21.1	48.9	0.360	0.500	-33.3	1.772	2.108	33.42	0.939	1.303	0.105	77.7	13.0
**199586**	*Type eN*	-54.0	129.1	24.23	5.330	0.707	369.5	-340.2	-29.8	60.8	0.260	0.276	-23.2	0.612	1.086	56.74	0.953	1.107	0.080	57.5	5.8
**199587**	*Type eB*	-65.0	415.9	21.04	6.938	1.144	296.4	-141.2	-26.3	64.8	0.348	0.472	-25.7	1.412	2.099	130.54	0.933	1.223	0.236	28.9	3.2
**199688**	*Type eN*	-55.0	268.9	35.48	16.718	1.033	428.2	-166.1	-37.0	77.2	0.304	0.440	-17.2	1.284	2.577	17.38	0.916	1.177	0.159	94.0	8.5
**199689**	*Type eN*	-49.0	87.0	37.28	8.426	0.851	629.0	-235.0	-37.5	73.7	0.224	0.337	-21.0	9.073	2.677	124.00	0.966	1.060	0.312	20.8	2.3
**199690**	*Type eN*	-44.9	63.2	46.18	9.563	0.930	573.9	-332.5	-31.7	76.7	0.224	0.272	-20.1	0.676	1.726	16.74	0.964	1.060	0.069	205.3	13.7
**199691**	*Type eN*	-58.6	95.8	37.88	12.188	0.981	588.3	-368.2	-36.6	78.2	0.224	0.264	-20.4	0.548	1.598	3.03	0.989	1.025	0.016	340.9	26.2
**199792**	*Type eB*	-55.2	628.1	22.49	25.043	0.962	453.0	-221.6	-33.7	73.3	0.280	0.352	-28.1	1.012	2.044	354.00	0.974	1.103	0.163	57.5	5.2
**199793**	*Type eN*	-50.3	81.9	53.35	10.319	0.965	576.8	-335.2	-35.4	75.4	0.220	0.264	-22.9	0.644	1.721	45.81	0.969	1.025	0.178	21.6	2.7
**199794**	*Type eN*	-47.3	173.3	39.30	17.634	0.946	491.6	-230.7	-35.7	73.0	0.248	0.324	-18.7	3.188	2.131	417.00	0.967	1.078	0.410	174.1	10.9
**199895**	*Type eN*	-48.2	81.8	47.98	7.547	0.993	332.3	-223.0	-35.0	52.1	0.244	0.284	-18.6	1.024	1.490	127.00	0.993	1.049	0.167	22.4	2.8
**199896**	*Type eN*	-51.8	308.6	27.90	15.349	0.933	458.8	-307.5	-32.6	68.7	0.244	0.280	-29.3	0.688	1.492	6.59	0.981	1.044	0.029	165.3	18.4
**199897**	*Type eN*	-52.1	74.5	56.80	9.975	0.997	437.9	-311.0	-33.8	62.1	0.224	0.252	-21.9	0.916	1.408	141.00	0.967	1.047	0.256	21.8	2.2
**2003113**	*Type eB*	-66.2	606.1	21.86	15.086	0.968	416.7	-167.1	-32.3	73.0	0.292	0.424	-27.2	1.180	2.493	263.20	0.949	1.191	0.149	45.0	4.5
**2003114**	*Type eB*	-54.7	n.a	n.a	11.236	0.961	255.2	-100.8	-17.2	57.5	0.380	0.536	-32.2	1.848	2.532	527.00	n.a	n.a	n.a	n.a	n.a
**2003115**	*Type eB*	-53.5	1907.6	17.46	20.416	0.940	316.6	-120.3	-22.0	58.3	0.312	0.436	-35.8	1.680	2.633	140.94	0.948	1.210	0.422	18.7	2.1
**2004116**	*Type eN*	-47.6	296.0	23.15	18.167	0.952	400.4	-165.9	-31.5	68.6	0.292	0.404	-17.8	1.048	2.414	3.93	0.918	1.198	0.020	253.3	11.5
**2004117**	*Type eN*	-53.6	n.a	n.a	8.472	0.951	510.4	-324.4	-31.0	70.5	0.228	0.264	-22.3	0.648	1.573	68.70	0.943	1.082	0.093	44.8	4.5
**2004118**	*Type eN*	-45.1	74.2	53.80	8.204	0.895	561.8	-321.8	-29.7	73.7	0.216	0.264	-17.8	0.684	1.746	103.40	0.979	1.038	0.182	67.2	5.2
**2004119**	*Type eN*	-58.2	165.6	47.15	24.868	0.991	461.1	-243.6	-31.0	70.6	0.256	0.320	-18.9	0.816	1.893	5.00	0.883	1.258	0.103	298.7	13.6
**2004122**	*Type eN*	-56.9	363.8	29.70	20.237	0.942	404.3	-178.4	-32.2	69.4	0.288	0.380	-22.8	1.124	2.267	6.50	0.828	1.318	0.059	140.7	15.6
**2004123**	*Type eN*	n.a	136.3	45.73	12.253	0.921	481.8	-306.1	-32.2	64.9	0.216	0.256	-26.2	0.644	1.574	13.21	0.932	1.114	0.309	227.0	7.6
**2004124**	*Type eN*	-62.2	150.1	51.95	24.552	0.956	472.2	-308.1	-36.1	76.5	0.276	0.324	-22.4	0.684	1.533	4.77	0.785	1.362	0.121	296.5	10.2
**2005126**	*Type eN*	-51.4	97.6	55.85	9.139	0.951	529.3	-323.9	-33.5	71.0	0.224	0.268	-20.7	0.744	1.634	17.03	0.951	1.072	0.049	151.4	11.6
**2005127**	*Type eB*	-61.7	476.2	27.35	17.958	1.059	451.9	-212.3	-22.3	69.4	0.272	0.356	-34.4	1.036	2.129	7.09	0.917	1.165	0.068	158.1	10.5
**2006128**	*Type eB*	-52.8	728.1	23.98	28.107	0.976	319.7	-114.8	-21.3	62.7	0.332	0.508	-30.5	1.556	2.784	203.76	0.974	1.262	0.611	8.8	1.3
**2006129**	*Type eB*	-60.7	905.9	26.65	30.320	0.979	431.4	-200.7	-30.6	69.3	0.268	0.344	-32.5	1.228	2.150	163.00	0.959	1.292	0.535	26.2	1.9
**2006130**	*Type eN*	-50.5	204.9	43.70	28.376	0.981	502.1	-345.8	-31.9	73.3	0.244	0.276	-27.7	0.620	1.452	9.73	0.947	1.105	0.111	136.1	8.5
**2006131**	*Type eN*	n.a	112.2	50.53	10.050	0.845	586.6	-328.7	-33.7	74.8	0.212	0.268	-22.6	0.652	1.784	19.96	0.971	1.183	0.270	127.5	25.5
**2007132**	*Type eN*	-44.7	147.9	35.18	10.470	0.957	537.8	-367.5	-40.8	78.5	0.236	0.276	-21.9	0.556	1.463	15.69	0.867	1.215	0.310	174.4	5.5
**2007133**	*Type eB*	-71.5	1023.2	20.44	19.729	0.972	294.5	-113.0	-18.5	57.8	0.340	0.488	-38.9	1.776	2.606	144.33	0.776	1.506	0.354	26.4	2.0
**2007134**	*Type eN*	-50.3	242.6	17.86	8.817	1.022	391.6	-252.8	-31.8	61.0	0.252	0.292	-15.9	0.628	1.549	2.21	1.000	1.056	0.011	506.4	36.2
**2008136**	*Type eB*	-68.2	337.0	24.18	16.267	0.795	419.6	-225.4	-31.2	65.3	0.264	0.328	-27.0	1.012	1.861	18.58	0.835	1.307	0.168	66.3	4.4

TableS10. Participation in each analysis, localization, and clustering results of individual MRR$^{vGluT2\rightarrow LHb}$ neurons.

MRR neuron ID	Mouse ID	*In vitro* data	Genetic data	Cell reconstruction	Map localization (DV Br.)	Electrophysiology category	Genetic category
19591	Mouse1	✓	x	✓	x	*Type eN*	x
19592	Mouse1	✓	x	✓	x	*Type eN*	x
19631	Mouse2	✓	x	✓	x	*Type eB*	x
197519	Mouse3	✓	✓	x	-4.56	*Type eN*	*Type gC*
197831	Mouse4	✓	x	x	-4.56	*Type eB*	x
197832	Mouse4	✓	x	✓	-4.56	*Type eB*	x
197833	Mouse4	✓	✓	x	-4.56	*Type eB*	*Type gH*
197934	Mouse5	✓	✓	x	-4.72	*Type eB*	*Type gH*
197935	Mouse5	✓	✓	x	-4.72	*Type eB*	*Type gH*
198036	Mouse5	✓	✓	x	-4.56	*Type eB*	*Type gH*
198037	Mouse5	✓	✓	x	-4.56	*Type eN*	*Type gC*
198039	Mouse5	✓	✓	x	-4.56	*Type eB*	*Type gH*
1981390	Mouse6	✓	x	✓	-4.28	*Type eN*	x
198140	Mouse6	✓	✓	x	-4.28	*Type eN*	*Type gC*
198141	Mouse6	✓	✓	x	-4.28	*Type eB*	*Type gH*
198242	Mouse6	✓	x	✓	-4.56	*Type eN*	x
198243	Mouse6	✓	✓	x	-4.56	*Type eN*	*Type gG*
198344	Mouse6	✓	✓	x	-4.72	*Type eN*	*Type gC*
198345	Mouse6	x	✓	x	-4.72	N/A	*Type gC*
198548	Mouse7	✓	x	✓	-4.44	*Type eN*	x
198549	Mouse7	✓	x	✓	-4.44	*Type eN*	x
198650	Mouse8	✓	✓	x	-4.56	*Type eB*	*Type gG*
198751	Mouse8	✓	x	x	-4.12	*Type eB*	x
198752	Mouse8	✓	x	x	-4.12	*Type eB*	x
198753	Mouse8	✓	x	✓	-4.12	*Type eB*	x
198754	Mouse8	✓	✓	x	-4.12	*Type eB*	*Type gG*
198855	Mouse8	✓	✓	x	-4.56	*Type eB*	*Type gH*
198856	Mouse8	✓	x	x	-4.56	*Type eN*	x
198957	Mouse8	✓	✓	x	-3.96	*Type eN*	*Type gC*
198958	Mouse8	✓	✓	x	-3.96	*Type eN*	*Type gC*
199273	Mouse9	✓	✓	x	-4.28	*Type eB*	*Type gH*
199274	Mouse9	✓	x	x	-4.28	*Type eB*	x

continued on next page…

MRR neuron ID	Mouse ID	*In vitro* data	Genetic data	Cell reconstruction	Map localization (DV Br.)	Electrophysiology category	Genetic category
199375	Mouse9	✓	x	x	-3.96	*Type eB*	x
199376	Mouse9	✓	x	x	-3.96	*Type eB*	*Type gH*
199377	Mouse9	✓	x	x	-3.96	*Type eB*	x
199378	Mouse9	✓	x	x	-3.96	*Type eB*	x
199379	Mouse9	✓	x	x	-3.96	*Type eB*	x
199480	Mouse9	✓	x	x	-4.72	*Type eN*	*Type gG*
199481	Mouse9	✓	x	✓	-4.72	*Type eN*	x
199482	Mouse9	✓	✓	✓	-4.72	*Type eN*	*Type gG*
199483	Mouse9	✓	x	✓	-4.72	*Type eN*	x
199584	Mouse10	✓	✓	✓	-4.12	*Type eB*	*Type gH*
199585	Mouse10	✓	x	✓	-4.12	*Type eB*	x
199586	Mouse10	✓	✓	x	-4.12	*Type eN*	*Type gG*
199587	Mouse10	✓	x	x	-4.12	*Type eB*	x
199688	Mouse10	✓	✓	✓	-4.44	*Type eN*	*Type gG*
199689	Mouse10	✓	✓	x	-4.44	*Type eN*	*Type gC*
199690	Mouse10	✓	x	✓	-4.44	*Type eN*	x
199691	Mouse10	✓	x	✓	-4.44	*Type eN*	x
199792	Mouse10	✓	x	x	-4.56	*Type eB*	x
199793	Mouse10	✓	✓	✓	-4.56	*Type eN*	*Type gG*
199794	Mouse10	✓	✓	✓	-4.56	*Type eN*	*Type gG*
199895	Mouse10	✓	x	✓	-4.56	*Type eN*	x
199896	Mouse10	✓	x	✓	-4.56	*Type eN*	x
199897	Mouse10	✓	✓	✓	-4.56	*Type eN*	*Type gC*
2003113	Mouse11	✓	✓	✓	-4.72	*Type eB*	*Type gG*
2003114	Mouse11	✓	x	x	-4.72	*Type eB*	x
2003115	Mouse11	✓	x	✓	-4.72	*Type eB*	x
2004116	Mouse11	✓	✓	x	-4.44	*Type eN*	*Type gC*
2004117	Mouse11	✓	✓	x	-4.44	*Type eN*	*Type gG*
2004118	Mouse11	✓	✓	x	-4.44	*Type eN*	*Type gG*
2004119	Mouse11	✓	x	✓	-4.44	*Type eN*	x
2004122	Mouse11	✓	✓	✓	-4.44	*Type eN*	*Type gG*
2004123	Mouse11	✓	x	x	-4.44	*Type eN*	x
2004124	Mouse11	✓	✓	x	-4.44	*Type eN*	*Type gC*
2005126	Mouse11	✓	✓	✓	-4.72	*Type eN*	*Type gG*
2005127	Mouse11	✓	x	x	-4.72	*Type eB*	x
2006128	Mouse11	✓	x	x	-4.56	*Type eB*	x
2006129	Mouse11	✓	x	x	-4.56	*Type eB*	x
2006130	Mouse11	✓	x	x	-4.56	*Type eN*	x
2006131	Mouse11	✓	✓	x	-4.56	*Type eN*	*Type gC*
2007132	Mouse12	✓	✓	x	-4.56	*Type eN*	*Type gC*
2007133	Mouse12	✓	x	x	-4.56	*Type eB*	x
2007134	Mouse12	✓	✓	x	-4.56	*Type eN*	*Type gG*
2008136	Mouse12	✓	x	✓	-4.12	*Type eB*	x
