# Supplementary material for: Prefrontal-raphe-habenular circuit drives fear memory recall and updating
Source: Nat Commun. 2026 Aug 12;17:9686. doi: 10.1038/s41467-026-76574-5 (PMC13558683; doi:10.1038/s41467-026-76574-5)
Supplement: Supplementary file 2 [file 41467_2026_76574_MOESM2_ESM.pdf]

nature portfolio

Peer Review File

Prefrontal-raphe-habenular circuit drives fear memory recall and updating

Corresponding Author: Professor Gabor Nyiri

This file contains all reviewer reports in order by version, followed by all author rebuttals in order by version.

Version 0:

Reviewer comments:

Reviewer #1

(Remarks to the Author)
Based on their previous work, the authors further explore the role of MRRvGluT2 neurons in modulation of fear memory recall and depression. They demonstrated that the inhibiting MRRvGluT2 neurons reduced fear expression. Using in vivo recordings, they found that MRRvGluT2 neurons were activated during fear memory recall. Furthermore, the authors provide evidence that both the mPFC→MRR and MRRvGluT2→LHb pathways respond to the auditory conditioned stimulus (CS) after fear conditioning, and that inhibition of these pathways reduces fear behavior. The study proposes a tri-synaptic mPFC→MRRvGluT2→LHb pathway as a key pathway modulating fear memory recall. Beyond fear regulation, the authors also implicate MRRvGluT2 neurons in depression-like behaviors and suggest a potential role in mediating ketamine's antidepressant effects. Overall, the study addresses an interesting and integrative research question and employs a diverse set of techniques for circuit dissection. However, several issues should be addressed before the manuscript can be considered suitable for publication:

1. Please check the reported number of mice (n-values) in Fig. 1K, M, N, O, P, and Fig. S1C. Using Fig. 1O as an example: on Day 9, the same cohort of mice was first tested in Context A and then in Context B. However, the n-value reported for these two tests is different. Could you please clarify if this is an error, or if some data points were excluded between the tests? Additionally, please verify the n-values across all the aforementioned panels and others.

2. For the in vivo recordings during the fear memory recall test, the study showed that the activity of MRRvGluT2 neurons exhibits a significantly decreasing trend across the five cue presentations. A question is whether the magnitude of this neural activity during each discrete cue presentation is correlated with the concurrent fear-like behavior (e.g., freezing levels).

3. The study demonstrates that inhibition of MRRvGluT2→LHb neurons reduces firing in a subset of LHb neurons and decreases pupil diameter in head-fixed mice. However, several important questions remain:
a) Did the inhibition suppress the activity of LHb neurons that were activated by the CS during fear memory recall?

b) While pupil diameter is a useful fear metric, it remains unclear whether the fear-reducing effect of inhibiting MRRvGluT2→LHb neurons can be replicated in freely moving mice using standard freezing behavior assays. Confirming this effect in a more natural behavioral paradigm would significantly enhance the validity of the findings.

c) Given that MRRvGluT2→LHb neurons also project to multiple brain regions involved in memory and emotional regulation, it is important to consider whether the observed effects are mediated through the LHb pathway or may involve collateral projections to other regions. To unequivocally establish the role of the LHb projection, the authors could inhibit the terminals of MRRvGluT2 neurons specifically within the LHb. This approach would help rule out contributions from other downstream targets and clarify the neural pathway underlying the behavioral effects.

4. It would significantly strengthen the study if the authors could employ a consistent experimental paradigm to investigate the neural circuit mechanisms underlying behavioral phenotypes across both fear and depression assays. It would minimize interpretational confounds arising from paradigm heterogeneity and strengthen the causal coherence between manipulation and functional outcomes. For instance, while contextual/auditory fear conditioning was used to probe MRRvGluT2 neurons function in fear, a head-fixed setup was introduced to probe MRRvGluT2-LHb pathway function. Similarly, for depression-related assays, chronic corticosterone administration was applied to monitor activity changes in MRRvGluT2 neurons, while

repeated foot-shock stress was used to assess the behavioral effects of manipulating these neurons.

5. The analysis in Fig. 3I should carefully account for potential post-inhibitory rebound activation following the cessation of light stimulation, especially in those trials where inhibition was initially conducted.

6. The study suggests that MRRvGluT2 neurons contribute to depression-like behaviors, as demonstrated using mouse models of chronic corticosterone administration and a protocol of unpredictable mild stress that included repeated foot-shocks. Evaluating whether the reported results can be replicated in more naturalistic and widely accepted depression models, such as chronic social defeat stress (CSDS) or a chronic unpredictable mild stress (CUMS) regimen, would significantly enhance the validity of the findings.

7. The study demonstrates that ketamine reduces the activity of MRRvGluT2 neurons， however, it remains unclear whether this effect is directly mediated through actions on MRRvGluT2 neurons themselves, or indirectly via ketamine's influence on LHb neuronal activity.

8. The study demonstrates that chronic chemogenetic inhibition of MRRvGluT2 neurons alleviates depression-like behaviors. Given that even acute inhibition of these neurons produces a lasting reduction in fear behavior, it would be interesting to examine whether an acute inhibition of MRRvGluT2 neurons is also sufficient to elicit sustained antidepressant-like effects.

9. The study demonstrates that inhibition of the MRRvGluT2 neurons, MRR→LHb and mPFC→MRR pathways reduces fear-like behavior. However, to ensure that these behavioral effects are not confounded by alterations in general locomotor activity, additional control experiments are necessary.

10. To accurately evaluate the specificity and placement of viral expression and optic fiber implantation, it is better to include images of the entire coronal section(s) rather than cropped or zoomed-in views.

11. The authors state that their findings "clearly suggest that MRRvGluT2 neurons are vital for reinforcing the aversive valence of the recalled memory" (Page 15, Lines 11–14). To improve clarity, it would be helpful to further explain what is meant by "reinforcing the aversive valence of the recalled memory" in this context.

12. The authors wrote "Activity of mPFC→MRR neurons scales with the aversiveness of negative experiences" in Fig 8; and "we demonstrated that the activity of MRRvGluT2 neurons scale with the aversiveness of the memory" in discussion (Page 15, line16-18). To strengthen the interpretation, the authors should explicitly define the term "aversiveness" and clarify how it was quantified or inferred.

13. A question arising from this study concerns the functional differentiation between the proposed mPFC→MRR pathway and other mPFC efferent pathways—such as those targeting the amygdala, paraventricular thalamus, periaqueductal gray, and so on—in the regulation of fear memory recall.

Reviewer #2

(Remarks to the Author)
Synopsis
With recent literature, there came increasing emphasis on characterizing fear circuit components beyond classical amygdala pathways. This paper is a welcome addition, as it identifies the PFC–MRR–LHb circuit as a critical pathway for fear recall and integrates this into a more holistic view of the brain's fear systems.
Initiated by their previous study on MRR(vGlut2) in fear learning (Szönyi et al. 2019), the authors expand on these findings by investigating (i) its embedding within canonical fear circuits and (ii) its role in fear recall. The authors first examined the specific role of the MRRvGlut2 system in fear recall. In a comprehensive set of experiments, opto-silencing of MRRvGlut2 neurons during recall reduced contextual fear expression (Fig. 1A–D). The same manipulation also led to a significant reduction of cued fear memory (Fig. 1E–H), demonstrating a role for these neurons in fear recall and memory maintenance. These effects generalized (Fig. 1K) and extended to compound fear memories (Fig. 1L–P). To mechanistically support these findings, the authors next performed calcium fiber photometry throughout their cued fear assays. Indeed, MRRvGlut2 neurons were reactivated during fear recall (Fig. 2A–C), but strikingly, not as strongly during fear learning. These results were confirmed by head-fixed electrophysiological recordings, which showed activity both during learning and recall (Fig. 2D–E), decreasing (extinguishing) over sessions. The authors suggest that this reflects activity scaling with fear levels. To understand how MRRvGlut2 neurons contribute to fear recall, the authors extended their previous mapping (Szönyi et al. 2019), providing a more comprehensive analysis of cortical and subcortical affective areas. They highlighted the LHb as one of the most prominent outputs (Fig. S3A–C), confirming their earlier results. Importantly, they demonstrate that individual neurons broadcast signals to multiple emotion- and memory-related centers simultaneously (Fig. S3D–F). Focusing on MRRvGlut2 projections in fear recall, they employed projection-specific retrograde opto-silencing in both head-fixed and freely moving experiments. Indeed, these data demonstrated that the MRR→LHb projection controls LHb activity and fear states during recall (Fig. 3B–K). To relate MRRvGlut2 activity to chronic stress, a known modulator of fear, the authors investigated MRRvGlut2 function under CORT-induced stress and subsequent ketamine treatment. CORT increased c-fos activity in MRRvGlut2 cells and induced anhedonia, effects that lasted over several days but were reversed by ketamine (Fig. 4A–D). Consistently, chemogenetic inhibition of MRRvGlut2 neurons reduced forced-stress (FS)-induced depression-like symptoms in the sucrose preference test (SPT; Fig. 4G–I). Given the broader projection pattern and

translational potential of MRRvGlut2 neurons in controlling diverse aversive states, the authors then explored their potential heterogeneity. Patch-clamp profiling revealed two major electrophysiological subtypes (Fig. 5B–D), and genetic profiling of MRRvGlut2-LHb neurons identified three molecular subtypes (Fig. S6–S8). Combining genetic, electrophysiological, and histological analyses (Fig. 6A–E), they identified PV-expressing excitatory neurons as the most numerous and translationally relevant group in this context. These findings were further strengthened by comparative data across mouse, monkey, and human brains (Fig. 6G–J), suggesting an evolutionarily conserved function with translational potential. Finally, the study investigated how MRRvGlut2 neurons embed into canonical fear circuitry. Their connectivity with the PFC proved most striking: anatomical tracing revealed strong direct inputs from ACC, PL, and IL cortex (Fig. 7G), suggesting transmission of fear signals. Optogenetic activation of these projections produced strong negative valence and real-time place aversion (Fig. 7L–N), and this activity was persistently and adaptively recruited by aversive stimuli (Fig. 8A–L). Conversely, optogenetic inhibition of mPFC→MRR projections prevented contextual recall and extinction (Fig. 9A–E). In line with this, mPFC–MRR projection neurons tagged during fear learning were reactivated at recall. Combined anterograde/retrograde viral tracing and electron microscopy further demonstrated strong afferents from hippocampus and BLA (Fig. 10A–I), consistent with transfer of contextual and cued fear information into the mPFC–MRR circuitry during recall. Overall, the authors explore and add to the understanding of non-canonical fear circuits in an extensive study with well-conducted and transparently reported experiments. The work is synthesized into a comprehensive updated working model of fear circuitry (Fig. S17). However, as I outline below, the manuscript requires revisions to increase its accessibility and impact for a broader readership.

Major Comments
For readers not familiar with the topic, it is sometimes difficult to appreciate the conceptual advance over Szönyi et al. (2019), other than the focus on recall (instead of fear learning) and expanded circuitry. The authors should better explain why a specific focus on fear learning (Szönyi et al. 2019) and fear recall is needed, and what the reader can learn from this. This is particularly important since the current study does not compare learning vs. recall throughout to investigate overlap and dissociation between these functions.
Statistical analysis has to be reworked to adjust for multiple comparisons, whenever appropriate. E.g. Kruskal-Wallis test is not reported and Mann-Whitney U-test is not corrected for multiple comparison (e.g. Fig.1 and fig.S1, Fig. 2C I, J). The rationale for sometimes performing multiple comparison correction (e.g.figS8K) and sometimes not (e.g. Fig. 4H) is unclear. Overall, multiple comparisons have to be adjusted for throughout the manuscript.
Fig.1: Mouse N alternate between Control/ArchT=21/16 and 15/11. It should be made clearer in the text, which cohort underwent which experiment. E.g. from the text (line12-16) it seems the data in Fig.H/I and J/K is derived from the same cohort, however J/K animal N matches Fig. 1 C/D instead. Furthermore, in O/P animal N changes again and is difficult to understand, since exposure to Context A and B was not done on the same (set of) animals. Moreover, Context B ArchT N is too low to argue with absence of significance (P4|Line 34-36).
The authors suggest that MRRvGlut2 neurons (Fig. 2) and PFC–MRR neurons (Figs. 8 and 9) scale with fear memory recall. However, the evidence for scalable representation is rather weak. I strongly suggest attempting to correlate the activity data with actual behavioral expression of fear, perhaps on an animal-by-animal basis.
It is unclear to this reviewer why the demonstrations of stress recruitment (Figs. 4A–D) and stress antagonism (Figs. 4E–H) are performed in different experimental settings. At the very least, a clear rationale should be provided.
Fig.4I: The effect size in this assay seems quite modest to clearly demonstrate an anhedonic state. Similarly, the CORT effect in FigS4B seems to rest on 3 animals and is not controlled for multiple comparisons. Collectively, the data showing a depression-like phenotype should be strengthened by increasing the N and more robust statistical analysis.
While generally easy to read, the overall narrative and message could be more effective by reworking the rationales for transitions between sections of the Results. This reviewer became lost at points, as some statements lack precision.
The deeper histological assessment of MRRvGlut2 brain-wide connectivity compared to Szönyi et al. (2019) (Fig. S3) should include, at the very least, a graphed quantitative assessment of the different projections.
To my understanding, the negative state induced by mPFC–MRR activation (Figs. 7L–N) and by MRRvGlut2 activation (Figs. S1D–G) are demonstrated in different assays, i.e., place preference vs. auditory conditioning. The rationale for this design is missing.

Minor Comments
The phrase “but the circuit described in the literature was incomplete” suggests that the authors now provide a complete description. This should be toned down to avoid overstatement.
FigS1A: Absence of significance at conditioning seems to be driven by an outlier. Otherwise, conditioning data seems comparable to Recall with manipulation. This undermines the reported effect at Recall (Fig. 1D).
Fig. 3F-L: What is the rationale for inhibiting only half of the trial? Why not every other trial?
P7|Line 20-21: How does this fit to the fact that MRRvGlut2 neuron activity fades after 3 trials (Fig.2I)? Is the cellular activity reactivated?
Quantifications in Fig. 6 would be more accessible in a figure than in the legend.
Behavior for conditioning (e.g. Fig.9D-E, day 6) should be reported throughout the paper.

Reviewer #3

(Remarks to the Author)
The authors provided the impressive amount of data collected using a diverse range of neuroscientific techniques. However, despite this substantial effort, the manuscript's central narrative is unclear. It is difficult for the reader to distill a core takeaway message from the extensive results presented. This lack of focus is further exemplified by the inclusion of numerous figures that do not seem to directly align with the main theme promised by the title.

A clear example of this disjointed narrative is found in Figure 4, which illustrates the function of MRR in a depression model. The manuscript fails to clarify the relevance of this finding to fear memory processing, which is the study's central theme. The authors should explicitly state and justify the connection between these two contexts.
In addition, the authors propose that the prefrontal-raphe-habenular circuit drives fear memory recall. While figure 1 and 2 show that inactivation of the MRR impairs fear memory recall, subsequent figures focus on the role of raphe-habenular circuit in depression-like behaviors or the involvement of mPFC-raphe projections in encoding negative valence. These data suggest that the observed fear memory recall impairments in figure 1, 2 may reflect disruptions in fear expression or aversive assessment, rather than memory recall per se. However, the schematic in figure S17 implies that MRR is involved in neither memory-related nor aversion-related process, further adding to the conceptual ambiguity. This lack of clear mechanistic framing weakens the overall coherence and significance of the manuscript's central argument.

Fundamentally, while the central premise of the study—that MRR is crucial for fear memory recall and the effort to identify its circuits—is novel and holds potential, the manuscript as presented does not convincingly argue that the findings represent a significant conceptual advance worthy of publication in Nature Communications.
To be clear, this is not a critique of the data's quality, quantity, or the authors' commendable efforts. Rather, the issue lies with the manuscript itself, which fails to effectively frame the findings and articulate their broader impact. The paper currently reads more like a collection of interesting observations rather than a cohesive narrative that builds a compelling case for the significance of this circuit.
For these reasons, I cannot recommend the manuscript for publication in Nature Communications in its current form. However, should the authors undertake a substantial revision to build a more focused and compelling narrative around their most significant findings, the work could represent a valuable contribution to the field.

Reviewer #4

(Remarks to the Author)
I co-reviewed this manuscript with one of the reviewers who provided the listed reports. This is part of the Nature Communications initiative to facilitate training in peer review and to provide appropriate recognition for Early Career Researchers who co-review manuscripts.

Version 1:

Reviewer comments:

Reviewer #1

(Remarks to the Author)
The authors have addressed the points raised. The work is now suitable for publication in Nature Communications.

Reviewer #2

(Remarks to the Author)
This reviewer would like to thank the authors for their comprehensive revision, clarifying their view on statistical matters, improving accessibility, and strengthening the conceptual framework, as well as including additional datasets. Specifically, the additional statements on learning vs. recall help the reader better understand the perspective and novelty of the study (Introduction). Also, the authors have now added correlation data to resolve the scalability of their effects more comprehensively (Figs. 1, 2, S2).
On a final note, I believe that applying the same matrix of experiments across the study consistently creates added value. In particular, when probing MRRvGluT2 in memory generation (Fig. S1) vs. mPFC→MRR in inducing an aversive state (Fig. 7): performing both behaviors on both circuit elements would dissociate these effects directly. That said, I understand that this might go beyond the scope of the study at this point.
Taken together, I am very much looking forward to seeing this paper published.

Reviewer #3

(Remarks to the Author)
The authors addressed my concerns adequately.

Reviewer #4

(Remarks to the Author)
I co-reviewed this manuscript with one of the reviewers who provided the listed reports. This is part of the Nature Communications initiative to facilitate training in peer review and to provide appropriate recognition for Early Career Researchers who co-review manuscripts.

Open Access This Peer Review File is licensed under a Creative Commons Attribution 4.0 International License, which permits use, sharing, adaptation, distribution and reproduction in any medium or format, as long as you give appropriate credit to the original author(s) and the source, provide a link to the Creative Commons license, and indicate if changes were made.
In cases where reviewers are anonymous, credit should be given to 'Anonymous Referee' and the source.
The images or other third party material in this Peer Review File are included in the article's Creative Commons license, unless indicated otherwise in a credit line to the material. If material is not included in the article's Creative Commons license and your intended use is not permitted by statutory regulation or exceeds the permitted use, you will need to obtain permission directly from the copyright holder.
To view a copy of this license, visit https://creativecommons.org/licenses/by/4.0/

Response to comments by the Reviewers

We thank the Reviewers for their constructive comments and suggestions, and we were pleased to receive their positive reviews of our manuscript. Here we present the Reviewers' unaltered comments and our point-by-point responses (in blue text). In the revised manuscript, we presented additional supporting experimental evidence, and we indicated further changes in blue text that are related to the Reviewers' suggestions.

Reviewer #1 (Remarks to the Author):

Based on their previous work, the authors further explore the role of MRRvGluT2 neurons in modulation of fear memory recall and depression. They demonstrated that the inhibiting MRRvGluT2 neurons reduced fear expression. Using in vivo recordings, they found that MRRvGluT2 neurons were activated during fear memory recall. Furthermore, the authors provide evidence that both the mPFC→MRR and MRRvGluT2→LHb pathways respond to the auditory conditioned stimulus (CS) after fear conditioning, and that inhibition of these pathways reduces fear behavior. The study proposes a tri-synaptic mPFC→MRRvGluT2→LHb pathway as a key pathway modulating fear memory recall. Beyond fear regulation, the authors also implicate MRRvGluT2 neurons in depression-like behaviors and suggest a potential role in mediating ketamine's antidepressant effects. Overall, the study addresses an interesting and integrative research question and employs a diverse set of techniques for circuit dissection. However, several issues should be addressed before the manuscript can be considered suitable for publication:

We thank the reviewer for their thoughtful and comprehensive summary of our work. Please find our point-by-point responses below.

1. Please check the reported number of mice (n-values) in Fig. 1K, M, N, O, P, and Fig. S1C. Using Fig. 1O as an example: on Day 9, the same cohort of mice was first tested in Context A and then in Context B. However, the n-value reported for these two tests is different. Could you please clarify if this is an error, or if some data points were excluded between the tests? Additionally, please verify the n-values across all the aforementioned panels and others.

We thank the reviewer for pointing this out. We carefully rechecked all n-values for all figures and we corrected typos accordingly. The n-values reported for Fig 1 O-P are different because we tested multiple cohorts within the same test, but some initial cohorts did not participate in the final test (Fig 1P context B). This is now clarified in the Methods/Fear conditioning test 3.

2. For the in vivo recordings during the fear memory recall test, the study showed that the activity of MRRvGluT2 neurons exhibits a significantly decreasing trend across the five cue presentations. A question is whether the magnitude of this neural activity during each discrete cue presentation is correlated with the concurrent fear-like behavior (e.g., freezing levels).

During cued fear conditioning experiments (Fig 1), as expected, we observed the well-known decline in freezing levels during fear extinction. Then, in our fiber photometry experiments, we observed a significant negative regression between the Ca^{2+}-dependent fluorescence signal (activity of MRR^{vGluT2} neurons) and the consecutive cue presentations (Fig2C, FigS2B) during fear recall, but not

in the isosbestic control signal. Although, in that particular experiment, the sample size is insufficient to show a direct correlation on an "animal-by-animal" basis, we did find a significant negative regression of baseline-corrected freezing levels across the consecutive cue presentations in these experiments as well (FigS2C), along with the decline of the activity of MRR^{vGluT2} neurons. The Results and the supplementary figure S2 were updated with this data accordingly.

3. The study demonstrates that inhibition of MRRvGluT2→LHb neurons reduces firing in a subset of LHb neurons and decreases pupil diameter in head-fixed mice. However, several important questions remain: a) Did the inhibition suppress the activity of LHb neurons that were activated by the CS during fear memory recall?

As it is expected based on literature data (see Fig3 of Congiu et al., Mol Psych, 2023), out of 153 identified LHb units, only 33 increased their activity upon CS presentation. Nine of these units (9/33, 27.3%) decreased, while the other units did alter their firing rates upon optogenetic inhibition of MRR$^{vGluT2\rightarrow LHb}$ neurons during CS presentation. The Results text was updated accordingly.

b) While pupil diameter is a useful fear metric, it remains unclear whether the fear-reducing effect of inhibiting MRRvGluT2→LHb neurons can be replicated in freely moving mice using standard freezing behavior assays. Confirming this effect in a more natural behavioral paradigm would significantly enhance the validity of the findings. c) Given that MRRvGluT2→LHb neurons also project to multiple brain regions involved in memory and emotional regulation, it is important to consider whether the observed effects are mediated through the LHb pathway or may involve collateral projections to other regions. To unequivocally establish the role of the LHb projection, the authors could inhibit the terminals of MRRvGluT2 neurons specifically within the LHb. This approach would help rule out contributions from other downstream targets and clarify the neural pathway underlying the behavioral effects.

In response to these questions, we conducted an additional contextual fear conditioning experiment combined with optogenetic inhibition of MRR$^{vGluT2\rightarrow LHb}$ axons in the LHb during fear recall. This manipulation resulted in significantly enhanced fear extinction, while replicating our findings in freely moving mice using standard freezing behavior assays, thereby unequivocally established the role of the LHb projection, as requested by this reviewer. We demonstrated this new data in a new Results section, along with the relevant figures (Fig3N-R, FigS4H-K) and tables, which further enhance the validity of our findings.

4. It would significantly strengthen the study if the authors could employ a consistent experimental paradigm to investigate the neural circuit mechanisms underlying behavioral phenotypes across both fear and depression assays. It would minimize interpretational confounds arising from paradigm heterogeneity and strengthen the causal coherence between manipulation and functional outcomes. For instance, while contextual/auditory fear conditioning was used to probe MRRvGluT2 neurons function in fear, a head-fixed setup was introduced to probe MRRvGluT2-LHb pathway function. Similarly, for depression-related assays, chronic corticosterone administration was applied to monitor activity changes in MRRvGluT2 neurons, while repeated foot-shock stress was used to assess the behavioral effects of manipulating these neurons.

Different experimental paradigms were deliberately chosen to probe distinct aspects of the same mPFC→MRR→LHb circuitry using the most sensitive approaches. The most effective experiments for a given question enabled the most efficient reduction in animal use. Freely moving paradigms were required for fear conditioning and freezing behavior assessment, whereas head-fixed

preparations enabled pathway-specific physiological measurements not feasible in freely moving animals. For instance, our new freely moving experiments further supported our conclusion by demonstrating that the MRR^{vGluT2}→LHb pathway contributes to fear recall not only in head-fixed conditions, but also in freely-moving contextual fear memory recall. Similarly, chronic corticosterone exposure and repeated foot-shock stress were used to examine complementary aspects of depression-related phenotypes. Importantly, the convergence of findings across paradigms demonstrates that the observed effects are robust and not dependent on a specific experimental setting.

5. The analysis in Fig. 3I should carefully account for potential post-inhibitory rebound activation following the cessation of light stimulation, especially in those trials where inhibition was initially conducted.

Indeed, a brief rebound activation could potentially influence the results, however only when inhibition occurred during the first 10 s of the cue presentation. However, we found that they did not affect the conclusions. Specifically, neuronal activity during the 10 s no-light periods did not differ depending on whether these periods occurred in the first or second half of the cue presentation (z-scored firing rate, first half: 0.062 [−0.007, 0.167]; second half: 0.068 [0.014, 0.113]; Wilcoxon signed-rank test, $p = 0.83$). In addition, the reduction in firing rate did not differ between the two paradigms (first half: -0.150 [-0.231,-0.089], second half: -0.127 [-0.218,-0.061]; Wilcoxon signed rank test; $p=0.27$). The figure legend and the Methods section have been updated accordingly.

6. The study suggests that MRRvGluT2 neurons contribute to depression-like behaviors, as demonstrated using mouse models of chronic corticosterone administration and a protocol of unpredictable mild stress that included repeated foot-shocks. Evaluating whether the reported results can be replicated in more naturalistic and widely accepted depression models, such as chronic social defeat stress (CSDS) or a chronic unpredictable mild stress (CUMS) regimen, would significantly enhance the validity of the findings.

In the present study, our aim was to establish a causal role for MRR^{vGluT2} neurons using two mechanistically distinct and widely accepted stress-related models. We have chosen a chronic corticosterone administration and an unpredictable mild stress paradigm. Importantly, these independent approaches converged on the same conclusion and provided strong evidence that MRR^{vGluT2} neuronal activity is involved in stress-induced depression-related states. While future studies using additional models will be valuable, the current findings already establish a robust and causal link between this circuit and depression-related phenotypes as well.

7. The study demonstrates that ketamine reduces the activity of MRRvGluT2 neurons, however, it remains unclear whether this effect is directly mediated through actions on MRRvGluT2 neurons themselves, or indirectly via ketamine's influence on LHb neuronal activity.

We agree that ketamine may act through both direct and indirect mechanisms. It may directly influence MRR^{vGluT2} neurons and may also modulate them indirectly through ketamine-sensitive LHb neurons that provide input to the MRR (as we described previously). Although our data do not define the primary site of ketamine action, they show that MRR^{vGluT2} neurons play a role in this process, which is in line with our behavioral data as well.

8. The study demonstrates that chronic chemogenetic inhibition of MRR^{vGluT2} neurons alleviates depression-like behaviors. Given that even acute inhibition of these neurons produces a lasting reduction in fear behavior, it would be interesting to examine whether an

acute inhibition of MRR^{vGluT2} neurons is also sufficient to elicit sustained antidepressant-like effects.

Determining the shortest and least intense modulation of MRR^{vGluT2} neurons that is sufficient to elicit behavioral effects is an interesting question; however, addressing it would require substantial additional resources. Our current data demonstrate that chronic modulation of MRR^{vGluT2} neurons is sufficient to bidirectionally influence depression-related behaviors, thereby providing a solid foundation for future studies to investigate the precise temporal and dosage parameters underlying these effects.

9. The study demonstrates that inhibition of the MRR^{vGluT2} neurons, MRR→LHb and mPFC→MRR pathways reduces fear-like behavior. However, to ensure that these behavioral effects are not confounded by alterations in general locomotor activity, additional control experiments are necessary.

Increased locomotor activity could, in principle, contribute to reduced acute freezing behavior. However, several lines of evidence argue against this interpretation.
First, and most importantly, the effect of MRR^{vGluT2} neuronal inhibition was not restricted to the light-on period. Reduced freezing persisted on day 9, when no light was delivered (Fig 1D, 1H-I, 1O-P, 3R), indicating that the manipulation altered fear expression and memory rather than producing an acute locomotor effect.
Second, MRR^{vGluT2} neurons primarily project to regions such as the LHb and ventral tegmental areas, which are strongly implicated in aversive and motivational state regulation rather than direct motor control. Moreover, manipulations of both their inputs and outputs produced similar effects. Thus, movement-related changes are more likely to reflect altered affective state than a primary locomotor effect.
Third, our previous work showed that stimulation of MRR^{vGluT2} neurons increases movement speed, making it unlikely that inhibition would produce the same locomotor effect (Szőnyi et al., Science, 2019; Fig. 8A–D).
Fourth, independent recent work showed that stimulation of MRR^{vGluT2} neurons increases exploratory behavior, whereas inhibition does not alter exploratory behavior (Ahmadlou et al., Nature, 2025; Fig. 2a,e).
Finally, a direct locomotor effect would be expected to occur rapidly during light exposure, on a seconds-long timescale (Fig8 A-D of Szőnyi et al., Science 2019 and Fig4g of Tovote et al. Nature 2016). We therefore performed an additional analysis of animal locomotion during MRR^{vGluT2} neuronal inhibition in the contextual fear conditioning experiment. We found no difference in locomotor activity between CTRL and ArchT mice when comparing 10 s periods before and after starting light exposure.
Together, these findings indicate that reduced freezing is consistent with reduced fear expression. The manuscript and tables have been updated accordingly (Fig S1B-C).

10. To accurately evaluate the specificity and placement of viral expression and optic fiber implantation, it is better to include images of the entire coronal section(s) rather than cropped or zoomed-in views.

We carefully evaluated all viral injection sites and optic fiber placements across several hundreds of animals, and only included data from animals with accurate targeting, as summarized in Fig. S16, where optic fibre positions are demonstrated. While we agree that full coronal sections can be informative, presenting them in the main figures is not practical in this case, as the MRR occupies a very small portion of the coronal plane. In full-section images, the precise position of the optic fibers - our primary point of validation - would not be clearly visible. We therefore opted for higher-magnification views to ensure accurate visualization of targeting.

11. The authors state that their findings "clearly suggest that MRRvGluT2 neurons are vital for reinforcing the aversive valence of the recalled memory" (Page 15, Lines 11–14). To improve clarity, it would be helpful to further explain what is meant by "reinforcing the aversive valence of the recalled memory" in this context.

We thank the reviewer for pointing out this ambiguity. By "reinforcing the aversive valence of the recalled memory," we mean that MRR^{vGluT2} neurons are required to re-engage the negative emotional component previously associated with the experience, thereby reinstating the affective state that accompanies fear memory recall. This interpretation is supported by our finding that MRR^{vGluT2} neurons are necessary for both contextual and cued fear memory recall and for maintaining fear responses during extinction. We have now clarified this at several point in the revised manuscript and described it in more details in the Discussions.

12. The authors wrote "Activity of mPFC→MRR neurons scales with the aversiveness of negative experiences" in Fig 8; and "we demonstrated that the activity of MRRvGluT2 neurons scale with the aversiveness of the memory" in discussion (Page 15, line16-18). To strengthen the interpretation, the authors should explicitly define the term "aversiveness" and clarify how it was quantified or inferred.

Thank you for this request. Aversiveness refers to the degree to which a stimulus, event, or experience is unpleasant or negatively valued. To improve clarity, we have now updated the text accordingly. Aversiveness was inferred from behavioral and physiological indicators, including freezing behavior, pupil dilation.

13. A question arising from this study concerns the functional differentiation between the proposed mPFC→MRR pathway and other mPFC efferent pathways—such as those targeting the amygdala, paraventricular thalamus, periaqueductal gray, and so on—in the regulation of fear memory recall.

We agree that distinct mPFC efferent pathways are likely to serve different functions. Our results specifically identify the mPFC→MRR pathway as contributing to recalled fear, without addressing the roles of other mPFC projections.

Reviewer #2 (Remarks to the Author):

Synopsis
With recent literature, there came increasing emphasis on characterizing fear circuit components beyond classical amygdala pathways. This paper is a welcome addition, as it identifies the PFC–MRR–LHb circuit as a critical pathway for fear recall and integrates this into a more holistic view of the brain's fear systems.
Initiated by their previous study on MRR(vGlut2) in fear learning (Szönyi et al. 2019), the authors expand on these findings by investigating (i) its embedding within canonical fear circuits and (ii) its role in fear recall. The authors first examined the specific role of the MRRvGlut2 system in fear recall. In a comprehensive set of experiments, opto-silencing of MRRvGlut2 neurons during recall reduced contextual fear expression (Fig. 1A–D). The same manipulation also led to a significant reduction of cued fear memory (Fig. 1E–H), demonstrating a role for these neurons in fear recall and memory maintenance. These effects generalized (Fig. 1K) and extended to compound fear memories (Fig. 1L–P). To mechanistically support these findings, the authors next performed calcium fiber photometry throughout their cued fear assays. Indeed, MRRvGlut2 neurons were reactivated during fear recall (Fig. 2A–C), but strikingly, not as strongly during fear learning. These results were

confirmed by head-fixed electrophysiological recordings, which showed activity both during learning and recall (Fig. 2D–E), decreasing (extinguishing) over sessions. The authors suggest that this reflects activity scaling with fear levels. To understand how MRRvGlut2 neurons contribute to fear recall, the authors extended their previous mapping (Szönyi et al. 2019), providing a more comprehensive analysis of cortical and subcortical affective areas. They highlighted the LHb as one of the most prominent outputs (Fig. S3A–C), confirming their earlier results. Importantly, they demonstrate that individual neurons broadcast signals to multiple emotion- and memory-related centers simultaneously (Fig. S3D–F). Focusing on MRRvGlut2 projections in fear recall, they employed projection-specific retrograde opto-silencing in both head-fixed and freely moving experiments. Indeed, these data demonstrated that the MRR→LHb projection controls LHb activity and fear states during recall (Fig. 3B–K). To relate MRRvGlut2 activity to chronic stress, a known modulator of fear, the authors investigated MRRvGlut2 function under CORT-induced stress and subsequent ketamine treatment. CORT increased c-fos activity in MRRvGlut2 cells and induced anhedonia, effects that lasted over several days but were reversed by ketamine (Fig. 4A–D). Consistently, chemogenetic inhibition of MRRvGlut2 neurons reduced forced-stress (FS)-induced depression-like symptoms in the sucrose preference test (SPT; Fig. 4G–I). Given the broader projection pattern and translational potential of MRRvGlut2 neurons in controlling diverse aversive states, the authors then explored their potential heterogeneity. Patch-clamp profiling revealed two major electrophysiological subtypes (Fig. 5B–D), and genetic profiling of MRRvGlut2-LHb neurons identified three molecular subtypes (Fig. S6–S8). Combining genetic, electrophysiological, and histological analyses (Fig. 6A–E), they identified PV-expressing excitatory neurons as the most numerous and translationally relevant group in this context. These findings were further strengthened by comparative data across mouse, monkey, and human brains (Fig. 6G–J), suggesting an evolutionarily conserved function with translational potential. Finally, the study investigated how MRRvGlut2 neurons embed into canonical fear circuitry. Their connectivity with the PFC proved most striking: anatomical tracing revealed strong direct inputs from ACC, PL, and IL cortex (Fig. 7G), suggesting transmission of fear signals. Optogenetic activation of these projections produced strong negative valence and real-time place aversion (Fig. 7L–N), and this activity was persistently and adaptively recruited by aversive stimuli (Fig. 8A–L). Conversely, optogenetic inhibition of mPFC→MRR projections prevented contextual recall and extinction (Fig. 9A–E). In line with this, mPFC–MRR projection neurons tagged during fear learning were reactivated at recall. Combined anterograde/retrograde viral tracing and electron microscopy further demonstrated strong afferents from hippocampus and BLA (Fig. 10A–I), consistent with transfer of contextual and cued fear information into the mPFC–MRR circuitry during recall.
Overall, the authors explore and add to the understanding of non-canonical fear circuits in an extensive study with well-conducted and transparently reported experiments. The work is synthesized into a comprehensive updated working model of fear circuitry (Fig. S17). However, as I outline below, the manuscript requires revisions to increase its accessibility and impact for a broader readership.

We thank the reviewer for the thorough and insightful evaluation of our work. We appreciate the recognition of the major discoveries of this study. Please find our point-by-point responses below.

Major Comments

For readers not familiar with the topic, it is sometimes difficult to appreciate the conceptual advance over Szönyi et al. (2019), other than the focus on recall (instead of fear learning) and expanded circuitry. The authors should better explain why a specific focus on fear learning (Szönyi et al. 2019) and fear recall is needed, and what the reader can learn from

this. This is particularly important since the current study does not compare learning vs. recall throughout to investigate overlap and dissociation between these functions.

We thank the reviewer for this insightful comment. The neural mechanisms underlying learning and recall differ substantially. For example, nociceptive processing may be used for learning aversive experiences but not for their recall. Learning requires integration of aversive and contextual/cued information to form a memory, whereas recall requires reactivation and updating of the affective value of an already stored memory. Consistent with this distinction, memory formation and retrieval are supported by distinct neural mechanisms and circuit states, as shown previously e.g. by Roy et al. (Cell, 2017). Our study therefore identifies a previously unrecognized role of the mPFC→MRR→LHb pathway in re-engaging and updating the aversive valence of recalled memories, rather than in encoding the original aversive event. We have revised the manuscript to clarify the conceptual distinction between fear learning and fear recall.

Statistical analysis has to be reworked to adjust for multiple comparisons, whenever appropriate. E.g. Kruskal-Wallis test is not reported and Mann-Whitney U-test is not corrected for multiple comparison (e.g. Fig.1 and fig.S1, Fig. 2C I, J). The rationale for sometimes performing multiple comparison correction (e.g.figS8K) and sometimes not (e.g. Fig. 4H) is unclear. Overall, multiple comparisons have to be adjusted for throughout the manuscript.

We fully agree on the importance of rigorous statistical testing, including adjustments for multiple comparisons, which we teach and advocate whenever appropriate and warranted. In this study, all analyses were selected accordingly. Corrections for multiple comparisons are generally recommended in data-mining approaches or in situations involving numerous undefined post hoc comparisons. However, standard statistical texts state that such corrections are unnecessary and can even be counterproductive when comparisons are defined "a priori" as the central objectives of planned experiments and are reported irrespective of their outcomes (Pagano, Understanding Statistics in the Behavioral Sciences, 9th ed., 2007).
In the case of Fig. S1, no multiple comparisons are present. Likewise, Fig. 1F, G, M, and N do not constitute multiple-comparison problems, as they represent predefined responses to a series of cues; observing consistent significance across these conditions strengthens, rather than weakens the conclusions. Multiple-comparison adjustments are intended to guard against accidentally significant findings, which is clearly not the case here given the repeated confirmation of effects across "a priori" defined tests. In the remaining instances noted by the Reviewer, all comparisons were predefined and all statistical outcomes and values are reported transparently in the Supplementary Data.
By contrast, Fig. S8K represents a typical exploratory ("data-mining") analysis involving post-hoc comparisons of genetic data; accordingly, Bonferroni-corrected p-values were applied in this case.
Finally, we note that non-parametric statistical tests were used throughout the manuscript. These tests are more conservative and generally require much larger effect sizes to reach significance than their parametric counterparts, further supporting the robustness of the reported results.

Fig.1: Mouse N alternate between Control/ArchT=21/16 and 15/11. It should be made clearer in the text, which cohort underwent which experiment. E.g. from the text (line12-16) it seems the data in Fig.H/I and J/K is derived from the same cohort, however J/K animal N matches Fig. 1 C/D instead. Furthermore, in O/P animal N changes again and is difficult to understand, since exposure to Context A and B was not done on the same (set of) animals. Moreover, Context B ArchT N is too low to argue with absence of significance (P4|Line 34-36).

We thank the reviewer for pointing this out. We carefully rechecked all n-values for all figures and we corrected some typos accordingly. The n-values reported for Fig 1 O-P are different because we tested multiple cohorts within the same test, but some initial cohorts did not participate in the final test (Fig 1P context B). This is now clarified in the Methods/Fear conditioning tests 3.
Regarding the number of mice in context B ArchT, the reviewer is right, the phrasing in the text needed to be clarified. Instead of "but not for context B" now we stated that "no such difference could be detected in context B".

The authors suggest that MRRvGlut2 neurons (Fig. 2) and PFC–MRR neurons (Figs. 8 and 9) scale with fear memory recall. However, the evidence for scalable representation is rather weak. I strongly suggest attempting to correlate the activity data with actual behavioral expression of fear, perhaps on an animal-by-animal basis.

During cued fear conditioning experiments (Fig 1), as expected, we observed the well-known decline in freezing levels during fear extinction. Then, in our fiber photometry experiments, we observed a significant negative regression between the Ca^{2+}-dependent fluorescence signal (activity of MRR^{vGluT2} neurons) and the consecutive cue presentations (Fig. 2c) during fear recall, but not in the isosbestic control signal. Although, in that particular experiment, the sample size is insufficient to show a direct correlation on an "animal-by-animal" basis, we did find a significant negative regression between baseline-corrected freezing levels and the consecutive cue presentations in these experiments as well, along with the decline of the activity of MRR^{vGluT2} neurons. The Results and the supplementary Fig S2 were updated with this data accordingly. Regarding the $mPFC^{\rightarrow MRR}$ neurons, scalability is supported by the graded responses of these neurons to sensory stimuli with different levels of aversiveness, rather than by a direct correlation with freezing behavior (Fig. 8L). We have clarified this distinction in the revised manuscript.

It is unclear to this reviewer why the demonstrations of stress recruitment (Figs. 4A–D) and stress antagonism (Figs. 4E–H) are performed in different experimental settings. At the very least, a clear rationale should be provided.

In the present study, our aim was to establish a causal role for MRR^{vGluT2} neurons using two mechanistically distinct and widely accepted stress-related models. We have chosen a chronic corticosterone administration and an unpredictable mild stress paradigm. Importantly, these independent approaches converged on the same conclusion and provided strong evidence that MRR^{vGluT2} neuronal activity is involved in stress-induced depression-related states. While future studies using additional models will be valuable, the current findings already establish a robust and causal link between this circuit and depression-related phenotypes as well.

Fig.4I: The effect size in this assay seems quite modest to clearly demonstrate an anhedonic state. Similarly, the CORT effect in FigS4B seems to rest on 3 animals and is not controlled for multiple comparisons. Collectively, the data showing a depression-like phenotype should be strengthened by increasing the N and more robust statistical analysis.

Indeed, larger sample sizes can further strengthen the results. However, the present experiments were designed to use the minimum number of animals required to test predefined hypotheses, in accordance with the 3Rs principles. Additionally, all animals meeting predefined histological and experimental inclusion criteria were included, and there was no objective basis for exclusion of any animals in the supplementary data of FigS4B. The comparisons in FigS4B were planned a priori; therefore, correction for multiple comparisons would have been counterproductive as described above. We also used non-parametric statistical tests throughout the manuscript, providing a conservative strict statistical assessment.

Importantly, our conclusion does not rely on this supplementary assay and its significant result alone, but on convergent evidence from stress-induced MRR^{vGluT2} activation, ketamine sensitivity, bidirectional behavioral manipulation, and rescue of stress-induced deficits by neuronal inhibition. We therefore believe that the current data robustly support the involvement of MRR^{vGluT2} neurons in depression-related phenotypes.

While generally easy to read, the overall narrative and message could be more effective by reworking the rationales for transitions between sections of the Results. This reviewer became lost at points, as some statements lack precision.

We thank the reviewer for this comment. We carefully re-examined all Results subsections to determine whether additional transition or rationale sentences were needed. The manuscript had been intentionally organized so that each subsection begins with an experimental rationale or question and ends with a concise conclusion. Now we added further explanatory text to the manuscript and we hope that this organization will help readers follow the conceptual progression of the work.

The deeper histological assessment of MRR^{vGlut2} brain-wide connectivity compared to Szönyi et al. (2019) (Fig. S3) should include, at the very least, a graphed quantitative assessment of the different projections.

In response to this reviewer's request, we performed additional quantitative analyses of the MRR^{vGluT2} brain-wide projections. This analysis confirmed that the $MRR^{vGluT2\rightarrow LHb}$ neuronal population largely gives axon collaterals to the same brain regions as the broader MRR^{vGluT2} population. These results and corresponding figures (Fig. S3 G) have been added to the manuscript.

To my understanding, the negative state induced by mPFC–MRR activation (Figs. 7L–N) and by MRR^{vGlut2} activation (Figs. S1D–G) are demonstrated in different assays, i.e., place preference vs. auditory conditioning. The rationale for this design is missing.

These experiments were designed to test complementary aspects of the same pathway. Direct MRR^{vGluT2} neuronal activation was paired with an auditory cue to test whether activation of these neurons can generate an aversive memory that is later recalled by the cue. In contrast, $mPFC^{\rightarrow MRR}$ activation was tested in a place-preference paradigm to determine whether activation of the upstream cortical input is sufficient to induce an ongoing negative affective state. Thus, the two assays address related but distinct questions: whether the pathway can create a memory of a negative experience, and whether its upstream activation can directly evoke negative valence. The text was updated accordingly.

Minor Comments

The phrase “but the circuit described in the literature was incomplete” suggests that the authors now provide a complete description. This should be toned down to avoid overstatement.

We changed the text accordingly: "but the circuit described in the literature is still incomplete".

FigS1A: Absence of significance at conditioning seems to be driven by an outlier. Otherwise, conditioning data seems comparable to Recall with manipulation. This undermines the reported effect at Recall (Fig. 1D).

We thank the reviewer for this question. After re-examining the supplementary data, we identified an error in the presentation of the data. In the previous version, freezing values were inadvertently pooled across the whole trial rather than shown separately for baseline and shock periods. We have now corrected the relevant supplementary figure and table to accurately present shock-induced fear behavior during conditioning.

Regarding the apparent outlier, we did not find an objective basis for excluding this animal. Freezing behavior rarely shows a normal distribution, and therefore outlier definitions based on normally distributed data are not possible here. Moreover, this animal later showed normal fear behavior compared with other mice in the same group, arguing against exclusion on biological or technical grounds (see figure on the right).

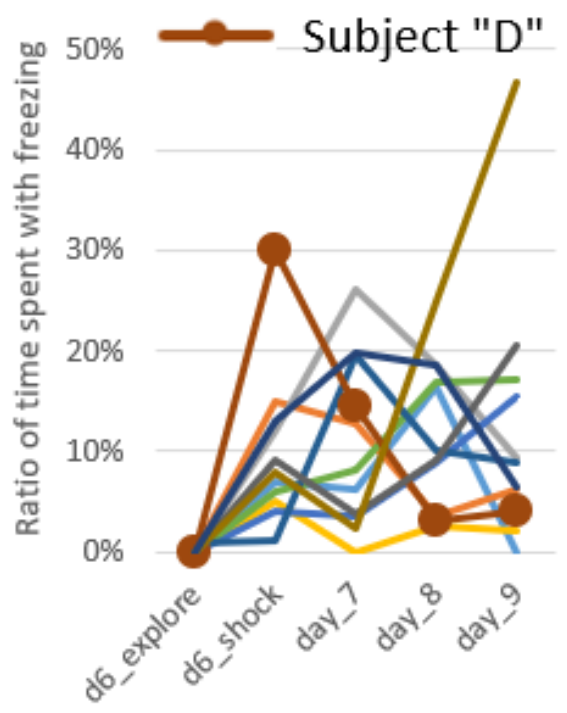

Importantly, the conclusion is unchanged if this animal is removed from the analysis. No significant group difference is detected during conditioning either with ($p = 0.2372$) or without ($p = 0.0910$; Mann–Whitney U test) this subject. Thus, the recall effect reported in Fig. 1D is not undermined by the conditioning data.

Fig. 3F-L: What is the rationale for inhibiting only half of the trial? Why not every other trial?

We divided each cue presentation into two equal segments to obtain sufficient paired light and no-light periods for statistical comparison within each unit. By splitting individual cue presentations, we could compare light and no-light periods at similar levels of fear recall. In addition, the order of light and no-light segments was alternated across cues to counterbalance any potential within-cue dynamics.

P7|Line 20-21: How does this fit to the fact that MRRvGlut2 neuron activity fades after 3 trials (Fig.2I)? Is the cellular activity reactivated?

Indeed, the significantly enhanced activity of $MRR^{\cdot vGluT2}$ neurons diminishes after the third cue presentation and becomes statistically not significant. However please note that the median values remain still higher at T4 and T5, Fig 2I. Additionally, Fig. 3I suggests that MRR^{vGluT2} neurons continue to significantly influence LHb activity at later trials as well. This is likely because the baseline firing of MRR^{vGluT2} neurons makes a significant contribution to LHb neuronal activity. Consequently, optogenetic inhibition of MRR^{vGluT2} neurons can reduce the excitatory drive onto LHb neurons even during later trials of fear recall.

Quantifications in Fig. 6 would be more accessible in a figure than in the legend.

Thank you, we have updated the figure accordingly.

Behavior for conditioning (e.g. Fig.9D-E, day 6) should be reported throughout the paper.

We thank the reviewer for this comment. We now present the freezing data from the conditioning periods for all experiments (Fig. S1 and Fig. S4).

Please also note that during re-evaluation of the data, we also noticed that in the experiment testing the role of $mPFC^{\rightarrow MRR}$ neurons in fear memory recall, one of the control cohort groups showed significantly lower freezing compared with the control animals of the other cohort. This indicated that pooling the cohorts was not appropriate. Without pooling, however, the sample size was insufficient. To address this issue, we repeated the same experiment using ArchT-mediated inhibition of $mPFC^{\rightarrow MRR}$ neurons. These new data are now presented in Fig. 9A–E and support the

same conclusion: MRR-projecting mPFC neurons are required for recalling negative experiences. The text was updated accordingly.

Reviewer #3 (Remarks to the Author):

The authors provided the impressive amount of data collected using a diverse range of neuroscientific techniques.

We thank the reviewer for the insightful evaluation of our work. Please find our point-by-point responses below.

However, despite this substantial effort, the manuscript's central narrative is unclear. It is difficult for the reader to distill a core takeaway message from the extensive results presented. This lack of focus is further exemplified by the inclusion of numerous figures that do not seem to directly align with the main theme promised by the title. A clear example of this disjointed narrative is found in Figure 4, which illustrates the function of MRR in a depression model. The manuscript fails to clarify the relevance of this finding to fear memory processing, which is the study's central theme. The authors should explicitly state and justify the connection between these two contexts.

We thank the reviewer for raising this point. We agree that the central conceptual message should be clear to all readers. The unifying focus of the manuscript is the role of MRR^{vGluT2} neurons in re-engaging the negative affective component of recalled experiences. This is directly relevant not only to adaptive fear memory recall, but also to pathological states in which negative experiences are persistently or maladaptively recalled, such as anxiety, PTSD, and depression. Thus, Fig 4 is not intended as a separate theme, but as a clinically relevant direct extension of the same circuit mechanism, controlled by the MRR^{vGluT2} neurons. Specifically, the first part of Fig 4 shows that MRR^{vGluT2} neuronal activity is altered in stressed animals and normalized by ketamine treatment, linking this cell population to a clinically relevant antidepressant-sensitive state. The second part shows that manipulating these neurons regulates depression-like behaviors, supporting the idea that this pathway contributes to maladaptive negative affect. Together, these data strengthen the main conclusion that MRR^{vGluT2} neurons are not merely involved in acute fear responses, but act as a broader hub for negative affective memory recall and updating that is the main message of the study. We have clarified this connection in the revised manuscript and ensured that each Results section begins with a clear rationale or question and ends with a concise conclusion to guide readers through the conceptual progression of the study.

In addition, the authors propose that the prefrontal-raphe-habenular circuit drives fear memory recall. While figure 1 and 2 show that inactivation of the MRR impairs fear memory recall, subsequent figures focus on the role of raphe-habenular circuit in depression-like behaviors or the involvement of mPFC-raphe projections in encoding negative valence. These data suggest that the observed fear memory recall impairments in figure 1, 2 may reflect disruptions in fear expression or aversive assessment, rather than memory recall per se. However, the schematic in figure S17 implies that MRR is involved in neither memory-related nor aversion-related process, further adding to the conceptual ambiguity. This lack of clear mechanistic framing weakens the overall coherence and significance of the manuscript's central argument.

We thank the reviewer for identifying this conceptually very important question. Indeed, our interpretation is that the prefrontal-raphe-habenular pathway does not encode the sensory or contextual content of the memory itself but (as this reviewer also suggests) is required to re-engage the aversive affective component associated with the recalled memory. In this sense, impaired

freezing after pathway inhibition reflects disruption of the negative affective/fear state normally reinstated during memory recall, rather than loss of the contextual or auditory memory trace itself. Accordingly, the data on MRR$^{\rightarrow LHb}$ function, depressive-like behaviors, and mPFC$^{\rightarrow MRR}$ neurons are intended to support one unified model: this pathway links memory-related forebrain circuits to brainstem–habenular valence circuits that reinstate negative affect during recall and updating. We agree that the previous Fig S17 did not convey this model clearly. We have therefore revised Fig. S17 and the corresponding text to explicitly confirm that MRR^{vGluT2} neurons participate in aversive affective recall and valence updating during fear memory retrieval.

Fundamentally, while the central premise of the study—that MRR is crucial for fear memory recall and the effort to identify its circuits—is novel and holds potential, the manuscript as presented does not convincingly argue that the findings represent a significant conceptual advance worthy of publication in Nature Communications. To be clear, this is not a critique of the data's quality, quantity, or the authors' commendable efforts. Rather, the issue lies with the manuscript itself, which fails to effectively frame the findings and articulate their broader impact. The paper currently reads more like a collection of interesting observations rather than a cohesive narrative that builds a compelling case for the significance of this circuit. For these reasons, I cannot recommend the manuscript for publication in Nature Communications in its current form. However, should the authors undertake a substantial revision to build a more focused and compelling narrative around their most significant findings, the work could represent a valuable contribution to the field.

We thank the reviewer for recognizing the quality and extent of our data and the effort invested in this study, and we also appreciate their constructive criticism. We agree that the central conceptual advance of the manuscript must be clear. We have therefore revised the manuscript to build a more focused narrative around our main findings.
In the revised manuscript, we now better frame the mPFC→MRR→LHb pathway as a previously unrecognized causal substrate for a recall-specific operation. In contrast to the canonical tripartite fear circuit, which primarily organizes contextual and sensory components of fear memories, our data suggest that the mPFC→MRR→LHb pathway is required to reinstate and flexibly scale the aversive emotional component of a retrieved memory. This interpretation is supported by the necessity of this pathway for both contextual and cued fear recall, the graded activity of MRR^{vGluT2} neurons with memory aversiveness, and the bidirectional effects of this circuit on extinction and depression-like behaviors. Thus, impaired fear behavior after pathway inhibition is best interpreted as disruption of the negative affective/fear state normally reinstated during recall, rather than as loss of the contextual or auditory memory trace itself. This framing also provides a unified rationale for the different parts of the study. The MRR→LHb experiments define a brainstem–habenular output pathway for aversive affect; the depression-related experiments show that the same neuronal population is engaged in maladaptive negative affective states; and the mPFC→MRR experiments identify a cortical input through which memory-related forebrain circuits can recruit this brainstem valence system during recall.
We have also clarified why fear learning and recall should be considered separately. Learning requires integration of ongoing aversive sensory input with contextual or cued information, whereas recall reactivates an existing representation and can update its affective value without the original aversive stimulus.

To address the reviewer's concern, we revised the Introduction and Discussion and updated Fig. S17 to explicitly present this unified model. We believe these revisions now clarify the work as a cohesive conceptual advance: the identification of a brainstem-centered circuit mechanism through which forebrain memory networks reinstate the negative affective component of fear memories.

Reviewer #4 (Remarks to the Author):
I co-reviewed this manuscript with one of the reviewers who provided the listed reports. This is part of the Nature Communications initiative to facilitate training in peer review and to provide appropriate recognition for Early Career Researchers who co-review manuscripts.
