# Supplementary material for: Prefrontal-raphe-habenular circuit drives fear memory recall and updating
Source: Nat Commun. 2026 Aug 12;17:9686. doi: 10.1038/s41467-026-76574-5 (PMC13558683; doi:10.1038/s41467-026-76574-5)
Supplement: Supplementary file 3 [file 41467_2026_76574_MOESM3_ESM.pdf]

nature portfolio

Corresponding author(s): Gábor Nyiri

Last updated by author(s): Jun 29, 2026

# Reporting Summary

Nature Portfolio wishes to improve the reproducibility of the work that we publish. This form provides structure for consistency and transparency in reporting. For further information on Nature Portfolio policies, see our Editorial Policies and the Editorial Policy Checklist.

## Statistics

For all statistical analyses, confirm that the following items are present in the figure legend, table legend, main text, or Methods section.

| n/a | Confirmed | |
|---|---|---|
| ☐ | ☒ | The exact sample size (*n*) for each experimental group/condition, given as a discrete number and unit of measurement |
| ☐ | ☒ | A statement on whether measurements were taken from distinct samples or whether the same sample was measured repeatedly |
| ☐ | ☒ | The statistical test(s) used AND whether they are one- or two-sided<br>*Only common tests should be described solely by name; describe more complex techniques in the Methods section.* |
| ☒ | ☐ | A description of all covariates tested |
| ☐ | ☒ | A description of any assumptions or corrections, such as tests of normality and adjustment for multiple comparisons |
| ☐ | ☒ | A full description of the statistical parameters including central tendency (e.g. means) or other basic estimates (e.g. regression coefficient) AND variation (e.g. standard deviation) or associated estimates of uncertainty (e.g. confidence intervals) |
| ☐ | ☒ | For null hypothesis testing, the test statistic (e.g. *F*, *t*, *r*) with confidence intervals, effect sizes, degrees of freedom and *P* value noted<br>*Give P values as exact values whenever suitable.* |
| ☒ | ☐ | For Bayesian analysis, information on the choice of priors and Markov chain Monte Carlo settings |
| ☒ | ☐ | For hierarchical and complex designs, identification of the appropriate level for tests and full reporting of outcomes |
| ☒ | ☐ | Estimates of effect sizes (e.g. Cohen's *d*, Pearson's *r*), indicating how they were calculated |

*Our web collection on statistics for biologists contains articles on many of the points above.*

## Software and code

Policy information about availability of computer code

| | |
|---|---|
| Data collection | Zeiss Axioplan2 microscope, Zeiss Axioscan Z1 microscope, 3DHISTECH Pannoramic Midi II automatic slide scanner, Nikon A1R confocal laser-scanning microscope, Hitachi 7100 electron microscope, FEI Apreo field emission gun (FEG) SEM, NIS-Elements AR 4.3, Noldus Ethovision 15.0, Open Ephys, Custom-made freezing detection head-mounted sensor (movement data collection), Doric Neuroscience Studio 5.4.1.23 for fiber photometry, Neurolucida System |
| Data analysis | NIS-Elements AR 4.3, Adobe Photoshop CS6, StatSoft STATISTICA 13.1, Igor Pro 9, Spyking Circus software, Phy software, MATLAB-based custom made freezing analysis software, Custom-written MATLAB scripts for fiber photometry (https://doi.org/10.5281/zenodo.21026721); Clampfit (v10.7, Molecular Devices) and SutterPatch (v2.3.1, running under IgorPro8.04, Sutter Instrument software, and custom-written Python scripts (Python 3.11) for in vitro electrophysiological experiments; Fragment Analyzer, Illumina's bcl2fastq2 (v2.20), fastqc, Trimmomatic, STAR aligner for genetic analysis; Neurolucida System, ezTrack software, Deeplabcut 3.0.0rc13, movement Python library 0.15.0 |

For manuscripts utilizing custom algorithms or software that are central to the research but not yet described in published literature, software must be made available to editors and reviewers. We strongly encourage code deposition in a community repository (e.g. GitHub). See the Nature Portfolio guidelines for submitting code & software for further information.

## Data

Policy information about availability of data

All manuscripts must include a data availability statement. This statement should provide the following information, where applicable:

- Accession codes, unique identifiers, or web links for publicly available datasets
- A description of any restrictions on data availability
- For clinical datasets or third party data, please ensure that the statement adheres to our policy

The RNA sequencing data generated in this study have been deposited in the NCBI Gene Expression Omnibus (GEO) database under accession code GSE300145 [https://www.ncbi.nlm.nih.gov/geo/query/acc.cgi?acc=GSE300145]. The RNA sequencing data generated in this study are publicly available through GEO. All other relevant data generated in this study are provided in the manuscript and its Supplementary Information/Source Data file.

## Research involving human participants, their data, or biological material

Policy information about studies with human participants or human data. See also policy information about sex, gender (identity/presentation), and sexual orientation and race, ethnicity and racism.

| | |
|---|---|
| Reporting on sex and gender | N/A |
| Reporting on race, ethnicity, or other socially relevant groupings | N/A |
| Population characteristics | N/A |
| Recruitment | N/A |
| Ethics oversight | N/A |

Note that full information on the approval of the study protocol must also be provided in the manuscript.

# Field-specific reporting

Please select the one below that is the best fit for your research. If you are not sure, read the appropriate sections before making your selection.

[x] Life sciences [ ] Behavioural & social sciences [ ] Ecological, evolutionary & environmental sciences

For a reference copy of the document with all sections, see nature.com/documents/nr-reporting-summary-flat.pdf

# Life sciences study design

All studies must disclose on these points even when the disclosure is negative.

| | |
|---|---|
| Sample size | For all experiments sample sizes were determined in an empirical way to minimize the number of animals used, but to allow a strong enough statistical power. |
| Data exclusions | In case of all experiments, mice were excluded only if there was no sign of virus injection or projection fibers in the targeted area or if the position of the optic fibers or silicon probes were mistargeted. |
| Replication | All experiments included in the manuscript were successfully replicated several times on different days in several animals. |
| Randomization | Mice were allocated into experimental groups randomly, and behavioral experiments with control and experimental animals were carried out in a random order. |
| Blinding | In optogenetic behavioral and anatomical experiments, the experimenters were blinded to the conditions and history of the mice during the experiments. The analysis of behavioral, fiber photometry, in vitro and in vivo electrophysiological experiments were automated. |

# Reporting for specific materials, systems and methods

We require information from authors about some types of materials, experimental systems and methods used in many studies. Here, indicate whether each material, system or method listed is relevant to your study. If you are not sure if a list item applies to your research, read the appropriate section before selecting a response.

## Materials & experimental systems

| n/a | Involved in the study |
|---|---|
| | [x] Antibodies |
| [x] | Eukaryotic cell lines |
| [x] | Palaeontology and archaeology |
| | [x] Animals and other organisms |
| [x] | Clinical data |
| [x] | Dual use research of concern |
| [x] | Plants |

## Methods

| n/a | Involved in the study |
|---|---|
| [x] | ChIP-seq |
| [x] | Flow cytometry |
| [x] | MRI-based neuroimaging |

# Antibodies

| | |
|---|---|
| Antibodies used | All antibodies used in this study listed in TableS6-S7 |
| Validation | All antibodies used in this study listed in TableS6-S7 |

# Animals and other research organisms

Policy information about studies involving animals; ARRIVE guidelines recommended for reporting animal research, and Sex and Gender in Research

| | |
|---|---|
| Laboratory animals | We used vGluT1-iRES-Cre (Stock No.: 023527), vGluT2-iRES-Cre (Stock No.: 028863), PV-Cre (Stock No.: 017320) and Fos2a/iCre-ERT2 (Stock No.: 030323) mice. All Cre-mice were from The Jackson Laboratory. We used C57Bl/6J mice from Charles River. We used adult (at least 50 days-old) mice from both sexes in our experiments. Mice had access to food and water ad libitum and were housed in a vivarium (3-5 mice/cage) until used in experiments. All mice were maintained on a normal 12h light-dark cycle, with experiments performed during the light phase of the cycle. Animal holding rooms were climate-controlled, maintaining a temperature range, and humidity range of 21°C, 35% to 25°C, 65%.<br>In case of the in vivo electrophysiology experiments we used 8–16-week-old vGluT2-iRES22 Cre mice. Ten of those were used for optogenetical tagging and 8 for lateral habenula-median raphe experiments. Mice were individually housed for at least 14 days before electrophysiological experiments.<br>In case of the freely moving MRRvGluT2 fiber photometry experiments, we used 8 male vGluT2-iRES-Cre mice. Only heterozygous (Cre/wild type) mice were used for experiments and were backcrossed to a C57BL/6J background for more than seven generations. Mice were individually housed for at least 14 days before starting behavioral paradigms and had access to a shelter and a running wheel.<br>We used adult (46-48 days old) male and female Wistar rats also from Charles River.<br>We used the intact brainstems of three adult female rhesus macaque monkeys (Macaca mulatta) that were previously studied by Rovo et al. in 2012. |
| Wild animals | No wild animals were involved in the study. |
| Reporting on sex | We used mice and from both sexes in our experiments. We used adult male and female Wistar rats and one female rhesus macaque monkey. |
| Field-collected samples | No field-collected samples were involved in the study. |
| Ethics oversight | Freely moving fiber photometry experiments were performed in accordance with institutional guidelines at the Friedrich Miescher Institute for Biomedical Research and were approved by the Veterinary Department of the Canton of Basel-Stadt. All other experiments were performed in accordance with the Institutional Ethical Codex and the Hungarian regulations on animal research (Act XXVIII of 1998, Government Decree 40/2013), in line with the EU Directive 2010/63/EU. The Animal-welfare Body of the Institute of Experimental Medicine and the Government Office of Pest County authorized the experiments at project number PE/EA/00063-5/2022 and PE/EA/00513-6/2025. Experiments on rhesus macaque monkeys tissues were performed according to the ethical guidelines of the Institute of Experimental Medicine and Institute of Physiology of the Hungarian Academy of Sciences and approved by the Ethical Committee (Approval 22.1/77/001/2010). |

Note that full information on the approval of the study protocol must also be provided in the manuscript.

## Plants

| | |
|---|---|
| Seed stocks | N/A |
| Novel plant genotypes | N/A |
| Authentication | N/A |
